# Cost-effective assembly of the African wild dog (*Lycaon pictus*) genome using linked reads

**DOI:** 10.1093/gigascience/giy124

**Published:** 2018-10-22

**Authors:** Ellie E Armstrong, Ryan W Taylor, Stefan Prost, Peter Blinston, Esther van der Meer, Hillary Madzikanda, Olivia Mufute, Roseline Mandisodza-Chikerema, John Stuelpnagel, Claudio Sillero-Zubiri, Dmitri Petrov

**Affiliations:** 1 Program for Conservation Genomics, Department of Biology, 385 Serra Mall, Stanford University, Stanford, CA, 94305, USA; 2 Department of Integrative Biology, 3040 Valley Life Science Building, University of California, Berkeley, CA, 94720-3140, USA; 3 Painted Dog Conservation, PO Box 72, Dete, 00263, Zimbabwe; 4 The Zimbabwe Parks & Wildlife Management Authority, Corner Sandringham & Borrowdale Roads, Botanical Gardens. Causeway, Harare, 00263, Zimbabwe; 5 10x Genomics, Inc., 7068 Koll Center Pkwy #401, Pleasanton, CA, 94566, USA; 6 Wildlife Conservation Research Unit, Zoology, University of Oxford, The Recanati-Kaplan Centre, Abingdon Road, Tubney House, Tubney, UK014

**Keywords:** conservation genomics, 10x Genomics Chromium, African wild dog, *Lycaon pictus*, *de novo* Assembly

## Abstract

**Background:**

A high-quality reference genome assembly is a valuable tool for the study of non-model organisms. Genomic techniques can provide important insights about past population sizes and local adaptation and can aid in the development of breeding management plans. This information is important for fields such as conservation genetics, where endangered species require critical and immediate attention. However, funding for genomic-based methods can be sparse for conservation projects, as costs for general species management can consume budgets.

**Findings:**

Here, we report the generation of high-quality reference genomes for the African wild dog (*Lycaon pictus*) at a low cost (<$3000), thereby facilitating future studies of this endangered canid. We generated assemblies for three individuals using the linked-read 10x Genomics Chromium system. The most continuous assembly had a scaffold and contig N50 of 21 Mb and 83 Kb, respectively, and completely reconstructed 95% of a set of conserved mammalian genes. Additionally, we estimate the heterozygosity and demographic history of African wild dogs, revealing that although they have historically low effective population sizes, heterozygosity remains high.

**Conclusions:**

We show that 10x Genomics Chromium data can be used to effectively generate high-quality genomes from Illumina short-read data of intermediate coverage (∼25x–50x). Interestingly, the wild dog shows higher heterozygosity than other species of conservation concern, possibly due to its behavioral ecology. The availability of reference genomes for non-model organisms will facilitate better genetic monitoring of threatened species such as the African wild dog and help conservationists to better understand the ecology and adaptability of those species in a changing environment.

## Background

Major population declines have been observed in vertebrate groups over the past several hundred years, primarily due to anthropogenic change [1]. This decline has resulted in extinction rates unprecedented in recent history [1, 2]. The conservation of extant species will require major efforts in restoring and preserving habitat, along with protection, management, and investment by local stakeholders. While, by definition, all species of conservation concern exist as small populations, populations generally still retain genetic variation that was generated and maintained when population sizes were much larger.

The historic genetic variation contains signals of demographic history, gene flow, and natural selection, which can inform efforts toward the long-term survival of species. In addition to signals of a species history, genetic information can be used to uncover important contemporary or very recent events and processes. Genetic markers can be used to track individual movement across landscapes either indirectly by measuring relatedness or directly by genotyping scat or hair left by an individual as it moves. Additionally, the identification and assignment of individuals through genotyping can be an important tool for law enforcement to assign contraband and confiscated materials to their geographic origin [3]. Conservationists can also use fine-grained measurements of reproductive success along with genotypes and environmental variables to gather a detailed understanding of the factors contributing to or limiting population growth, such as inbreeding depression. Taken together, genomic tools are poised to have a major contribution to conservation [4, 5].

**Figure 1:**
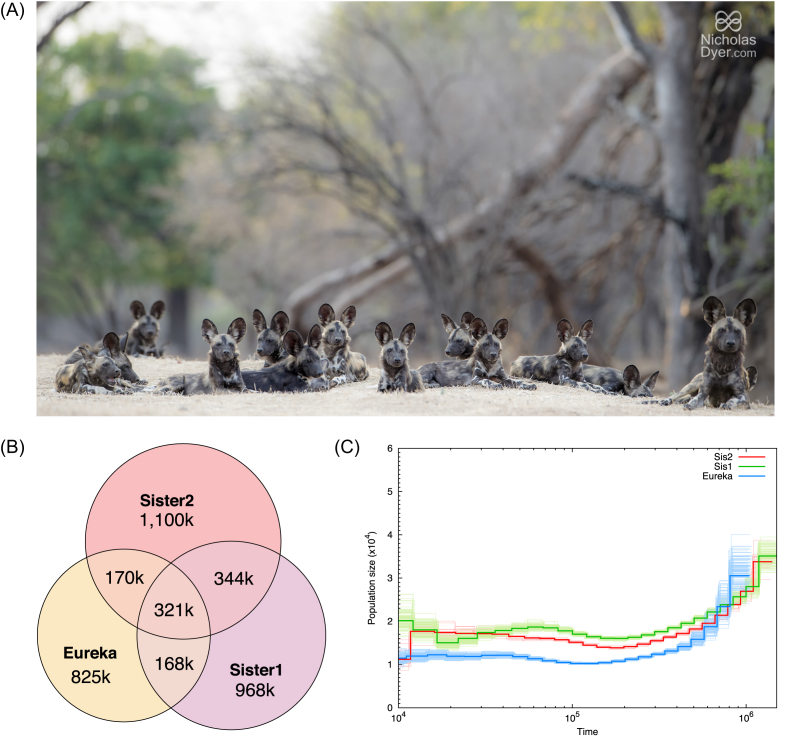
**(A)** Pack of African wild dogs. **(B)** Shared heterozygous sites between the three *de novo* assemblies (calculated using a posterior cutoff of 0.99). More of the heterozygous sites are shared between the two sisters than between either sister and Eureka. **(C)** Reconstruction of the individuals’ demographic history using the PSMC program. Bootstrap replicates are plotted in lighter colors. Time is in years before present.

The African wild dog, also known as the African painted dog or Cape hunting dog (*Lycaon pictus*), is a medium-sized (18–34 kg), endangered carnivore that lives in scattered populations in sub-Saharan Africa (Fig. 1A). The species is a surviving member of a lineage of wolf-like canids, including other species such as the Ethiopian wolf and the dhole [6]. Wild dogs have been subject to intense recovery efforts across their range [7, 8], but their global population is decreasing. It is estimated that only 6,600 adult wild dogs remain in 39 subpopulations [9]. The primary reasons for the species' population decline include habitat loss and fragmentation, as well as anthropogenic mortality (e.g., snaring, persecution, road kills, exposure to infectious diseases from domestic dogs) when they range beyond the borders of protected areas [7, 8, 10]. Due to their large ranges and low population densities, African wild dogs are more susceptible to these threats than most other carnivore species [8]. In addition, their complex social system and susceptibility to Allee effects appears to increase the species extinction risk [11, 12]. The dogs are obligate cooperative breeders that form packs consisting of an alpha male and female, their adult siblings, and pups and subadults from the dominant pair [13]. Subadults that have reached reproductive age disperse in single sex groups and form new packs by joining dispersing groups from the opposite sex [14]. Pack members rely on each other for hunting, breeding, and defense against natural enemies; pack size has been found to be a significant factor in determining hunting and breeding success [13, 15, 16]. When pack size becomes critically low, this dependence on helpers increases the risk of pack extinction and reduces the number of successful dispersals ([12], but see [17]).

Prior genetic studies on wild dogs using a combination of mitochondrial, microsatellite, and Major Histocompatibility Complex (MHC) markers have resulted in varying estimates of the start of the species decline on the African continent [18, 19]. Consistent with expectation, the data show strong structuring among populations due to habitat fragmentation and isolation, as well as low genetic diversity within populations [19, 20]. For species that are experiencing such rapid and alarming declines, estimates that are particularly important for management decisions, such as effective population size, inbreeding, and local adaptation, are greatly improved by the use of whole-genome methods. Recently, Campana and colleagues [21] sequenced low-coverage genomes of two African wild dog individuals from Kenya and South Africa, respectively, to investigate demographic history and signatures of selection of these two separate populations. By mapping these data to the domestic dog genome, they discovered approximately 780,000 single-nucleotide polymorphisms (SNPs) between their two individuals that could be used to develop SNP typing for the two populations. However, given the low coverage of their genomes (5.7–5.8x average coverage) and the small number of individuals sequenced, additional sequencing will be needed to verify the authenticity of those SNPs. Further, important structural variation can be overlooked when mapping against a reference genome from a different genus, and mapping can be hindered if the divergence is high between the sample and the reference (see, e.g., [22]). The groups containing the African wild dog and the domestic dog are estimated to have split approximately 2.5–4 million years ago (Mya); furthermore, the domestic dog has undergone significant genomic selection in recent times [23–25].

Despite the ever-declining cost to sequence DNA, the routine use of genomic approaches in conservation is still far from a reality. One of the major remaining barriers is the lack of reference genomes for species of conservation concern. Generating a *de novo* reference genome generally requires the sequencing and assembly of billions of base pairs that make up a genome. The first mammalian genome (human) required a massive collaboration among hundreds of scientists and nearly $3 billion (1990–2001; [26, 27]). Fortunately, the cost to sequence DNA is now low enough that every base-pair in a typical mammalian genome can be sequenced to high coverage for a few thousand dollars. However, these low-cost sequencing methods produce very short sequences of 150–300 base-pairs in length (for a review on sequencing methods, see [28]). Because large proportions of typical mammal genomes consist of repetitive sequences, it has been challenging to obtain complete or highly contiguous genomes using only these short sequences. In order to achieve higher continuity, more elaborate and expensive library preparation or alternative sequencing technologies have to be used [28, 29]. Among others, these include mate-pair (MP) libraries; chromatin folding-based libraries, such as cHiCago [30] or HiC [31]; and long-read sequencing technologies, such as Pacific Biosciences and Oxford Nanopore Technology. While the resulting genomes can show high continuity, those methods substantially increase the costs of sequencing projects and thus can hinder the generation of genomes for conservation biology purposes.

Here, we report the use of the Chromium system developed by 10x Genomics [32], a genomic library preparation technique that facilitates cost-effective assemblies using short sequencing reads, to assemble three African wild dog genomes. In brief, the 10x Genomics Chromium system is based on dilution of high-molecular-weight (HMW) DNA. It uses as little as 1 ng of input DNA, which is well suited for a variety of applications. During library preparation, gel beads, so-called GEMs, are mixed with DNA and polymerase for whole-genome amplification. Each gel bead has primer oligos (44 nt long) attached to its surface. These contain a priming site (22 nt partial R1), a 16-nt barcode region, and a 6-nt *N*-mer region that binds to different places on the original DNA fragment. The low amount of input DNA ensures that each gel bead only binds a single (up to ∼100 kb) DNA fragment. In the next step, amplification of short reads along the original DNA fragment is performed within each gel bead. In most cases, this amplification results in spotted read coverage along the fragment. However, all reads from a respective GEM contain identical bar codes and can later be assigned to groups originating from the same DNA molecule. The information about which molecule of DNA the sequence originated from greatly increases the ability to identify the location of repetitive sequences. The library is then sequenced on an Illumina platform, and the raw read data are assembled by the 10x Genomics Supernova assembler. The data produced also can be phased, presenting another potentially useful addition to genome assemblies.

We *de novo* assembled three African wild dog genomes using the 10x Genomics Chromium platform to investigate whether this technology is suitable for conservation genomic purposes. For any endangered species, a genome can enable studies with the potential for large conservation impacts, but high-quality genomes have historically been costly or impossible due to the sampling requirements and analysis. Thus, for an assembly to be a practical component of many conservation projects, the technology needs to be cost-effective and user-friendly. We test the 10x Genomics Chromium based assemblies for reproducibility, continuity, conserved gene completeness, and repetitive content, as compared to the previously published domestic dog genome [33] and several other genomes built with various technologies. We further estimate heterozygosity of the individuals and within the phased data from the 10x technology and estimate historical effective population size from each genome.

## Data Description and Analyses

### Assembly of the African wild dog genome

Using 10x Genomics Chromium technology, we generated DNA libraries for three African wild dog individuals, two of which were collected from a wild pack in Hwange National Park, Zimbabwe, and are sisters from the same litter born in June 2013 (identified as sister 1 and sister 2; additional information can be found in Supplementary Appendix S1), and a third unrelated individual from the Endangered Wolf Center, Eureka, Missouri (identified as Eureka). A summary of the assembly statistics output by the Supernova assembler can be found in Table 1 (detailed statistics for each genome assembly can be found in Supplementary Table S1). We generated ∼1.2 billion paired-end (PE) reads for sister 1, ∼0.8 billion reads for sister 2, and ∼0.4 billion reads for Eureka. We then used the reads to assemble each genome using the 10x Genomics Supernova assembler (as explained in [34]). The mean input DNA molecule length reported by the Supernova assembler was 19.91 kb for sister 1, 196,77.03 kb for sister 2, and 52.00 kb for Eureka. All three assemblies corroborate a genome size of approximately 2.3 Gb, which is similar to that of the domestic dog (2.4 Gb; [33]). These three assemblies together constitute the first reported *de novo* assemblies for the African wild dog species.

**Table 1:** Assembly statistics.

		Sister 1	Sister 2	Eureka
Input	Reads (m)	1200	801.56	427.6
	Average coverage	69	46	25
	Mean molecule size (kb)	19.91	77.03	52.00
Contig	N50 (kb)	61.34	83.47	50.15
	Longest (kb)	524.60	615.40	450.50
	Number (k)	78.62	68.64	108.00
Scaffold	N50 (mb)	7.91	21.34	15.31
	Longest (mb)	43.96	69.63	41.67
	Number (k)	11.78	17.64	25.78
Total size (gb)	Scaffolds ≥ 10 kb	2.27	2.26	2.20
	Scaffolds ≥ 500 bp	2.34	2.40	2.42

Assembly statistics for the three African wild dog genomes reported by the Supernova assembler. Coverage was assessed using SAMtools depth.

The sister 1 assembly resulted in a 61.34 kb contig and 7.91 Mb scaffold N50; the sister 2 assembly achieved 83.47 kb contig and 21.34 Mb scaffold N50; and the Eureka assembly had 50.15 kb contig and 15.31 Mb scaffold N50 (Table 1). While the scaffold N50s of these three 10x genomes are are smaller than the ones from the most recent dog genome (267 kb and 45.9 Mb, respectively), they are still larger than most mammalian genomes assembled that used only short-read data (see, e.g., [35]). A recent *de novo* assembly of a wild wolf using Illumina MP libraries of varying insert size resulted in a similar contig N50, but much lower scaffold N50 measurements than our results (Supplementary Table S2; [36]). Interestingly, despite the molecule size being the highest for sister 2, the highest percent phased data was obtained by Eureka (52.54% compared to 40.1%; Supplementary Table S1).

### Conserved genes

The program Benchmarking Universal Single-Copy Orthologs (BUSCO) uses highly conserved single-copy orthologous genes from several different taxa and groups to test assemblies (both genomic and transcriptomic) for gene completeness, fragmentation, and absence as an indicator of assembly quality. Using BUSCO v2 on our assemblies, we found that the most continuous assembly, sister 2, completely recovered 95.1% of conserved genes (Mammalia gene set; Table 2). Sister 1 and Eureka recovered 95.4% and 93.3% of complete conserved genes, respectively. Using the same analysis, we found 95.3% of complete conserved genes in the latest dog assembly (canFam3.1; [33]). This indicates that although the domestic dog assembly is more continuous overall, our assemblies recover nearly the same or even higher numbers of conserved genes. Surprisingly, sister 1 had the fewest missing genes out of all the assemblies assessed, despite lower continuity than sister 2. We also ran BUSCO on the Hawaiian monk seal genome, generated through the combination of 10x Genomics Chromium and Bionano Genomics Irys data, and found it recovered 94.6% of conserved genes using BUSCO [37]. This suggests that using Bionano in addition to 10x does not greatly improve the reconstruction of the gene regions. However, the Hawaiian monk seal genome has a scaffold N50 of approximately 28 Mb, so Bionano may improve the overall assembly continuity compared to 10x Genomics alone. The low-coverage genomes from Campana et al. achieved a BUSCO score of 92.8% for the individual from Kenya and 94.8% for the individual from South Africa [21]. The wolf genome also scored similarly (94.8%) [36].

**Table 2:** Conserved gene statistics.

Assembly	Species	Complete	Single copy	Duplicated	Fragmented	Missing	Total searched
Sister 1	*L. pictus*	3,914	3,875	39	102	88	4,104
Sister 2	*L. pictus*	3,903	3,845	58	107	94	4,104
Eureka	*L. pictus*	3,829	3,789	40	169	106	4,104
canFam3.1	*C. familiaris*	3,910	3,857	53	98	96	4,104
Kenya	*L. pictus*	3,849	3,823	26	136	119	4,104
South Africa	*L. pictus*	3,892	3,867	25	104	108	4,104
Wolf	*C. lupus*	3,890	3,849	41	110	104	4,104
Hawaiian monk seal	*Neomonachus schauinslandi*	3,881	3,833	48	118	105	4,104

Results of the BUSCO v2 gene annotation from three African wild dog genome assemblies, canFam3.1, low-coverage wild dog genomes [21], the recently published wolf genome [36], and the Hawaiian monk seal genome [37].

### Repeat annotation

We identified repetitive regions of the genome to discern how well these complex areas were assembled by the 10x Genomics Chromium technology. We found that for all three wild dog assemblies, total repeat content was determined to be within 3% of one another, which indicates consistency among assemblies from a single species (Supplementary Table S3). No single repeat category was disproportionately affected during repeat annotation of the three genomes, which suggests that assembly quality was likely the most influential factor. Furthermore, repeat content of all wild dog assemblies was qualitatively similar to canFam3.1 [33] and the wolf genome [36], likely due to recent common ancestry between the two groups [23–25].

### Gene annotation

Genome annotation resulted in very similar numbers of annotated genes between all three African wild dog individuals and the domestic dog [33]. Annotations ranged from 20,649 (sister 2) to 20,946 (sister 1) genes (Supplementary Table S4). Through detecting orthologous genes between individuals and paralogous genes within individuals, we found 12,617 one:one orthologs present in all three individuals and 6,462 one:one orthologs in two of the three individuals. We found 268 multicopy genes present in all three individuals and 37 total not present in single individuals, likely due to their coverage differences (10 were missing in sister 1, 13 in sister 2, and 14 in Eureka). Overall, the number of annotated genes was comparable to those found in the domestic dog genome and the wolf genome (Supplementary Table S4; [33, 36]).

### Variant rates

We found a high number of heterozygous sites to be shared between all three individuals (321 k; here, we report the heterozygous sites called using a posterior probability cutoff of 0.99; Supplementary Fig. S2A). As expected, sister 1 and sister 2 share more heterozygous sites (344 k) than either sister with Eureka (168 k and 170 k for sister 1 and sister 2, respectively). Each individual shows a high number of singletons (heterozygous sites only found in one individual), with sister 2 showing the highest number (1,100 k), followed by sister 1 (968 k) and Eureka (825 k). Even if we include the two low-coverage genomes from Campana et al. [20], we find a high number of shared heterozygous sites between all individuals (134 k; Supplementary Fig. S2B). We see a higher number of singletons in these two individuals, most likely due to the lower reliability of the genotype calls caused by the low-coverage data (false positives caused by sequencing errors). We estimated a per site heterozygosity of 0.0008 to 0.0012 for sister 1, 0.0009 to 0.0012 for sister 2, and 0.0007 to 0.001 for Eureka using posterior cutoffs for genotype calls from 0.95 to 1 in ANGSD (Supplementary Fig. S1C). As can be seen in Supplementary Fig. S2, except for a posterior probability cutoff of 1 where sister 1 shows the highest heterozygosity, sister 2 always shows the highest, sister 1 the second highest, and Eureka the lowest heterozygosity. Interestingly, Eureka shows a lower heterozygosity than the other two assemblies, even though its parents are thought to have originated from different localities (Supplementary Text S1). With more stringent filtering, we likely could improve the heterozygosity estimates for the low-coverage individuals, but we did not investigate this further and maintained our methods across datasets for comparative purposes.

We did not see any major difference between heterozygosity estimates from repeat-masked and unmasked genomes [38]. The Supernova software estimated a heterozygous position every 2.6 kb, 3.1 kb, and 7.14 kb for sister 1, sister 2, and Eureka, respectively (Supplementary Table S5). On the contrary, estimates based on genotype calls using ANGSD showed much more frequent heterozygous positions (850 bp–1.2 kb, 814 bp–1.1 kb, and 999 bp–1.5 kb, depending on the posterior cutoff used; Supplementary Table S5). Overall, our estimates show that while being heavily threatened, African wild dogs seem to still retain a relatively high within-individual heterozygosity relative to other endangered species that have been estimated, such as those in the cheetah or the Amur tiger (>0.0005, 0.0005; [39]) or in the island grey fox (>0.0005; [40]). Additionally, the estimates here are comparable to those from several gray wolf individuals (0.0009–0.0012; [36]).

We also examined the phased data and its effect on heterozygosity estimates for one individual, sister 2. We find that the estimates are relatively consistent between both the pseudohaplotypes and the merged pseudohaplotype produced by the Supernova software (Supplementary Table S5) [38].

### Demographic history

We estimated demographic history using the program PSMC [41]. Our results show demographic trends that are similar to those reported by Campana et al. [21]; however, we observe declines beginning just over 1 Mya, as opposed to approximately 700,000 years ago (Fig. 1C). From 1 million to 120,000 years ago, the population size steadily declines, resulting in a predicted N_e_ of approximately 1,000–2,000 individuals. During the remainder of the African wild dog history, there are some small effective population size estimate fluctuations.

We also infer similar population histories from the genomes of the two sisters from Zimbabwe and, furthermore, show very little difference between the inferred history of the third individual, Eureka (Fig. 1C). This may be because the populations were formerly continuous and share their ancestral population history; however, further analyses would be required to disentangle these hypotheses. We also do not detect additional large fluctuations as noted by Campana et al. [21]; more high-coverage genomes from across populations would be needed to confirm that these do not exist, since our individuals are from populations that are distinct from those previously tested. Furthermore, population structure and short-term demographic incidents (e.g., populations bottlenecks) can affect PSMC estimations of historic population sizes [42]. In addition, the assumed mutation rate and generation times can have large effects on the resulting estimates. However, the data consistently reinforce that African wild dogs have existed at relatively low population sizes for a long time.

## Discussion

### Assembly continuity and quality

All three African wild dog assemblies produced with 10x Genomics Chromium data showed high continuity, high recovery rates of conserved genes, and expected proportions of repetitive sequence overall. The assembly for sister 2, which has the highest mean molecule length, is also the most continuous (contig N50: 83.47 kb, scaffold N50: 21.34 Mb; Table 1). Interestingly, the sister 1 genome has a higher contig N50 (61.34 kb) than Eureka (50.15 kb) but a lower scaffold N50 (7.91 Mb and 15.31 Mb, respectively). This may indicate that input molecule length is a key factor for scaffolding, while coverage is a key factor for contig assembly; indeed, input DNA quality is noted as the most common cause of failed or substandard assemblies [43]. Furthermore, the percent of the genome able to be phased across genomes did not correspond to input molecule length (Supplementary Table S1). More work is needed in order to determine the accuracy of the phased data and the wet lab methods and/or assembly parameters that influence these inferences.

Despite having the highest continuity of all three assemblies, sister 2 did not show the highest BUSCO completeness scores (see Table 2), although the differences were minor (with 95.1% complete BUSCOs compared to 95.4% for sister 1). Sister 1 achieved the highest BUSCO scores, even compared to the latest domestic dog genome assembly (CanFam3.1 [33]; 95.2%), which has three times higher contig N50 and an almost six times higher scaffold N50. The high scores are remarkable for the limited number of reads used for the assemblies (as low as 25x coverage). As expected, sister 2, which showed the highest continuity, also had the highest repeat content (see Supplementary Table S3). All three assemblies resulted in similar repeat contents in terms of repeat composition as well as overall percentage (within 3% of each other), with the most continuous assembly (sister 2) showing the highest number of repeats. Repeat composition in the African wild dog genomes was also similar to that of the domestic dog and the wolf [33, 36].

All assemblies yielded similar amounts of genes, with sister 1 showing the highest number (see Supplementary Table S4), which reflect its BUSCO scores. Closer investigations of one to one and one to many orthologs also showed a very good agreement between annotations obtained from all three individuals. The numbers of annotated genes for all three African wild dogs were similar to those calculated for the latest domestic dog assembly and wolf genome assembly [33, 36].

### 10x Genomics Chromium system: feasibility and caveats

Most mammal genomes published in the last several years use a mixture of PE and multiple MP Illumina libraries (e.g., [35] and [44]). While often resulting in good continuity (e.g., [44] or [45]), using different insert libraries considerably increases the cost per genome. On the contrary, 10x Genomics Chromium allows for assembly of a comparable or even more continuous genome using only a single library for a fraction of the cost (see below). Furthermore, as we show here, this library technology generates high-quality assemblies from as low as 25x coverage (see Eureka assembly), while the recommended coverage for PE plus MP assemblies is approximately 80x to 100x [46]. We do note, however, that the most recent wolf genome used a variety of PE and MP libraries to produce a highly continuous assembly with approximately 30x total coverage [36]. Recently, Mohr and colleagues [37] presented a highly continuous assembly of the endangered Hawaiian monk seal (∼2.4 Gb total genome assembly length) using a combination of 10x Genomics Chromium and Bionano Genomics optical mapping. Interestingly, their 10x Genomics Chromium (sans additional Bionano) assembly showed N50 statistics that are similar to those reported here (scaffold N50 22.23 Mb), showing that 10x Genomics Chromium technology alone consistently generates highly continuous mammalian genome assemblies.

A limitation of 10x Genomics Chromium technology is the requirement of fresh tissue samples for the isolation of HMW DNA. This can be difficult or impossible to obtain from some endangered species. Fortunately, small amounts of mammalian blood yield sufficient amounts of HMW DNA when properly stored. Additionally, DNA extraction kits such as the Qiagen MagAttract kit can extract sufficient amounts of HMW DNA from as little as 200 μL (see Supplementary Information S1 and Supplementary Fig. S1). For museum samples or tissues stored for extended periods of time, reference-based mapping might be the only option to extract long-range genomic information. However, for extant endangered species, especially those with individuals in captivity, 10x Genomics Chromium offers a cost-effective approach to sequence genomes. For species with genome sizes <1 Gb and between ∼3 Gb and 5.8 Gb, special data processing will need to be applied (see [47]). In addition, the amplification primers for the 10x Chromium library preparation are designed for GC contents similar to human (∼41%), implying that the method might not work as well for genomes that strongly divert from this GC content (e.g., for some invertebrates).

### Cost-effectiveness

Sequencing costs are steadily dropping. At the time the sequencing for this project was carried out, a lane on the Illumina HiSeqX cost (output of ∼120 Gb) approximately $1,500–$2,000 and a 10x Genomics library prep ranged from $450 to $1,000, thus allowing the generation of high-quality *de novo* genomes for less than $3,000 total (2016–2017). As we have shown, the 10x method only requires a single library to be sequenced to an average coverage of 25x–75x for comparable results. Furthermore, computational resources required to assemble the genome are very low.  The current version of Supernova 1.2 requires a minimum of 16 central processing unit (CPU) cores and 244 Gb of memory (for a human genome at 56x coverage; [48]), and the assembly can be carried out in only a few days (depending on the number of available CPU cores). This is a reduction of about five times the memory requirement compared to the first version of Supernova. Additionally, Supernova does not require parameter input or tuning, thus allowing even novices to easily assemble 10x Genomics Chromium-based genomes.

For a comparable Illumina assembly, such as the one produced in Gopalakrishnan et al. (2017), the cost would include two PE and two MP libraries plus the sequencing costs [36]. Although PE libraries are relatively cheap to produce ($120–$180 USD), MP libraries can be much more expensive depending on their input size ($2,000–$3,000 for larger insert sizes, or $700–$1,000 if non-size selected). In addition, MP libraries require a much larger quantity of starting material compared to the 10x library prep.

### Applications in conservation

Traditionally, conservation biologists have obtained a great deal of genetic information from a few microsatellite markers and/or nuclear and mitochondrial loci. The analysis of microsatellite markers can provide a snapshot into contemporary population structure, but this method risks providing incomplete information on selection and migration and can be an unreliable way to identify individuals from degraded low-quality DNA samples (such as scat) due to the stochastic behavior of marker amplification (allelic dropout; [49–51]). Moreover, microsatellites can be difficult to successfully design and develop, which can quickly increase costs for species that have little to no genetic information available. The ability to rapidly and cost-effectively generate full genomes will allow conservation biologists to bridge this gap and harvest crucial fine-scale population information for population parameters such as inbreeding (e.g., [52]), load of deleterious mutations (e.g., [53]), gene flow (e.g., [54]), and population structure (e.g., [55]). Once a reference genome has been assembled, optional (low-coverage) resequencing data from several individuals allow for the typing of genome-wide information such as SNPs, potentially neutral microsatellite loci, and other genomic regions of interest. These data can then be used to investigate the aforementioned population parameters and yield additional insights into adaptive genetic variation and perhaps the adaptive potential of different populations or species.

### Heterozygosity within African wild dog individuals

A high number of heterozygous sites were shared between all three individuals in this study, with sister 1 and sister 2 sharing more heterozygous sites than either shared with Eureka. Each of the individuals further showed a high number of singletons (heterozygous sites only found in one individual). Even when compared to the two low-coverage genomes from Campana et al., we find a high number of shared sites [21]. As expected, we see a much higher rate of singletons in these two individuals. Due to the low coverage (5.7x–5.8x average coverage), we suspect a higher proportion of the called heterozygous sites to be false positives due to sequencing errors, which could potentially be removed with more stringent filtering. Heterozygosity per site estimates indicate a high within-individual diversity. Estimates ranged from 0.0007 to 0.001 for Eureka and from 0.0009 to 0.0012 for sister 2, which are similar to those obtained for lions (0.00074–0.00148) and tigers (0.00087–0.00104) [56]. Intriguingly, other threatened carnivores, such as the Iberian lynx (*Lynx pardinus*), the cheetah (*Acinonyx jubatus*), and the island fox (*Urocyon littoralis*), show nearly 10-fold lower heterozygosity (0.0001 [55], 0.0002 [39], and 0.000014–0.0004 [40], respectively). The high within-individual heterozygosity could be a result of their social structure, as only unrelated individuals come together to form new packs through dispersal. In addition, Hwange National Park is considered to be a part of the most continuous population of African wild dogs, which may explain the high heterozygosity of sister 1 and sister 2 [19]. Further sequencing of other populations and additional unrelated individuals will be needed to determine whether the high within-individual heterozygosity is a range-wide phenomenon in African wild dogs.

The Supernova software reports distance between heterozygous site estimates (see Supplementary Table S1). Interestingly, those estimates were much lower than the ones obtained based on the genotype calls produced with ANGSD. While Supernova estimated this distance to be 2.6 kb in sister 1, 3.1 kb in sister 2, and 7.1 kb in Eureka, the ANGSD based estimates range from 850 bp to 1.2 kb for sister 1, 814 bp to 1.1 kb for sister 2, and 999 bp to 1.5 kb for Eureka, depending on the posterior cutoff used. Supernova calculates the distance between heterozygous sites as part of the assembly process. However, when the fasta consensus sequence is called, part of the variation can get flattened (see, e.g., [32]). This phenomenon is typically seen in regions between megabubbles, which are nominally homozygous, but could in fact have some variation that cannot be phased by Supernova. We also note that heterozygosity values obtained using genotype calls in ANGSD could be biased, as they are based on the nominal and not the effective coverage. The nominal coverage is the total number of reads that cover a site in the assembly, whereas for the effective coverage, only reads from different bar codes are included in the estimation. If individual bar coded regions amplified with different efficiency during the library preparation step, then heterozygosity estimates could be unreliable. However, this should not strongly affect genome-wide heterozygosity estimates, as we expect this issue to be rare.

## Potential Implications

We find that the 10x Genomics Chromium system can be used to assemble highly continuous and accurate mammalian genome assemblies for less than $3,000 per genome (sequenced 2016 and 2017). The method can be easily applied to species of conservation concern for which genomic methods could greatly benefit their management and monitoring programs. For the African wild dog, these genomes will facilitate more reliable and cost-effective conservation efforts through the use of resequencing and SNP-typing methods. Compared to other species of conservation concern, the African wild dog has a relatively high heterozygosity. Using demographic analyses, we also demonstrate that these wild dog populations appear to have been stable at lower effective population sizes for the past 100,000 years. Additional studies should inquire whether this is consistent for populations across the African continent and evaluate current effective population sizes. More studies are also required to understand how both the social biology and recent precipitous population declines have impacted the population genomic structure of African wild dogs and how management might use this information for the benefit and longevity of the species.

### Methods

#### Samples

Note that detailed methods can be found in the Supporting Information. Blood samples from two individuals belonging to the same pack in Hwange National Park, Zimbabwe, were provided by Painted Dog Conservation (CITES Export permit: ZW/0842/2015, ESA import permit: MA66259B-0, Research Council of Zimbabwe permit: 0 2553). These individuals were presumed to be sisters from direct observation of their litter at the den (here, named sister 1 and sister 2). DNA was extracted from samples two weeks after storage at –80°C. The third sample was provided by the Endangered Wolf Center, Eureka, Missouri, from a captive-born individual (here, named Eureka). DNA was extracted nine days after the sample was taken (additional information on sample storage can be found in Appendix S1). Though the Chromium library preparation does not require large amounts of DNA, the DNA should have a mean molecule length >200 kb (HMW). DNA from all individuals was extracted from blood samples using the QIAGEN MagAttract HMW DNA kit following the provided instructions.

#### Genome assembly

We constructed one sequencing library per individual using the 10x Genomics Chromium System with 1.2 ng of HMW input DNA. All libraries were then sequenced on the Illumina HiSeqX (sister 2, Eureka) or HiSeq 4000 (sister 1) platform. We subsequently assembled the three genomes using the 10x Genomics genome assembler Supernova 1.1.1 [32, 57]) using default assembly parameters.

#### Assembly quality assessment

We used the Supernova assembler as well as scripts from Assemblathon 2 to determine continuity statistics, such as the scaffold N50 and the total number of scaffolds [58]. We further applied the program BUSCO v2 (BUSCO, RRID:SCR_015008) [59] to assess the presence of nearly universal lineage-specific single-copy orthologous genes in our assemblies using the mammalian gene set from OrthoDB v9 (OrthoDB, RRID:SCR_011980; 4104 genes; available at [60]). We compare these results to the high-quality canFam3.1 assembly of the domestic dog ([33]; *Canis familiaris*). The canFam3.1 assembly was built on 7x coverage of Sanger reads and Bacteria Artificial Chromosome (BAC)library sequencing and has a scaffold N50 of 46 Mb. We also inferred the number of BUSCOs in the recently published Hawaiian monk seal genome (which was assembled using a combination of 10x Genomics Chromium and Bionano Genomics Irys data) and the two previously published African wild dog genomes (sequenced with basic short-read Illumina technology at low coverage and assembled using the domestic dog for reference mapping; [21]).

#### Repeat identification and masking

Next, we identified repetitive regions in the genomes as another comparative measure of assembly quality and to prepare the genome for annotation. Repeat annotation was carried out using both homology-based and *ab initio* prediction approaches. We used the canid RepBase [61, 62] repeat database for the homology-based annotation within RepeatMasker (RepeatMasker, RRID:SCR_012954) [63]. We then carried out *ab initio* repeat finding using RepeatModeler (RepeatModeler, RRID:SCR_015027).

#### Gene annotation

Gene annotation for the three assemblies was performed with the genome annotation pipeline Maker3 (MAKER, RRID:SCR_005309) [64], which implements both *ab initio* prediction and homology-based gene annotation by leveraging previously published protein sequences from dog, mouse, and human.

Orthologous genes between the three African wild dog assemblies, as well as paralogous genes within each individual, were inferred using Proteinortho [65]. Proteinortho applies highly parallelized reciprocal blast searches to establish orthology and paralogy for genes within and between gene annotation files.

#### Variant rates

In order to estimate within-individual heterozygosity, we output a single pseudohaplotype using the “style = pseudohap” parameter within Supernova from sister 2 to represent the reference sequence. Next, we mapped the raw reads from all three individuals to the reference using BWA-MEM [56]. We then converted the resulting SAM files to BAM format using Samtools [58] and sorted and indexed them using Picard (Picard, RRID:SCR_006525; [66]). Realignment around insertion/deletion regions and duplicate marking were performed using GATK (GATK, RRID:SCR_001876). Finally, we called heterozygous sites using a probabilistic framework implemented in ANGSD [67, 68, 69]. We tested different posterior probability cutoffs (1, 0.999,0.99, 0.98, and 0.95). To allow for comparison between all individuals, we downsampled our three assemblies to 20x mean nominal coverage (total number of reads covering a position, independent of their bar code) for our analyses. Heterozygosity was then simply calculated as the ratio of variable sites to the total number of sites (variable and invariable). Supernova also outputs the distance between heterozygous sites as part of their assembly report. We then used the read data of Campana et al. [20] and mapped them to our sister 2 assembly to compare heterozygosity estimates (using the approach outlined above). Next, we estimated the number of shared heterozygous sites between our individuals and between our individuals and the individuals from Campana et al. [20]. To do so, we used the *gplots* library in R [70] to calculate the overlap between the three sets and to display them in a Venn diagram.

Different pseudohaplotypes were obtained through the Supernova software by selecting either the “–style = pseudohap” or “–style = pseudohap2.” The two fasta files produced by “pseudohap2” were then analyzed as described above.

#### Demographic history

We filtered each genome for putative X chromosome sequences by first aligning them to the domestic dog X scaffold [33]. Scaffolds showing significant alignment were then further filtered using the program Basic Local Alignment Search Tool [71]. The top hit for each alignment was chosen, and all scaffolds that aligned with the mouse, human, pig, domestic dog, or domestic cat X chromosome were removed. This was repeated for each assembly.

We then mapped the raw reads to the subset of scaffolds using BWA-MEM and called the consensus sequence using SAMtools and BCFtools (SAMtools/BCFtools, RRID:SCR_005227) [72, 73]. Population history was reconstructed using pairwise sequentially Markovian coalescent and scaled using a mutations/site/generation rate of 6.0 × 10^−9^ and a generation time of five years [41]. This generation time a mutation/site/generation rate was chosen because it was the average mutation/site/generation rate inferred in Campana et al. [21].

## Availability of supporting data

Genomic and read data are available in the National Center for Biotechnology Information database under project accession PRJNA488046. Additional supporting data can be found in the *GigaScience* repository, GigaDB [38].

## Supporting Information

Detailed information on methods, Supernova output, repeat annotation, gene annotation, heterozygosity calculations, and different posterior probability cutoffs are available online. The authors are solely responsible for the content and functionality of these materials. Queries (other than absence of the material) should be directed to the corresponding author.

## Additional files

Supporting_information_AWD_Gigascience_final_update.docx

## Abbreviations

BUSCO: Benchmarking Universal Single-Copy Orthologs; CPU: central processing unit; HMW: high-molecular-weight; MP: mate-pair; Mya: million years ago; PE: paired-end; SNP: single-nucleotide polymorphism.

## Competing interests

J.S.is a board member of 10x Genomics Inc. R.W.T. is founder of End2End Genomics Inc. The remaining authors declare that they have no competing interests.

## Funding

This work was funded by a donation to the Program for Conservation Genomics at Stanford University.

## Author contributions

J.S., C.S.Z., P.B., S.P., E.A., and D.P. conceived the project. E.M., H.M., O.M., and R.M.C. contributed samples and insight to the project. R.T. assembled the genomes. E.A. and S.P. performed the genome annotation and downstream analyses. E.A., S.P., C.S.T., D.P., and R.T. wrote the paper. All authors read and approved the final manuscript.

## Supplementary Material

GIGA-D-17-00324_Original_Submission.pdf

GIGA-D-17-00324_Revision_1.pdf

Response_to_Reviewer_Comments_Original_Submission.pdf

Reviewer_1_Report_(Original_Submission) -- Andreas Chavez 1/31/2018 Reviewed

Reviewer_2_Report_(Original_Submission) -- M. Thomas P. Gilbert 2/5/2018 Reviewed

Reviewer_3_Report_(Original_Submission) -- Klaus-Peter Koepfli, PhD 2/6/2018 Reviewed

Reviewer_3_Report_(Revision_1) -- Klaus-Peter Koepfli, PhD 6/6/2018 Reviewed

Supplemental File
