# Supplementary material for: Cost-effective assembly of the African wild dog (Lycaon pictus) genome using linked reads
Source: Gigascience. 2018 Oct 22;8(2):giy124. doi: 10.1093/gigascience/giy124 (PMC6350039; doi:10.1093/gigascience/giy124)
Supplement: GIGA-D-17-00324_Original_Submission.pdf [file giy124_giga-d-17-00324_original_submission.pdf]

# GigaScience


# Entering the era of conservation genomics: Cost-effective assembly of the African wild dog genome using linked reads.

--Manuscript Draft--

| | |
|---|---|
| **Manuscript Number:** | GIGA-D-17-00324 |
| **Full Title:** | Entering the era of conservation genomics: Cost-effective assembly of the African wild dog genome using linked reads. |
| **Article Type:** | Research |
| **Funding Information:** | John Stuelpnagel — Not applicable |
| **Abstract:** | A high-quality reference genome assembly is a valuable tool for the study of non-model organisms across disciplines. Genomic techniques can provide important insights about past population sizes, local adaptation, and even aid in the development of breeding management plans. This information can be particularly important for fields like conservation genetics, where endangered species require critical and immediate attention. However, funding for genomic-based methods can be sparse for conservation projects, as costs for general species management can consume budgets. Here we report the generation of high-quality reference genomes for the African wild dog (Lycaon pictus) at a low cost, thereby facilitating future studies of this endangered canid. We generated assemblies for three individuals from whole blood samples using the linked-read 10x Genomics Chromium system. The most continuous assembly had a scaffold N50 of 21 Mb, a contig N50 of 83 Kb, and completely reconstructed 95% of conserved mammalian genes as reported by BUSCO v2, indicating a high assembly quality. Thus, we show that 10x Genomics Chromium data can be used to effectively generate high-quality genomes of mammal species from Illumina short-read data of intermediate coverage (~25-50x). Interestingly, the wild dog shows a much higher heterozygosity than other species of conservation concern, possibly as a result of its behavioral ecology. The availability of reference genomes for non-model organisms will facilitate better genetic monitoring of threatened species such as the African wild dog and help researchers and conservationists to better understand the ecology and adaptability of those species in a changing environment. |
| **Corresponding Author:** | Ellie Armstrong<br>Stanford University<br>UNITED STATES |
| **Corresponding Author Secondary Information:** | |
| **Corresponding Author's Institution:** | Stanford University |
| **Corresponding Author's Secondary Institution:** | |
| **First Author:** | Ellie Armstrong |
| **First Author Secondary Information:** | |
| **Order of Authors:** | Ellie Armstrong |
| | Ryan W Taylor |
| | Stefan Prost |
| | Peter Blinston |
| | Esther van der Meer |
| | Hillary Madzikanda |
| | Olivia Mufute |
| | Roseline Madisodza-Chikerema |
| | John Stuelpnagel |


Powered by Editorial Manager® and ProduXion Manager® from Aries Systems Corporation

| | |
|---|---|
| | Claudio Sillero-Zubiri |
| | Dmitri Petrov |
| **Order of Authors Secondary Information:** | |
| **Opposed Reviewers:** | |
| **Additional Information:** | |
| **Question** | **Response** |
| Are you submitting this manuscript to a special series or article collection? | No |
| **Experimental design and statistics**<br><br>Full details of the experimental design and statistical methods used should be given in the Methods section, as detailed in our Minimum Standards Reporting Checklist. Information essential to interpreting the data presented should be made available in the figure legends.<br><br>Have you included all the information requested in your manuscript? | Yes |
| **Resources**<br><br>A description of all resources used, including antibodies, cell lines, animals and software tools, with enough information to allow them to be uniquely identified, should be included in the Methods section. Authors are strongly encouraged to cite Research Resource Identifiers (RRIDs) for antibodies, model organisms and tools, where possible.<br><br>Have you included the information requested as detailed in our Minimum Standards Reporting Checklist? | Yes |
| **Availability of data and materials**<br><br>All datasets and code on which the conclusions of the paper rely must be either included in your submission or deposited in publicly available repositories (where available and ethically appropriate), referencing such data using a unique identifier in the references and in the "Availability of Data and Materials" section of your manuscript.<br><br>Have you have met the above requirement as detailed in our Minimum | Yes |

Powered by Editorial Manager® and ProduXion Manager® from Aries Systems Corporation

| | |
|---|---|
| Standards Reporting Checklist? | |

Powered by Editorial Manager® and ProduXion Manager® from Aries Systems Corporation

Manuscript

Click here to download Manuscript AWD_gigascience_main_final.docx

**Entering the era of conservation genomics: Cost-effective assembly of the African wild dog genome using linked reads.**

Ellie E. Armstrong[1]*, Ryan W. Taylor[1]*, Stefan Prost[1,2], Peter Blinston[3], Esther van der Meer[3], Hillary Madzikanda[3], Olivia Mufute[4], Roseline Mandisodza-Chikerema[4], John Stuelpnagel[5], Claudio Sillero-Zubiri[6], Dmitri Petrov[1]

[1] Program for Conservation Genomics, Department of Biology, Stanford University, Stanford, CA, USA

[2] Department of Integrative Biology, University of California, Berkeley, CA, USA

[3] Painted Dog Conservation, Dete, Zimbabwe

[4] The Zimbabwe Parks & Wildlife Management Authority, Zimbabwe

[5] 10x Genomics, Inc., Pleasanton, CA

[6] Wildlife Conservation Research Unit, Zoology, University of Oxford, The Recanati-Kaplan Centre, Tubney, UK

* These authors contributed equally to this work.

Corresponding Author: Ellie E. Armstrong (elliea@stanford.edu)

## Abstract

***Background***

A high-quality reference genome assembly is a valuable tool for the study of non-model organisms across disciplines. Genomic techniques can provide important insights about past population sizes, local adaptation, and even aid in the development of breeding management plans. This information can be particularly important for fields like conservation genetics, where endangered species require

critical and immediate attention. However, funding for genomic-based methods can be sparse for conservation projects, as costs for general species management can consume budgets.

***Findings***

Here we report the generation of high-quality reference genomes for the African wild dog (*Lycaon pictus*) at a low cost, thereby facilitating future studies of this endangered canid. We generated assemblies for three individuals from whole blood samples using the linked-read 10x Genomics Chromium system. The most continuous assembly had a scaffold N50 of 21 Mb, a contig N50 of 83 Kb, and completely reconstructed 95% of conserved mammalian genes as reported by BUSCO v2, indicating a high assembly quality.

***Conclusions***

We show that 10x Genomics Chromium data can be used to effectively generate high-quality genomes of mammal species from Illumina short-read data of intermediate coverage (~25-50x). Interestingly, the wild dog shows a much higher heterozygosity than other species of conservation concern, possibly as a result of its behavioral ecology. The availability of reference genomes for non-model organisms will facilitate better genetic monitoring of threatened species such as the African wild dog and help researchers and conservationists to better understand the ecology and adaptability of those species in a changing environment.

**Keywords**

Conservation genomics, 10x Genomics Chromium, African wild dog, *Lycaon pictus*, *de novo* Assembly

## Background

Major population declines have been observed in vertebrate groups over the past several hundred years, primarily due to anthropogenic change [1]. This decline has resulted in extinction rates unprecedented in recent history [1, 2]. The conservation of extant species will require major efforts in restoring and preserving habitat, along with protection, management, and investment by local stakeholders. Though, by definition, all species of conservation concern exist as small populations, populations can still retain genetic variation that was generated and maintained a few generations back, when population sizes were much larger. Within patterns of historic genetic variation are signals of demographic history, gene flow, and natural selection which can inform efforts towards the long-term survival of species. In addition to signals of a species history, genetic information can be used to uncover important contemporary or very recent events and processes. For example, Epstein, Jones [3] identified genes that may confer facial tumor resistance in Tasmanian devils, suggesting that the ability to artificially select for resistance in non-infected populations may allow for a more robust population rescue and recovery. Genetic markers can be used to track individual movement across landscapes either indirectly by measuring relatedness, or directly by genotyping scat or hair left by an individual as it moves. Additionally, the identification and assignment of individuals through genotyping can be an important tool for law enforcement to assign contraband and confiscated materials to their geographic origin. Conservationists can also use fine grained measurements of reproductive success along with genotypes and environmental variables to gather a detailed understanding of the factors contributing to or limiting population growth, such as inbreeding depression. Taken together genomic tools are poised to have a major contribution to conservation [4, 5].

The African wild dog (*Lycaon pictus*) is a medium-sized (18-34kg), endangered carnivore that lives in scattered populations in sub Saharan Africa (Fig. 1A). The species is the only surviving member of a lineage of wolf-like canids [6].

Wild dogs have been subject to intense recovery efforts across their range [7, 8], but their global population is decreasing. It is estimated that only 6,600 adult wild dogs remain in 39 subpopulations [9]. The primary reasons for the species' population decline include habitat loss and fragmentation, as well as anthropogenic mortality (e.g. snaring, persecution, road kills, exposure to infectious diseases from domestic dogs) when they range beyond the borders of protected areas [7, 8, 10]. Due to their large ranges and low population densities, African wild dogs are more susceptible to these threats than most other carnivore species [8]. In addition, their complex social system and susceptibility to Allee effects appears to increase the species extinction risk [11, 12]. The dogs are obligate cooperative breeders which form packs consisting of an alpha male and female, their adult siblings, and pups and subadults from the dominant pair [13]. Subadults that have reached reproductive age disperse in single sex groups and form new packs by joining dispersing groups from the opposite sex [14]. Pack members rely on each other for hunting, breeding, and defense against natural enemies and pack size has been found to be significant for hunting and breeding success [13, 15, 16]. When pack size becomes critically low, e.g. due to anthropogenic mortality, this dependence on helpers increases the risk of pack extinction and reduces the number of successful dispersals (Courchamp, Clutton-Brock [12], but see Creel and Creel [17]).

Prior genetic studies on wild dogs using a combination of mitochondrial, microsatellite, and MHC markers have resulted in varying estimates of the start of the species decline on the African continent [18, 19]. Consistent with expectation, the data shows strong structuring between populations due to habitat fragmentation and isolation, as well as low genetic diversity within populations [19, 20]. For species that are experiencing such rapid and alarming declines, estimates that are particularly important for management decisions, such as local adaptation, effective population size, and inbreeding are greatly improved by the use of whole-genome methods. Recently, Campana and colleagues [21] sequenced low-coverage genomes of two

African wild dog individuals from Kenya and South Africa, respectively, to investigate demographic history and signatures of selection of these two separate populations. By mapping these data to the domestic dog genome, they discovered approximately 780,000 single nucleotide polymorphisms (SNPs) between their two individuals which could be used to develop SNP typing for the two populations. However, given the low coverage of their genomes (5.7-5.8x average coverage) and the small number of individuals, additional sequencing will be needed to verify the authenticity of those SNPs. Further, important structural variation can be overlooked when mapping against a reference genome from a different genus, and mapping can be hindered if the divergence is high between the sample and the reference (see e.g. Shapiro and Hofreiter [22]). The groups containing the African wild dog and the domestic dog are estimated to have split approximately 7.5-10 Mya and furthermore, the domestic dog has undergone significant genomic selection in recent time [23].

Despite the ever-declining cost to sequence DNA, the routine use of genomic approaches in conservation is still far from a reality. One of the major remaining barriers is the lack of reference genomes for species of conservation concern. Generating a *de novo* reference genome requires the sequencing and assembly of the 100s of millions to billions of base-pairs that make up a genome. The first mammalian genome (human) required a massive collaboration between hundreds of scientists and nearly $3 billion US dollars (1990-2001; [24, 25]). Fortunately, the cost to sequence DNA is now low enough that every base-pair in a typical mammalian genome can be sequenced to high coverage for a few thousand US dollars. However, these low cost sequencing methods produce very short sequences of 150-300 base-pairs in length (for a review on sequencing methods see Goodwin, McPherson [26]). Because large proportions of typical mammal genomes consist of repetitive sequences, it has been impossible to assemble highly-contiguous genomes from only these short sequences. In order to achieve higher continuity, more elaborate and expensive library preparation or alternative sequencing

technologies have to be used [26, 27]. Among others, these include mate-pair libraries, chromatin folding based libraries, such as cHiCago [28] or HiC [29], and long-read sequencing technologies, such as Pacific Biosciences and Oxford Nanopore Technology. While the resulting genomes can show high continuity, those methods substantially increase the costs of sequencing projects and thus can hinder the generation of genomes for conservation biology purposes.

Here we report the use of the Chromium system developed by 10x Genomics [30], a genomic library preparation technique that facilitates cost-effective (around $2,500) assemblies using short sequencing reads, to assemble three African wild dog genomes. In brief, the 10x Genomics Chromium system is based on dilution of high molecular weight (HMW) DNA. It uses as little as 1ng of input DNA, which is well-suited for a variety of applications. During library preparation, gel beads, so-called GEMs, are mixed with DNA and polymerase for whole-genome amplification. Each gel bead has primer oligos (44nt long) attached to its surface. These contain a priming site (22nt partial R1), a 16nt barcode region, and a 6nt N-mer region that binds to different places on the original DNA fragment. The low amount of input DNA ensures that each gel bead only binds a single (up to ~100kb) DNA fragment. In the next step, amplification of short reads along the original DNA fragment is performed within each gel bead. In most cases, this amplification results in spotted read coverage along the fragment. However, all reads from a respective GEM contain identical barcodes and can later be assigned to groups originating from the same DNA molecule. The information about which molecule of DNA the sequence originated from greatly increases the ability to identify the location of repetitive sequences. The library is then sequenced on an Illumina platform and the raw read data is assembled by the 10x Genomics Supernova assembler. This assembler is very user-friendly and does not require any prior knowledge about input parameters for the assembly.

We *de novo* assembled three African wild dog genomes using the 10x Genomics Chromium platform in order to investigate whether this technology is suitable for conservation genomic purposes. For any endangered species, a genome can have large conservation impacts, but high-quality genomes have historically been costly or impossible due to the sampling requirements and in addition, downstream analyses can be challenging. Thus, in order for it to be useful for conservation purposes the technology needs to be (a) cost-effective and (b) user-friendly. Furthermore, we test the 10x Genomics Chromium based assemblies for reproducibility, continuity, conserved gene completeness, and repetitive content, as compared to the previously published domestic dog genome.

**Data Description & Analyses**

*Assembly of the African wild dog genome*

Using 10x Genomics Chromium technology, we generated DNA libraries for three African wild dog individuals, two of which were collected from a wild pack in the Hwange National Park, Zimbabwe and are presumed to be sisters (named Sister 1 and Sister 2), and a third unrelated individual from the Endangered Wolf Center, Eureka, Missouri (named Eureka). A summary of the assembly statistics output by the Supernova assembler can be found in Table 1 (detailed statistics for each genome assembly can be found in Supporting Information Table 1). We generated 1,200 million paired-end reads for Sister 1, 801.56 million reads for Sister 2, and 427.6 million reads for Eureka. We then used the reads to assemble each genome using the 10x Genomics Supernova assembler (as explained in https://support.10xgenomics.com/de-novo-assembly/software/overview/welcome). The mean input DNA molecule length reported by the Supernova assembler for Sister 1 was 19.91kb, Sister 2 was 77.03kb, and Eureka was 52.00kb. All three assemblies corroborate a genome size of approximately 2.3Gb, which is similar to

that of the domestic dog (2.4Gb). These three assemblies together constitute the first reported *de novo* assemblies for the African wild dog species.

We then calculated the scaffold and contig N50 statistics, which are indicative of assembly continuity. The Sister 1 assembly resulted in a contig and scaffold N50 of 61.34 kb and 7.91 Mb, respectively, the Sister 2 assembly achieved 83.47 kb contig and 21.34 Mb scaffold N50s, and finally the Eureka assembly had 50.15 kb contig and 15.31 Mb scaffold N50s (Table 1). While our contig and scaffold N50's are smaller than the ones from the most recent dog genome (267kb and 45.9Mb, respectively), they are still larger than most mammalian genomes assembled that used only short read data (see e.g. Lok, Paton [31]).

*Conserved Genes*

The program BUSCO (Benchmarking Universal Copy Orthologs) uses highly conserved single copy orthologous genes from a number of different taxa and groups in order to test assemblies (both genomic and transcriptomic) for gene completeness, fragmentation, or absence as an indicator of assembly quality. Using BUSCO v2 on our assemblies, we found that the most continuous assembly, Sister 2, completely recovered 95.1% of conserved genes (Mammalia gene set; Table 2). Sister 1 and Eureka recovered 95.4% and 93.3% of complete conserved genes, respectively. Using the same analysis, we found 95.3% of complete conserved genes in the latest dog assembly (canFam3.1). This indicates that although the domestic dog assembly is more continuous overall, our assemblies recover nearly the same or even higher number of conserved genes. Surprisingly, Sister 1 had the least number of missing genes out of all the assemblies assessed, despite lower continuity than Sister 2. We also ran BUSCO on the Hawaiian monk seal genome, generated through the combination of 10x Genomics Chromium and Bionano Genomics Irys data, and found it recovered 94.6% of conserved genes using BUSCO. This suggests that using Bionano in addition to 10x does not greatly

improve the reconstruction of the gene regions. However, the Hawaiian monk seal genome has a scaffold N50 of approximately 28Mb, so Bionano may improve the overall assembly continuity compared to 10x Genomics alone. The low coverage genomes from Campana et al. 2016 achieved a BUSCO score of 92.8% for the individual from Kenya and 94.8% for the individual from South Africa. These scores are similar to those from the dog assembly, which was the reference the reads were mapped to initially.

*Repeat annotation*

We identified repetitive regions of the genome in order to discern how well these complex areas were assembled by the 10x Genomics Chromium technology. Using both RepeatMasker and RepeatModeler, we found that for all three wild dog assemblies, total repeat content was evaluated to be within 3% of one another, which indicates consistency among assemblies from a single species (Supporting Information Table S2). No single repeat category was disproportionately affected during repeat annotation of the three genomes, which suggests that assembly quality was likely the most influential factor. Furthermore, repeat content of all wild dog assemblies was qualitatively similar to canFam3.1. As repetitive regions tend to be the most difficult regions to assemble, the similarity in repeat content between the African wild dog compared to that of the domestic dog, highlights the value of using 10x Genomics Chromium technology to produce accurate and continuous assemblies.

*Gene annotation*

The genome annotation pipeline Maker3 resulted in very similar numbers of annotated genes between all three African wild dog individuals and the domestic dog. Annotations ranged from 20,649 (Sister 2) to 20,946 (Sister 1) genes (Supporting Information Table S3). Using proteinortho to detect orthologous genes

between individuals and paralogous genes within individuals, we found 12,617 one:one orthologs present in all three individuals and 6,462 one:one orthologs in two out of the three individuals. We found 268 multi copy genes present in all three individuals and 37 not present in one individual. Overall, the number of annotated genes was comparable to those found in the dog genome (Supporting Information Table S3).

*Variant rates*

We found a high number of heterozygous sites to be shared between all three individuals (321k; here we report the heterozygous sites called using a posterior probability cutoff of 0.99; Fig. 1B). As expected, Sister 1 and Sister 2 share more heterozygous sites (344k) than either sister with Eureka (168k and 170k, for Sister 1 and Sister 2, respectively). Each individual shows a high number of singletons (heterozygous sites only found in one individual), with Sister 2 showing the highest number (1,100k), followed by Sister 1 (968k) and Eureka (825k). Even if we include the two low coverage genomes from Campana, Parker [21], we find a high number of shared heterozygous sites between all individuals (134k; Supporting Information Figure S1). As expected, we see a higher number of singletons in these two individuals, due to the lower reliability of the genotype calls caused by the low coverage (false positives caused by sequencing errors). We estimated a per site heterozygosity of 0.0008 to 0.0012 for Sister 1, 0.0009 to 0.0012 for Sister 2, and 0.0007 to 0.001 for Eureka using posterior cutoffs for genotype calls from 0.95 to 1 in ANGSD (Supporting Information, Fig. S1C). As can be seen in Figure 1C, except for a posterior probability cutoff of 1, where Sister 1 shows the highest heterozygosity, Sister 2 always shows the highest, Sister 1 the second highest and Eureka the lowest heterozygosity. Interestingly, Eureka shows a lower heterozygosity than the other two assemblies, even though its parents originated from South Africa and

Botswana. Our estimates show that, while being heavily threatened, African Wild dogs seem to still retain a relatively high within individual heterozygosity. We did not see any major difference between heterozygosity estimates from repeat-masked and unmasked genomes (data not shown). The Supernova software estimated a heterozygous position every 2.6kb, 3.1kb, and 7.14kb for Sister 1, Sister2, and Eureka, respectively (Supporting Information Table S4). On the contrary, estimates based on genotype calls using ANGSD showed much more frequent heterozygous positions (850bp - 1.2kb, 814bp - 1.1kb and 999bp - 1.5kb depending on the posterior cutoff used; Supporting Information Table S4).

## Discussion

### *Assembly continuity and quality*

All three African wild dog assemblies produced with 10x Genomics Chromium data showed high continuity, high recovery rates of conserved genes, and expected proportions of repetitive sequence; indicating that they are high-quality assemblies. The Sister 2 assembly, which has the highest mean molecule length, is also the most continuous (Contig N50: 83.47kb, Scaffold N50: 21.34Mb; Table 1). Interestingly, the Sister 1 genome has a higher contig N50 (61.34kb) than Eureka (50.15kb), but a lower scaffold N50 (7.91Mb and 15.31Mb, respectively). This may indicate that input molecule length is a key factor for scaffolding, while coverage is a key factor for contig assembly. Despite having the highest continuity of all three assemblies, Sister 2 did not show the highest BUSCO completeness scores (see Table 2), although the differences were minor and likely not meaningful as they could lie well within the uncertainty of the BUSCO analysis (with 95.1% complete BUSCOs compared to 95.4% for Sister 1). Sister 1 achieved the highest BUSCO scores, even compared to the latest domestic dog genome assembly (CanFam3.1; 95.2%), which has three times higher contig N50 and an almost six times higher scaffold N50. The high

scores are remarkable for the limited number of reads used for the assemblies (as low as 25x coverage). As expected, Sister 2, which showed the highest continuity also had the highest repeat content (see Supporting Information Table S2). However, all three assemblies resulted in similar repeat contents in terms of repeat composition as well as overall percentage (within 3% of each other), with the most continuous assembly (Sister 2) showing the highest number of repeats. Repeat composition in the African wild dog genomes was also similar to the domestic dog.

All assemblies yielded similar amounts of genes, with Sister 1 showing the highest number (see Supporting Information Table S3), which reflects its BUSCO scores. Closer investigations of one:one and one:many orthologs further showed a very good agreement between annotations obtained from all three individuals. The numbers of annotated genes for all three African wild dogs were similar to those calculated for the latest domestic dog assembly.

*10x Genomics Chromium system: Feasibility and caveats*

Most mammal genomes published in the last several years use a mixture of paired-end (PE) and multiple mate pair (MP) Illumina libraries (e.g. Lok, Paton [31] and Liu, Lorenzen [32]). While often resulting in good continuity (e.g. Liu, Lorenzen [32] or Huang, Zhao [33]), using different insert libraries considerably increases the cost per genome. On the contrary, 10x Genomics Chromium allows for assembly of a comparable or even more continuous genome using only a single library for a fraction of the cost (see below). Furthermore, as we show here, this library technology generates high-quality assemblies from as low as 25x coverage (see Eureka assembly), while the recommended coverage for PE plus MP assemblies is 100x [34]. Recently, Mohr and colleagues [35] presented a highly continuous assembly of the endangered Hawaiian Monk seal (~2.4Gb total genome assembly length) using a combination of 10x Genomics Chromium and Bionano Genomics

optical mapping. Interestingly, their 10x Genomics Chromium assembly showed similar N50 statistics to those reported here (scaffold N50 22.23Mb), showing that 10x Genomics Chromium technology alone enables the generation of high-quality mammalian genome assemblies.

A limitation of 10x Genomics Chromium technology is the requirement of fresh tissue samples for the isolation of HMW DNA. This can be difficult or impossible to obtain from some endangered species. Fortunately, small amounts of mammalian blood yield sufficient amounts of HMW DNA when properly stored. Additionally, DNA extraction kits such as the Qiagen MagAttract kit can extract sufficient amounts of HMW DNA from as little as 200µl. For museum samples, or tissues stored for extended periods of time, reference-based mapping might be the only option to extract long-range genomic information. However, for extant endangered species, especially those with individuals in captivity, 10x Genomics Chromium offers a cost-effective approach to sequence genomes. For species with genome sizes <1Gb and between ~3Gb and 5.8Gb special data processing will need to be applied (see https://support.10xgenomics.com/de-novo-assembly/sample-prep/doc/technical-note-supernova-guidance). In addition, the amplification primers for the 10x Chromium library preparation are designed for GC contents similar to human (~41%), implying that the method might not work as well for genomes that strongly divert from this GC content (e.g. for some invertebrates).

*Cost effectiveness*

Sequencing costs are steadily dropping. At the time the sequencing for this project was carried out a lane on the Illumina HiSeqX cost approximately $1,500 - $2,000 and a 10x Genomics library ranged from $450 to $1000, thus allowing the generation of high quality *de novo* genomes for less than $3,000 total (prices obtained from US sequencing facilities). Furthermore, prices are likely to decline as

technology improves. Even more so, independent of sequencing lane costs, this method only requires a single library to be sequenced to an average coverage of 25 - 75x, unlike other methods which require multiple libraries at higher coverage. As we have shown here, a continuous assembly can be generated from as little as 25x. Furthermore, computational resources required to assemble the genome are very low. The current version of Supernova 1.2 only requires a minimum of 16 CPU cores and 244Gb of memory (for a human genome at 56x coverage; https://www.10xgenomics.com/), and the assembly can be carried out in only few days (depending on the number of available CPU cores). This is about a reduction of five times the memory requirement compared to the first version of Supernova. Even more so, Supernova does not require parameter input or tuning, thus allowing even novices to easily assemble 10x Genomics Chromium based genomes.

*Applications in conservation*

Traditionally, conservation biologists have obtained a great deal of genetic information from a few microsatellite markers and/or nuclear and mitochondrial loci. The analysis of microsatellite markers can provide a snapshot into contemporary population structure, but this method risks providing incomplete information on selection and migration and can be an unreliable way to identify individuals from degraded low-quality DNA samples (such as scat) due to the stochastic behavior of marker amplification (allelic dropout; Frantzen, Silk [36]; Taberlet and Luikart [37] ; Morin, Luikart [38]). Moreover, microsatellites can be difficult to successfully design and develop, which can quickly increase costs for species that have little to no genetic information available. The ability to rapidly and cost-effectively generate full genomes will allow conservation biologists to bridge this gap and harvest crucial fine-scale population information for population parameters such as inbreeding (e.g. Vieira, Fumagalli [39]), load of deleterious mutations (e.g. Robinson, Ortega-Del

Vecchyo [40]), gene flow (e.g. Pazmiño, Maes [41]) and population structure (e.g. Hampton, Spencer [42]). Once a reference genome has been assembled, optional (low coverage) re-sequencing data from several individuals allows for the typing of genome-wide information such as single-nucleotide polymorphisms (SNPs), potentially neutral microsatellite loci, and other genomic regions of interest. These data can then be used to investigate the abovementioned population parameters, but also further yield insights into adaptive genetic variation and perhaps the adaptive potential of different populations or species.

*Heterozygosity within African wild dog individuals*

A high number of heterozygous sites were shared between all three individuals in this study, with Sister 1 and Sister 2 sharing more heterozygous sites than either with Eureka. Each of the individuals further shows a high number of singletons (heterozygous sites only found in one individual). Even when compared to the two low coverage genomes from Campana et al. (2016) we find a high number of shared sites. As expected, we see a much higher rate of singletons in these two individuals. Due to the low coverage (5.7 - 5.8x average coverage) we predict a higher proportion of the called heterozygous sites to be false positives due to sequencing errors. Heterozygosity per site estimates indicate a high within individual diversity. Estimates ranged from 0.0007 - 0.001 for Eureka to 0.0009 - 0.0012 for Sister 2, which are similar to those obtained for lions (0.00074 – 0.00148) and tigers (0.00087 – 0.00104) [45]. Intriguingly, other threatened large bodied carnivores, such as the Iberian lynx (*Lynx pardinus*), the cheetah (*Acinonyx jubatus*), and the island fox (*Urocyon littoralis*) show nearly 10 fold lower heterozygosity (0.0001 [43], 0.0002 [44] and 0.000014 - 0.0004 [40], respectively). The high within-individual heterozygosity could be a result of their social structure, as only unrelated individuals come together to form new packs through dispersal. However, Hwange National

Park is considered to be a part of the most continuous population of African wild dogs which may explain the high heterozygosity of Sister 1 and Sister 2 (Girman et al. 2001). Further sequencing of other populations will be needed to assess whether the high within-individual heterozygosity is a range-wide phenomenon in African wild dogs. If true, this could be very good news for the survival of these species if external pressures (such as hunting, habitat fragmentation, etc.) can be reduced.

The Supernova software reports distance between heterozygous site estimates (see Supporting Information Table S1). Interestingly, those estimates were much lower than the ones obtained based on the genotype calls produced with ANGSD. While Supernova estimated this distance to be 2.6kb in Sister 1, 3.1kb in Sister 2 and 7.1kb in Eureka, the ANGSD based estimates range from 850bp - 1.2kb for Sister 1, 814bp - 1.1kb for Sister 2 and 999bp - 1.5kb for Eureka, depending on the posterior cutoff used. Supernova calculates the distance between heterozygous sites as part of the assembly process, however, when the fasta consensus sequence is called part of the variation can get flattened (see Weisenfeld, Kumar [30]). This phenomenon is typically seen in regions between megabubbles, which are nominally homozygous, but could actually have some variation that cannot be phased by Supernova. We also note that heterozygosity values obtained using genotype calls in ANGSD could also be biased, as they are based on the nominal and not the effective coverage. The nominal coverage is the total number of reads that cover a site in the assembly, whereas for the effective coverage only reads from different barcodes are included in the estimation. If individual barcoded regions amplified with different efficiency during the library preparation step, then heterozygosity estimates could be unreliable. However, this should not strongly affect genome-wide heterozygosity estimates, as we expect this issue to be rare. Heterozygosity

## Potential Implications

We find that the 10x Genomics Chromium system can be used to assemble highly continuous and accurate mammalian genome assemblies for less than $3,000 US dollars per genome (sequenced 2016 and 2017). The method can be easily applied to species of conservation concern for which genomic methods could greatly benefit their management and monitoring programs. For the African wild dog, these genomes will facilitate more reliable and cost-effective conservation efforts through the use of re-sequencing and SNP-typing methods. Compared to other species of conservation concern, the African wild dog has a relatively high heterozygosity. More studies are required to understand how both the social biology and recent precipitous population declines have impacted the population genomic structure of African wild dogs, and how management might use this information for the benefit and longevity of the species.

## Methods

Detailed Methods can be found in Supporting Information.

### *Samples*

Blood samples from two individuals belonging to the same pack in Hwange National Park, Zimbabwe were provided by Painted Dog Conservation (CITES Export permit: ZW/0842/2015, ESA import permit: MA66259B-0, Research Council of Zimbabwe permit: 02553). These individuals were presumed to be sisters from direct observation of their litter at the den (here, named Sister 1 and Sister 2). DNA was extracted two weeks after storage at -80ºC. The third sample was provided by the Endangered Wolf Center, Eureka, Missouri from a captive born individual (here named Eureka). DNA was extracted 9 days after the sample was taken. Though the Chromium library preparation does not require large amounts of DNA, the DNA should have a mean molecule length > 200kb (high-molecular weight, or HMW). DNA

from all individuals was extracted from blood samples using the QIAGEN MagAttract HMW DNA kit following the provided instructions.

*Genome Assembly*

We constructed one sequencing library per individual using the 10x Genomics Chromium System with 1.2ng of HMW input DNA. All libraries were then sequenced on the Illumina HiSeqX (Sister 2, Eureka) or HiSeq 4000 (Sister 1) platform. We subsequently assembled the three genomes using the 10x Genomics genome assembler Supernova 1.1.1 Weisenfeld, Kumar [30]; http://support.10xgenomics.com/de-novo-assembly/software/overview/welcome) using default assembly parameters.

*Assembly Quality Assessment*

We used the Supernova assembler as well as QUASTv4.3 to determine continuity statistics, such as the scaffold N50 and the total number of scaffolds [46]. We further applied the program BUSCO v2 [47] to assess the presence of nearly universal lineage specific single-copy orthologous genes in our assemblies using the mammalian gene set from OrthoDB v9 (4104 genes; available at http://busco.ezlab.org). We compare these results to the high-quality canFam3.1 assembly of the domestic dog (Hoeppner, Lundquist [48]; *Canis familiaris*). The canFam3.1 assembly was built on 7x coverage of Sanger reads and BAC end sequencing and has a scaffold N50 of 46Mb. We also inferred the number of BUSCO's in the recently published Hawaiian monk seal genome (which was assembled using a combination of 10x Genomics Chromium and Bionano Genomics Irys data) and the two previously published African wild dog genomes (sequenced with basic short read Illumina technology at low coverage and assembled using the domestic dog; [21]).

*Repeat Identification and Masking*

We next identified repetitive regions in the genomes as another comparative measure of assembly quality and to prepare the genome for annotation. Repeat annotation was carried out using both homology-based and *ab-initio* prediction approaches. We used the canid RepBase (http://www.girinst.org/repbase/; [49]) repeat database for the homology-based annotation within RepeatMasker (http://www.repeatmasker.org). We then carried out *ab-initio* repeat finding using RepeatModeler (http://repeatmasker.org/RepeatModeler.html).

*Gene Annotation*

Gene annotation for the three assemblies was performed with the genome annotation pipeline Maker3 [50], which implements both *ab-initio* prediction and homology-based gene annotation by leveraging previously published protein sequences from dog, mouse, and human.

Orthologous genes between the three African wild dog assemblies, as well as paralogous genes within each individual, were inferred using proteinortho [51]. Proteinortho applies highly parallelized reciprocal blast searches to establish orthology and paralogy for genes within and between gene annotation files.

*Variant rates*

In order to estimate within individual heterozygosity, we selected a single pseudo-haplotype (in cases where genomic regions were phased into haplotypes, one of the two was chosen randomly) from Sister 2 to represent the reference sequence. Next we mapped the raw reads from all three individuals to the reference using bwa mem [52]. We then converted the resulting sam files to bam format using samtools [53], and sorted and indexed them using picard (http://broadinstitute.github.io/picard/). Realignment around insertion/deletion (indel) regions was performed using GATK, and finally, we called heterozygous sites using

a probabilistic framework implemented in ANGSD [54]. We tested different posterior probability cutoffs (1, 0.999,0.99 and 0.95). To allow for comparison between all individuals, we down-sampled all individuals to 20x mean nominal coverage (total number of reads covering a position, independent of their barcode) for our analyses. Heterozygosity was then simply calculated as the ratio of variable sites to the total number of sites (variable and invariable). Furthermore, Supernova outputs the distance between heterozygous sites as part of their assembly report. We further downloaded the read data of Campana, Parker [21] and mapped them against our Sister 2 assembly to compare heterozygosity estimates (using the approach outlined above). Next, we estimated the number of shared heterozygous sites between a) our individuals and b) our individuals and the two from Campana, Parker [21]. To do so, we used the *gplots* library in R (https://www.r-project.org) to calculate the overlap between the three sets and to display them in a Venn diagram.

## Availability of supporting data

The data sets supporting the results of this article will be uploaded to the GigaDB repository pending manuscript acceptance.

## Supporting Information

Detailed information on methods, Supernova output, repeat annotation, gene annotation, heterozygosity calculations, and different posterior probability cutoffs are available online. The authors are solely responsible for the content and functionality of these materials. Queries (other than absence of the material) should be directed to the corresponding author.

## Competing Interests

Author J. Stuelpnagel is a board member of 10x Genomics Inc. Author Ryan W. Taylor is founder of End2End Genomics Inc.

## Authors' contributions

Authors JS, CSZ, PB, SP, EA, and DP conceived the project. Authors EM, HM, OM, and RMC contributed samples and insight to the project. RT assembled the genomes. EA and SP performed the genome annotation and downstream analyses. EA, SP, CST, DP, and RT wrote the paper. All authors read and approved the final manuscript.

## Acknowledgements

We thank M. Agnew, C. Asa, L. Padilla, and W. Warren for assistance in obtaining the Eureka sample. T. Linderoth, T. Korneliussen, and K. Bi for help with the different heterozygosity calculations. D. Church from 10x Genomics for discussion on how SuperNova performs the heterozygous site calling. This work was funded by the a donation to the Program for Conservation Genomics at Stanford University.

## Literature Cited

1. Pimm, S.L., et al., *The biodiversity of species and their rates of extinction, distribution, and protection.* Science, 2014. **344**(6187): p. 1246752.
2. Ceballos, G., et al., *Accelerated modern human–induced species losses: Entering the sixth mass extinction.* Science Advances, 2015. **1**(5): p. e1400253.
3. Epstein, B., et al., *Rapid evolutionary response to a transmissible cancer in Tasmanian devils.* Nature communications, 2016. **7**: p. 12684.
4. Steiner, C.C., et al., *Conservation genomics of threatened animal species.* Annu. Rev. Anim. Biosci., 2013. **1**(1): p. 261-281.
5. Shafer, A.B., et al., *Genomics and the challenging translation into conservation practice.* Trends in Ecology & Evolution, 2015. **30**(2): p. 78-87.

6. Girman, D., et al., *Molecular genetic and morphological analyses of the African wild dog (Lycaon pictus).* Journal of heredity, 1993. **84**(6): p. 450-459.

7. Woodroffe, R., J. Ginsberg, and D.W. Macdonald, *The African wild dog: status survey and conservation action plan*1997: IUCN.

8. IUCN/SSC, *Regional conservation strategy for the cheetah and African wild dog in Southern Africa*, 2007, IUCN Species Survival Commission Gland.

9. Woodroffe, R. and C. Sillero-Zubiri, *Lycaon pictus.* The IUCN Red List of Threatened Species 2012 (Downloaded August 2017), 2012. **e.T12436A16711116**.

10. Woodroffe, R. and J.R. Ginsberg, *Edge effects and the extinction of populations inside protected areas.* Science, 1998. **280**(5372): p. 2126-2128.

11. Courchamp, F., T. Clutton-Brock, and B. Grenfell, *Inverse density dependence and the Allee effect.* Trends in Ecology & Evolution, 1999. **14**(10): p. 405-410.

12. Courchamp, F., T. Clutton-Brock, and B. Grenfell. *Multipack dynamics and the Allee effect in the African wild dog, Lycaon pictus*. in *Animal Conservation forum*. 2000. Cambridge University Press.

13. McNutt, J.W. and J.B. Silk, *Pup production, sex ratios, and survivorship in African wild dogs, Lycaon pictus.* Behavioral Ecology and Sociobiology, 2008. **62**(7): p. 1061-1067.

14. McNutt, J.W., *Sex-biased dispersal in African wild dogs, Lycaon pictus.* Animal behaviour, 1996. **52**(6): p. 1067-1077.

15. Fanshawe, J.H. and C.D. Fitzgibbon, *Factors influencing the hunting success of an African wild dog pack.* Animal behaviour, 1993. **45**(3): p. 479-490.

16. Creel, S. and N.M. Creel, *Six ecological factors that may limit African wild dogs, Lycaon pictus.* Animal Conservation, 1998. **1**(1): p. 1-9.

17. Creel, S. and N.M. Creel, *Opposing effects of group size on reproduction and survival in African wild dogs.* Behavioral Ecology, 2015. **26**(5): p. 1414-1422.

18. Girman, D., et al., *Patterns of population subdivision, gene flow and genetic variability in the African wild dog (Lycaon pictus).* Molecular Ecology, 2001. **10**(7): p. 1703-1723.

19. Marsden, C.D., et al., *Spatial and temporal patterns of neutral and adaptive genetic variation in the endangered African wild dog (Lycaon pictus).* Molecular Ecology, 2012. **21**(6): p. 1379-1393.

20. Marsden, C.D., et al., *Highly endangered African wild dogs (Lycaon pictus) lack variation at the major histocompatibility complex.* Journal of heredity, 2009. **100**(suppl_1): p. S54-S65.

21. Campana, M.G., et al., *Genome sequence, population history, and pelage genetics of the endangered African wild dog (Lycaon pictus).* BMC genomics, 2016. **17**(1): p. 1013.

22. Shapiro, B. and M. Hofreiter, *A paleogenomic perspective on evolution and gene function: new insights from ancient DNA.* Science, 2014. **343**(6169): p. 1236573.

23. Nyakatura, K. and O.R. Bininda-Emonds, *Updating the evolutionary history of Carnivora (Mammalia): a new species-level supertree complete with divergence time estimates.* BMC biology, 2012. **10**(1): p. 12.

24. Lander, E.S., et al., *Initial sequencing and analysis of the human genome.* 2001.

25. Hayden, E.C., *The $1,000 genome.* Nature, 2014. **507**(7492): p. 294.

26. Goodwin, S., J.D. McPherson, and W.R. McCombie, *Coming of age: ten years of next-generation sequencing technologies.* Nature Reviews Genetics, 2016. **17**(6): p. 333-351.

27. Ekblom, R. and J.B. Wolf, *A field guide to whole‐genome sequencing, assembly and annotation.* Evolutionary applications, 2014. **7**(9): p. 1026-1042.

28. Putnam, N.H., et al., *Chromosome-scale shotgun assembly using an in vitro method for long-range linkage.* Genome Research, 2016. **26**(3): p. 342-350.

29. Burton, J.N., et al., *Chromosome-scale scaffolding of de novo genome assemblies based on chromatin interactions.* Nature biotechnology, 2013. **31**(12): p. 1119-1125.

30. Weisenfeld, N.I., et al., *Direct determination of diploid genome sequences.* Genome Research, 2017. **27**(5): p. 757-767.

31. Lok, S., et al., *De novo genome and transcriptome assembly of the Canadian beaver (Castor canadensis).* G3: Genes, Genomes, Genetics, 2017. **7**(2): p. 755-773.

32. Liu, S., et al., *Population Genomics Reveal Recent Speciation and Rapid Evolutionary Adaptation in Polar Bears.* Cell, 2014. **157**(4): p. 785-794.

33. Huang, J., et al., *Analysis of horse genomes provides insight into the diversification and adaptive evolution of karyotype.* Scientific reports, 2014. **4**.

34. Gnerre, S., et al., *High-quality draft assemblies of mammalian genomes from massively parallel sequence data.* Proceedings of the National Academy of Sciences, 2011. **108**(4): p. 1513-1518.

35. Mohr, D.W., et al., *Improved de novo Genome Assembly: Linked-Read Sequencing Combined with Optical Mapping Produce a High Quality Mammalian Genome at Relatively Low Cost.* bioRxiv, 2017: p. 128348.

36. Frantzen, M., et al., *Empirical evaluation of preservation methods for faecal DNA.* Molecular Ecology, 1998. **7**(10): p. 1423-1428.

37. Taberlet, P. and G. Luikart, *Non-invasive genetic sampling and individual identification.* Biological Journal of the Linnean Society, 1999. **68**(1-2): p. 41-55.

38. Morin, P.A., G. Luikart, and R.K. Wayne, *SNPs in ecology, evolution and conservation.* Trends in Ecology & Evolution, 2004. **19**(4): p. 208-216.

39. Vieira, F.G., et al., *Estimating inbreeding coefficients from NGS data: impact on genotype calling and allele frequency estimation.* Genome Research, 2013. **23**(11): p. 1852-1861.

40. Robinson, J.A., et al., *Genomic flatlining in the endangered island fox.* Current Biology, 2016. **26**(9): p. 1183-1189.

41. Pazmiño, D.A., et al., *Genome-wide SNPs reveal low effective population size within confined management units of the highly vagile Galapagos shark (Carcharhinus galapagensis).* Conservation Genetics, 2017: p. 1-13.

42. Hampton, J.O., et al., *Molecular techniques, wildlife management and the importance of genetic population structure and dispersal: a case study with feral pigs.* Journal of Applied Ecology, 2004. **41**(4): p. 735-743.

43. Abascal, F., et al., *Extreme genomic erosion after recurrent demographic bottlenecks in the highly endangered Iberian lynx.* Genome biology, 2016. **17**(1): p. 251.

44. Dobrynin, P., et al., *Genomic legacy of the African cheetah, Acinonyx jubatus.* Genome biology, 2015. **16**(1): p. 277.

45. Kim, S., et al., *Comparison of carnivore, omnivore, and herbivore mammalian genomes with a new leopard assembly.* Genome biology, 2016. **17**(1): p. 211.

46. Gurevich, A., et al., *QUAST: quality assessment tool for genome assemblies.* Bioinformatics, 2013. **29**(8): p. 1072-1075.

47. Simão, F.A., et al., *BUSCO: assessing genome assembly and annotation completeness with single-copy orthologs.* Bioinformatics, 2015. **31**(19): p. 3210-3212.

48. Hoeppner, M.P., et al., *An Improved Canine Genome and a Comprehensive Catalogue of Coding Genes and Non-Coding Transcripts.* PLoS one, 2014. **9**(3): p. e91172.

49. Jurka, J., et al., *Repbase Update, a database of eukaryotic repetitive elements.* Cytogenetic and genome research, 2005. **110**(1-4): p. 462-467.

50. Holt, C. and M. Yandell, *MAKER2: an annotation pipeline and genome-database management tool for second-generation genome projects.* BMC bioinformatics, 2011. **12**(1): p. 491.

51. Lechner, M., et al., *Orthology detection combining clustering and synteny for very large datasets.* 2014.

52. Li, H. and R. Durbin, *Fast and accurate short read alignment with Burrows–Wheeler transform.* Bioinformatics, 2009. **25**(14): p. 1754-1760.

53. Li, H., et al., *The sequence alignment/map format and SAMtools.* Bioinformatics, 2009. **25**(16): p. 2078-2079.

54. Korneliussen, T.S., A. Albrechtsen, and R. Nielsen, *ANGSD: analysis of next generation sequencing data.* BMC bioinformatics, 2014. **15**(1): p. 356.

## Tables

**Table 1. Assembly Statistics.** Assembly statistics for the three African wild dog genomes reported by the Supernova assembler. Coverage was assessed using samtools depth.

| | | Sister 1 | Sister 2 | Eureka |
|---|---|---|---|---|
| Input | Reads (m) | 1,200 | 801.56 | 427.6 |
| | Average coverage | 69 | 46 | 25 |
| | Mean molecule size (kb) | 19.91 | 77.03 | 52.00 |
| Contig | N50 (kb) | 61.34 | 83.47 | 50.15 |
| | Longest (kb) | 524.60 | 615.40 | 450.50 |
| | Number (k) | 78.62 | 68.64 | 108.00 |
| Scaffold | N50 (mb) | 7.91 | 21.34 | 15.31 |
| | Longest (kb) | 43.96 | 69.63 | 41.67 |
| | Number (k) | 11.78 | 17.64 | 25.78 |
| Total size (gb) | Scaffolds >= 10kb | 2.27 | 2.26 | 2.20 |
| | Scaffolds >= 500bp | 2.34 | 2.40 | 2.42 |

**Table 2. Conserved Gene Statistics.** Results of the BUSCO v2 gene annotation from three African wild dog genome assemblies, canFam3.1, low-coverage wild dog genomes [21], and the recently published Hawaiian Monk seal genome [35].

| Assembly | Species | Complete | Single copy | Duplicated | Fragmented | Missing | Total searched |
|---|---|---|---|---|---|---|---|
| Sister 1 | *L. pictus* | 3914 | 3875 | 39 | 102 | 88 | 4104 |

| | | | | | | | |
|---|---|---|---|---|---|---|---|
| Sister 2 | *L. pictus* | 3903 | 3845 | 58 | 107 | 94 | 4104 |
| Eureka | *L. pictus* | 3829 | 3789 | 40 | 169 | 106 | 4104 |
| canFam3.1 | *C. familiaris* | 3910 | 3857 | 53 | 98 | 96 | 4104 |
| Kenya | *L. pictus* | 3849 | 3823 | 26 | 136 | 119 | 4104 |
| South Africa | *L. pictus* | 3892 | 3867 | 25 | 104 | 108 | 4104 |
| Hawaiian monk seal | *Neomonachus schauinslandi* | 3881 | 3833 | 48 | 118 | 105 | 4104 |

Figure 1 Click here to download Figure AWD_figure1.pdf

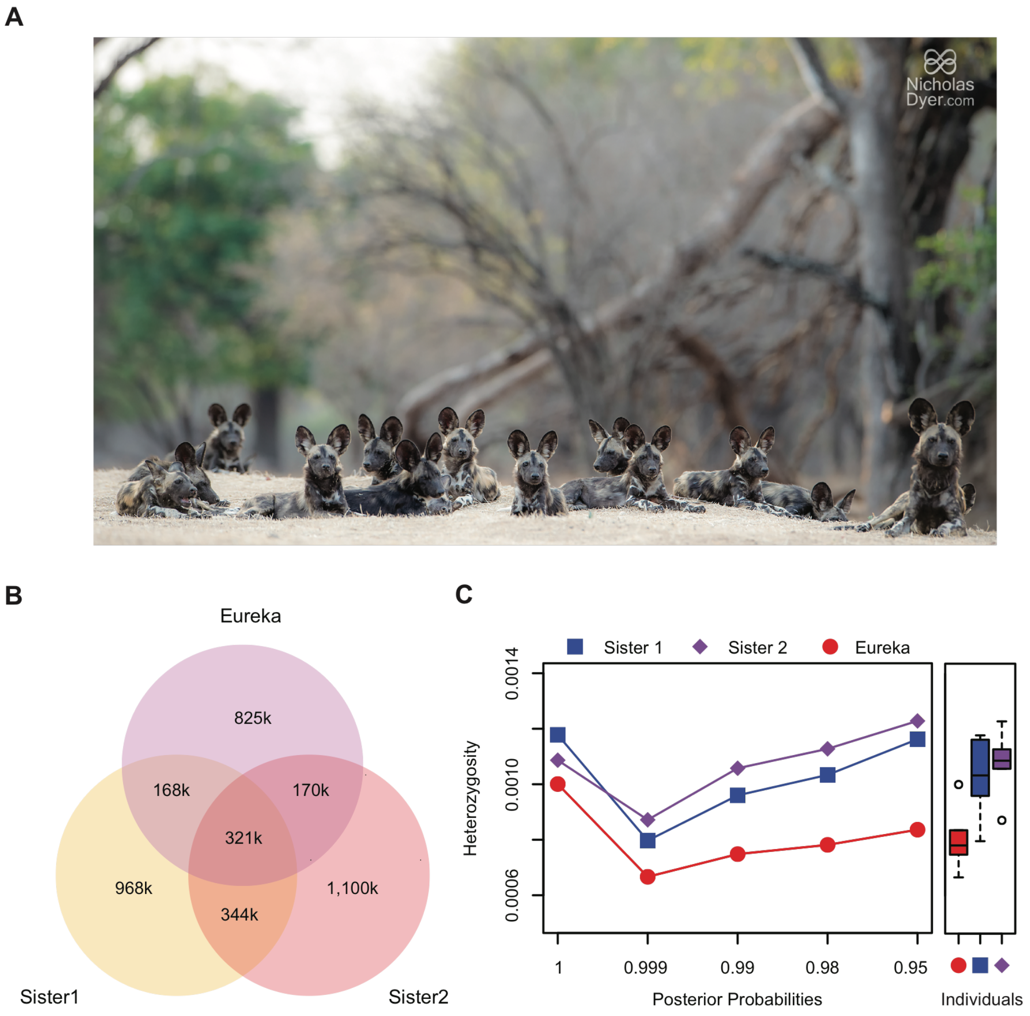


Figure 1. Shared heterozygous sites between the different African wild dog individuals. A) Pack of African wild dogs. B) Shared heterozygous sites between the three *de novo* assemblies (calculated using a posterior cutoff of 0.99). Many of the heterozygous sites are shared between all individuals and more heterozygous sites are shared between the two sisters than between each sister and Eureka. C) Comparison of heterozygosity estimates using different posterior probability cutoffs for all three assemblies. Boxplot of heterozygosity values (y-axis) calculated for different posterior probability cutoffs.

Supporting information for methods, gene and repeat annotations,
and heterozygosity calculations.

Click here to access/download
**Supplementary Material**
Supporting_information_AWD_Gigascience.docx

Personal Cover
Click here to download Personal Cover awd_coverletter_final_giga.pdf

Stanford University

Stanford, 28th of November 2017

Dear Editor,

We would like to submit our manuscript titled "Entering the era of conservation genomics: Cost-effective assembly of the African wild dog genome using linked reads." for consideration for publication in GigaScience.

The Anthropocene is currently impacting species across the globe at an unprecedented rate. Managers and scientists are searching for cost-effective methods to monitor population level changes and understand past distributions and adaptations to best plan for the future. Genomic tools can inform many of these questions and assist in developing SNP assays for monitoring, but historically genome sequencing has been problematic both because of its high cost and sample submission requirements. Thus, the demonstration of a system that can circumvent these issues is critical.

Here we present the results of the 10x Genomics Chromium platform for genome assembly of an endangered canid, the African wild dog (*Lycaon pictus*). Our work is the first *de novo* assembly for this species and demonstrates that this method of genome sequencing generates comparable or better assemblies than traditional Illumina based paired-end and mate-paired sequencing for a small fraction of the cost. We find that the results of the 10x Chromium technology are consistent and reproducible across the three individuals we sequenced. Additionally, we show that the wild dog has a higher heterozygosity than expected given its endangered status, which may be an outcome of its social biology.

The African wild dog, like many other endangered species, is being heavily targeted for recovery. Important measures to consider for such recovery programs will be statistics such as inbreeding and heterozygosity, and also a better understanding of the demography of the wild dogs. A well-assembled reference genome provides the basis for which these measures can be estimated using low-coverage population sequencing. As endangered species often have less genomic resources than model organisms, 10x Genomics Chromium assemblies will open up a new avenue of study without impacting conservation dollars that must be put towards on the ground monitoring.

In conclusion, we show that the 10x Genomics Chromium system produces a highly continuous and quality genome assembly for comparably less coverage and cost than other technologies. This work is particularly relevant for species of conservation concern that could benefit from genetic monitoring and in-depth studies of adaptive capabilities to mounting pressures such as climate change and shrinking population sizes.

We recently uploaded a pre-print to BioRxiv and the response has been overwhelmingly positive. We feel strongly that the manuscript is a good fit to the scope and mission of the GigaScience journal. We hope that it will reach a broad audience and facilitate the expansion of high-quality genomic datasets from many organisms.

We hope you find our manuscript of interest and look forward to hearing about your decision.

Sincerely,
Ellie Armstrong
