# Supplementary material for: Cost-effective assembly of the African wild dog (Lycaon pictus) genome using linked reads
Source: Gigascience. 2018 Oct 22;8(2):giy124. doi: 10.1093/gigascience/giy124 (PMC6350039; doi:10.1093/gigascience/giy124)
Supplement: GIGA-D-17-00324_Revision_1.pdf [file giy124_giga-d-17-00324_revision_1.pdf]

GigaScience

Cost-effective assembly of the African wild dog genome using linked reads.

--Manuscript Draft--

**Manuscript Number:**	GIGA-D-17-00324R1
**Full Title:**	Cost-effective assembly of the African wild dog genome using linked reads.
**Article Type:**	Research
**Funding Information:**	John Stuelpnagel — Not applicable
**Abstract:**	A high-quality reference genome assembly is a valuable tool for the study of non-model organisms. Genomic techniques can provide important insights about past population sizes, local adaptation, and aid in the development of breeding management plans. This information is important for fields like conservation genetics, where endangered species require critical and immediate attention. However, funding for genomic-based methods can be sparse for conservation projects, as costs for general species management can consume budgets. Here we report the generation of high-quality reference genomes for the African wild dog (Lycaon pictus) at a low cost (< $3000), thereby facilitating future studies of this endangered canid. We generated assemblies for three individuals using the linked-read 10x Genomics Chromium system. The most continuous assembly had a scaffold and contig N50 of 21 Mb and 83 Kb, respectively, and completely reconstructed 95% of a set of conserved mammalian genes. Additionally, we estimate the heterozygosity and demographic history of African wild dogs, revealing that although they have historically low effective population sizes, heterozygosity remains high. We show that 10x Genomics Chromium data can be used to effectively generate high-quality genomes from Illumina short-read data of intermediate coverage (~25-50x). Interestingly, the wild dog shows higher heterozygosity than other species of conservation concern, possibly due to its behavioral ecology. The availability of reference genomes for non-model organisms will facilitate better genetic monitoring of threatened species such as the African wild dog and help conservationists to better understand the ecology and adaptability of those species in a changing environment.
**Corresponding Author:**	Ellie Armstrong<br>Stanford University<br>UNITED STATES
**Corresponding Author Secondary Information:**	
**Corresponding Author's Institution:**	Stanford University
**Corresponding Author's Secondary Institution:**	
**First Author:**	Ellie Armstrong
**First Author Secondary Information:**	
**Order of Authors:**	Ellie Armstrong
	Ryan W Taylor
	Stefan Prost
	Peter Blinston
	Esther van der Meer
	Hillary Madzikanda
	Olivia Mufute
	Roseline Madisodza-Chikerema
	John Stuelpnagel
	Claudio Sillero-Zubiri

Powered by Editorial Manager® and ProduXion Manager® from Aries Systems Corporation

	Dmitri Petrov
**Order of Authors Secondary Information:**	
**Response to Reviewers:**	Response to Editor Comments<br><br>We have included additional commentary regarding the sample preparation and processing. We have discussed further reasons for possible differences between the assemblies, as well as noted which parameters we are unable to investigate as a result of this study (e.g. the relationship between estimated molecule input length and percent genome phased). We have also changed the title, as requested.<br><br>Reviewer 1<br><br>Discretionary Revision: Perhaps it would be useful to run a PSMC-type analysis using multiple wild dog genomes to assess trends in historical population sizes in recent times for African wild dogs. This might produce useful results with conservation applications. There are several methods that have come out recently that can do a decent job with estimating population size in recent times.<br><br>We have added a PSMC analyses of our three genomes. The results show comparative historical population sizes to those estimated in Campana et al. (2016) (Figure 1). The most notable differences are in the recent population size estimates and the timing of the beginning of the population decline, but are overall consistent.<br><br>Edit: Line 444. The word "Heterozygosity" at the end of the paragraph seems out of place.<br><br>This sentence has been revised.<br><br>Reviewer 2<br><br>Line 84 - 'The lineage is the only surviving member of a lineage of wolf-like canids' is I guess true to some degree, but that could be said of other wolf-like canids like the dhole, Ethiopian wolf, African Golden Wolf etc. Perhaps consider rewriting.<br><br>This sentence and others have been revised as suggested from this comment, as well as comments from Reviewer 3 to reflect more accurate predictions of the divergence of the African wild dog lineage from other canids. We have included more up to date estimates for this timing.<br><br>Line 171 and elsewhere, term 'high quality' is used. I agree that the scaffold size is excellent, but high quality also can refer to long contig sizes (in particular if one wants to study repeats, duplication etc). It would be useful if the authors could undertake a comparison of the contig sizes recovered here to those other genomes of similar SCAFFOLD quality (in particular genomes generated with different methods) so that readers can get a feel for how the contig size varies when using this approach as opposed to much more expensive methods (e.g. deep PacBio sequencing, or mate pair Illumina). Of the top of my head, one comparison in this regard could be to look at the recently published purely Illumina (mate pair) based wolf de novo genome (Gopalakrishnan et al. 2017 BMC Genomics). Unfortunately that genome is not annotated so other comparisons cannot be made (e.g. gene completeness) but simply what I suggest would be interesting.<br><br>We have added an analyses comparing contig and scaffold sizes of our genomes with the wolf genome. We ran analyses on all genomes using the Assemblathon scripts (Table S2) and BUSCO v2 (Table 2). We also annotated the wolf genome for comparison of gene completeness with the same methods as we annotated the African wild dog genomes.<br><br>Line 360-361 - perhaps give sequencing price per GB or per 100GB instead of per lane? As many readers may not know the lane output.<br><br>We have noted the output of the sequencer and hope this provides a reference to the

Powered by Editorial Manager® and ProduXion Manager® from Aries Systems Corporation

reader.

Reviewer 3

We especially thank Reviewer 3 for their extensive time and comments to our manuscript. Below we have outlined responses to these comments, as well as clarification on certain aspects of the manuscript.

1. Lines 1-2: The title should be revised because we've already been in the 'era of conservation genomics' for several years now, so this idea is out of date. How about just shortening the title to: "Cost-effective assembly of the African wild dog (Lycaon pictus) genome using linked reads"

Revised as suggested.

2. Line 80: Add a comma after "Taken together" so that the sentence reads: "Taken together, genomic tools are poised…"

Revised as suggested.

3. Line 82: "The African wild dog…" The species is also known by two other common names that are commonly applied to Lycaon pictus - African painted dog and Cape hunting dog. The former is especially used by many researchers and canid conservationists. Therefore, the authors should include these alternative names: "The African wild dog, also known as the African painted dog or Cape hunting dog (Lycaon pictus) is a medium-sized (18-34kg)…"

Revised as suggested.

4. Line 83: "sub Saharan should by hyphenated.

Revised as suggested.

5. Lines 123-125: "The groups containing the African wild dog and the domestic dog…" The authors cite the Nyakatura and Bininda-Emonds (2012) paper on the updated supertree analyses of the Carnivora to support the phylogenetic grouping and divergence time of the African wild dog in relation to the domestic dog. However, the supertree results are inconsistent with more direct assessments of canid phylogenetic history based on analyses of DNA sequences from multiple nuclear and mitochondrial loci. Supertree analyses have been empirically shown to produce inaccurate results regarding relationships. Direct assessment of DNA sequences indicate that the African wild dog and domestic dog, its wild counterpart, the gray wolf, and other wolf-like canids, are grouped together in the same clade (Tribe Canini, the wolf-like-canids). Furthermore, recent estimates of divergence times suggest that the African wild dog lineage and domestic dog lineage split only about 2.5 - 4 Mya (less than have the age suggested by Nyakatura and Bininda-Emonds, 2012). The authors should instead cite the following references: Lindblad-Toh et al. 2005 Nature 438: 803; Perini et al. 2010 Journal of Evolutionary Biology 23: 311; . The authors should then revise this sentence accordingly.

Associated sentences revised and inferences revised accordingly.

6. Lines 138-139: "…it has been impossible to assemble highly-contiguous genomes from only these short sequences." This statement is incorrect, in particular, the use of the word "impossible." Many mammalian genome assemblies with high continuity (e.g., human, dog, cow, Tasmanian devil, cheetah) have been generated using Illumina short read data. Short read data per se is not the problem. Given that enough paired-end shotgun and mate pair libraries are constructed and sequenced, the resulting short read data can be assembled to produce draft assemblies with high continuity despite the high content of repetitive sequences (comparable to or greater than those generated by the 10X Genomics Chromium System). Therefore, the comparison is a relative one and mostly depends on input. I suggest the authors revise the sentence as follows: "Because large proportions of typical mammal genomes consist of repetitive sequences, it has been challenging to obtain complete or highly

continuous genome assemblies using only these short sequences."

Revised as suggested.

7. Lines 173-175: "Thus, in order for it to be useful for conservation purposes the technology needs to be (a) cost-effective and (b) user-friendly." This sentence doesn't make sense and doesn't accord with the facts. Genomes of multiple endangered species (e.g., tiger - Cho et al. 2013 Nat Comm; crested ibis - Li et al 2014 Genome Biol.; cheetah - Dobrynin et al. 2015 Genome Biol.' Iberian lynx - Abascal et al. 2016 Genome Biol.) have been generated and directly useful for conservation purposes regardless of their cost-effectiveness or user-friendliness. The authors' statement precludes other potential sequencing technologies that may not be as cost-effective (e.g. PacBio long reads) but yet still may be used to obtain high quality genome assemblies for conservation genomic applications. And most surprisingly, why should user-friendliness with regards to analysis of next generation sequencing data (i.e., bioinformatics) ever be a criterion on whether it is useful or not for conservation? Please delete this sentence.

We have revised this sentence with an emphasis on the practicality of using genomics as a wide-spread tool in the conservation world. We would defend that it still remains elusive or out of reach for many conservation biologists to assemble a genome de novo, despite desiring to use what a reference assembly provides downstream for everyday conservation practice. We direct the reviewers to a recent study (Taylor et al. (2017) Bridging the conservation genetics gap by identifying barriers to implementation for conservation practitioners. Global Ecology and Conservation), which describes a common disconnect between managers desiring to use genetic and genomic resources, but lacking the funds and expertise to use such technologies.

8. Line 184: "and are presumed to be sisters…" The authors should indicate that the details behind this presumption are included in the supporting information and cite Appendix S1.

Revised accordingly.

9. Lines 202 - 204: The authors need to cite Hoeppner et al. 2014 here; e.g., "…from the most recent dog genome (267kb and 45.9Mb, respectively [48]),"

Revised accordingly.

10. Line 216: Same comment as point 9; need to cite the Hoeppner et al. 2014 paper.

Revised accordingly.

11. Lines 240-241: "Furthermore, repeat content of all wild dog assemblies was qualitatively similar to canFam3.1." Given that African wild dog and domestic dog share a relatively close evolutionary ancestry (see point #5 above), it's not surprising that their repeat contents would be similar. The authors should qualify their findings in these terms.

Revised accordingly.

12. Lines 242-245: "…the similarity in repeat content between the African wild dog compared to that of the domestic dog, highlights the value of using 10x Genomics Chromium technology to produce accurate and continuous assemblies." This seems like a specious conclusion. The canFam3.1 assembly was not generated using 10x Genomics data, yet it has a repeat content similar to the African wild dogs. This is likely due to the recent common ancestry (point #11) and not because of the technology used to sequence/assemble the genome. The repeat content of the two species would be similar regardless of the continuity of the assembly or how that was achieved. I recommend the authors delete the last sentence in this paragraph.

Revised as suggested.

13. Line 254: "…multi copy…" should be hyphenated (multi-copy).

Powered by Editorial Manager® and ProduXion Manager® from Aries Systems Corporation

Revised as suggested.

14. Line 255: "…and 37 not present in one individual." Specify which individual was missing these multi-copy genes (paralogues). Any reason why these 37 multi-copy genes were missing? Lower coverage? Assembly problem?

We re-phrased this sentence to more accurately reflect the results. Thirty-seven total singletons were missing across the three individuals, with the lowest coverage genome missing the most and the highest coverage genome missing the least.

15. Lines 270-272: "As expected, we see a higher number of singletons in these two individuals…" Here the authors should be more explicit about the discrepancy in the number of singleton SNPs in the two African wild dogs sequenced by Campana et al. 2016 and the three individuals sequenced by the authors. Please provide numbers or percentages about the differences and then cite the Appendix S1 for the detailed methods used for variant calling. Coverage in and of itself may not be the sole reason for the higher number of singletons in the two African wild dogs sequenced by Campana et al. More stringent filtering methods applied to these two individuals would likely have resulted in a comparable number of SNPs to the three individuals sequenced by the authors. The authors should discuss these alternatives. Also, the Nielsen et al. 2011 and 2012 references are not included in the references (main text or Appendix S1). Also, the authors should consider the following papers: Bryc et al. 2013 Genetics 195: 553 and Kousathanas et al. 2017 Genetics 205: 317.

We agree with the reviewer that there is much to be said for the different ways to estimate heterozygosity, but would add that this is difficult to do without introducing additional biases. Indeed, data-preprocessing, the choice of a reference genome (this particular issue is documented in Gopalakrishnan et al. 2017 using the wolf data), mapping tools, and filtering, may all introduce unknown biases in heterozygosity estimates. Our intention in this paper was not to estimate heterozygosity using multiple different methods, but rather use a single method and estimate differences. However, this would be a pertinent follow-up study in the future using a more controlled data set and we will certainly consider this. We have adjusted the language here to acknowledge the limitations of our analyses.

16. Lines 280-281: "Our estimates show that, while being heavily threatened, African Wild
dogs seem to still retain a relatively high within individual heterozygosity." First "Wild" in this sentence should be revised as "wild." Second, the conclusion of "relatively high within individual heterozygosity" is impossible to judge without context to some reference/metric or other species. Relative to what exactly? The per site heterozygosities measured by the authors should be compared to those obtained from other species listed as endangered or critically endangered on the IUCN Red List. The paper by Robinson et al. 2016 Current Biol. 26: 1183 would be of use for this. Furthermore, it would be useful to compare the per site heterozygosities obtained for the three African wild dogs with those of gray wolves reported by Gopalakrishnan et al. 2017 BMC Genomics 18: 495 (see their Table S1).

We have included comparisons to those reported for several endangered species in Dobrynin et al. 2016, Gopalakrishnan et al. 2017, and Robinson et al. 2016.

17. Lines 299-301: "This may indicate that input molecule length is a key factor for scaffolding, while coverage is a key factor for contig assembly." Input molecule length is indeed likely to have a strong effect on assembly quality for the 10X Genomics platform. In fact, this is directly stated by 10X Genomics: "DNA quality. By far the most common cause of subpar assembly results is poor input DNA quality" (https://support.10xgenomics.com/de-novo-assembly/software/pipelines/latest/troubleshooting). In fact, the Chromium library preparation process may nick the DNA and thus cause fragmentation (smaller molecule lengths). The authors should include and cite the weblink above. It is somewhat surprising that the assemblies of the three African wild dogs were so different in terms of their assembly metrics (e.g., contig and scaffold N50s). Given that the 10X Genomics linked-read technology is still relatively new, it's difficult to judge

Powered by Editorial Manager® and ProduXion Manager® from Aries Systems Corporation

whether these results are common or not. The authors' findings do not accord with my own experience using 10X, where assembly metrics from multiple individuals of the same species were more consistent (mostly identical). The authors should discuss in in one or two additional sentences other factors that may have influenced their results: 1) sample handling, storage, and/or preparation; 2) library preparation - were the three libraries prepared by the same lab or technician? The authors state in Appendix S1 that the three individuals were sequenced at two different sequencing facilities/vendors; 3) sequencing platforms, chemistries used (HiSeq X for two individuals vs. HiSeq4000 for the third).

We have included the link as part of our revisions and added this as a commentary. We do emphasize that the three assemblies were sequenced at different depths, which may also result in some of the stochasticity among our assemblies. We hope that what comes across is not that the assemblies are wildly different, but rather that as an assembly service which is cost-effective, that the results across individuals are more or less consistent.

18. Lines 357-375: Cost effectiveness: The authors should list the US sequencing facilities examined and their corresponding prices for Chromium library preparation and sequencing in the Supporting Information- Appendix 1 in a table. This will provide readers with the explicit information to gauge different costs associated with these services. This information is also usually provided on the websites of sequencing facilities and vendors. Also, the authors should indicate the pricings for the library preparation and sequencing at the two sequencing facilities they used to generate the data of the three African wild dogs. Also, how much would the cost be for if the authors had used generated and sequenced Illumina shotgun and mate pair libraries to obtain genome assemblies comparable in quality to those generated using the 10X Chromium platform?

We have included details on the prices we paid for each assembly. We are reluctant to include a survey of current costs because the cost for sequence services changes rapidly, and the prices posted on websites are not always representative of negotiated prices. We believe the prices we paid are within 15% of prices currently offered by most sequencing providers.

We have more explicitly listed the cost of each of our genomes by their components (the price of a lane and the price of the library prep) in comparison with the approximate cost to prepare the libraries and sequencing of the wolf genome, a comparable Illumina library based genome.

19. Lines 408-411: See my previous comments with respect to this issue in point # 15 above. It would be useful to cite Nielsen et al. 2011 and 2012 here.

We have incorporated the Nielsen et al. 2011 & 2012 citations where appropriate. We thank the reviewer for bringing this oversight to our attention.

20. Line 414: "other threatened large bodied carnivores…" - Neither the Iberian lynx nor dwarf Channel island fox would be considered large-bodied. I suggest the authors revise this just as: "other threatened carnivores…"

Revised as suggested.

21. Line 421: a comma should be added after "dogs" in this sentence.

Revised as suggested.

22. Line 433: "…as part of the assembly process, however, when the fasta consensus sequence…" This is a run-on sentence and should be broken into two sentences: "…as part of the assembly process. However, when the fasta consensus sequence…"

Revised as suggested.

23. Line 473: "DNA was extracted 9 days after the sample was taken." The authors should provide details about how this sample was stored prior to DNA extraction. Also,

what type of blood tubes (e.g., Vacutainer) were the samples collected into? These details are important to document given the importance of the HMW input DNA to the success of the 10X Genomics Chromium technology (and in the interests of reproducibility).

We had described the storage and processing of the samples in detail, but failed to reference appendix S1. We have corrected this error.

24. Line 486 (and in Appendix S1): In the interests of reproducibility, the default assembly parameters should be listed or described.

There are no assembly parameters for Supernova and it is simply ‘supernova run’ in the same directory as the fastq files.

25. Line 492: "lineage specific" should include a hyphen.

Revised as suggested.

26. Line 496: "BAC end" should include a hyphen.

Revised as suggested.

27. Lines 524-527: The 10X Genomics Supernova assembler outputs four FASTA data files (raw, megabubbles, pseudohap and pseudohap2); see: https://support.10xgenomics.com/de-novo-assembly/software/pipelines/latest/output/generating. Given that there are only two outputs that provide the phased information (pseudohap and pseudohap2), how could this choice for estimating heterozygosity possibly be described a random? In the interests of reproducibility, the authors should indicate which pseudo-haplotype file was used for which individual African wild dog. Also, the authors should at least take one individual (Sister 2, the one with the most continuous assembly) and estimate the heterozygosity from the other pseudo-haplotype file to check that there is no difference in the inferred number of heterozygous sites (this acts as a control).

We have included an analysis of the two distinct pseudohaplotypes from the --style=pseudohap2 output for Sister 2 and have included a more thorough description of which files were used for each. We do note, however, that the software does a randomized pseudohaplotype when the option --style=pseudohap is chosen and is noted here in the Supernova manual: “For pseudohap...Megabubble arms are chosen arbitrarily so many records will mix maternal and paternal alleles.” However, for --style=pseudohap2, the maternal and paternal arms are separated. We have made efforts to make this more clear in the text.

28. Line 529: The Samtools and Picard programs should be capitalized.

Revised as suggested.

29. Literature cited: The authors should carefully check the formatting of their references so that they consistently conform to the journal standards (e.g., journal titles are often not properly capitalized).

Revised as suggested.

30. Methods (main text and Appendix S1): Samples. Given the requirement of input DNA with long molecule lengths and its importance to the 10X Genomics technology, no details or information is provided on how the HMW genomic DNA was assayed following extraction. This is absolutely crucial and related to the issue of experimental reproducibility. Such HMW DNA is usually assessed using pulse-field electrophoresis techniques or variations thereof. Since the authors used two different sequencing facilities to generate the libraries and sequencing data, different methods may have been used for the assays. In any case, the authors should provide the details about how the HMW DNA was assessed and evaluated prior to Chromium library preparation.

Powered by Editorial Manager® and ProduXion Manager® from Aries Systems Corporation

	We have added additional information on the assays performed following extraction in the supplement.<br><br>31. Phased assemblies: Even though the percentage of the assemblies that were phased is presented in Table S1, this feature is never discussed in detail in the main text. However, this is one of the most noteworthy (and marketed) features of the 10X Genomics platform. Phased assemblies also have a dramatic impact on the downstream population genetic analyses and provide additional information for these analyses compared to technologies that do not yield phased assemblies. The authors should include a description of the phasing results of the three African wild dog assemblies in the Data Description & Analyses section as well as discuss this important feature of the 10X Genomics platform.<br><br>We considered this point extensively during analyses, but unfortunately are not able to address this point with the data in hand. Although we can produce phased vcf files, the genomes produced from the Sister 1 and Sister 2 individuals by independent Supernova runs are still too fragmented for us to consider the phasing of any certain haplotype or position, nor to investigate whether the sisters share the expected amount of variation. We are continuing this project with population-level sequencing of individuals from Zimbabwe and hope to address this point further when we have additional information on the expected allele frequencies.
**Additional Information:**	
**Question**	**Response**
Are you submitting this manuscript to a special series or article collection?	No
**Experimental design and statistics**<br><br>Full details of the experimental design and statistical methods used should be given in the Methods section, as detailed in our Minimum Standards Reporting Checklist. Information essential to interpreting the data presented should be made available in the figure legends.<br><br>Have you included all the information requested in your manuscript?	Yes
**Resources**<br><br>A description of all resources used, including antibodies, cell lines, animals and software tools, with enough information to allow them to be uniquely identified, should be included in the Methods section. Authors are strongly encouraged to cite Research Resource Identifiers (RRIDs) for antibodies, model organisms and tools, where possible.	Yes

Powered by Editorial Manager® and ProduXion Manager® from Aries Systems Corporation

Have you included the information requested as detailed in our Minimum Standards Reporting Checklist?	
**Availability of data and materials**<br><br>All datasets and code on which the conclusions of the paper rely must be either included in your submission or deposited in publicly available repositories (where available and ethically appropriate), referencing such data using a unique identifier in the references and in the “Availability of Data and Materials” section of your manuscript.<br><br>Have you have met the above requirement as detailed in our Minimum Standards Reporting Checklist?	Yes

Manuscript
Click here to view linked References
Click here to access/download;Manuscript;AWD_gigascience_main_final_4_

Cost-effective assembly of the African wild dog (*Lycaon pictus*) genome using linked reads.

Ellie E. Armstrong[1]*, Ryan W. Taylor[1]*, Stefan Prost[1,2], Peter Blinston[3], Esther van der Meer[3], Hillary Madzikanda[3], Olivia Mufute[4], Roseline Mandisodza-Chikerema[4], John Stuelpnagel[5], Claudio Sillero-Zubiri[6], Dmitri Petrov[1]

[1]Program for Conservation Genomics, Department of Biology, Stanford University, Stanford, CA, USA

[2]Department of Integrative Biology, University of California, Berkeley, CA, USA

[3]Painted Dog Conservation, Dete, Zimbabwe

[4]The Zimbabwe Parks & Wildlife Management Authority, Zimbabwe

[5]10x Genomics, Inc., Pleasanton, CA

[6]Wildlife Conservation Research Unit, Zoology, University of Oxford, The Recanati-Kaplan Centre, Tubney, UK014

* These authors contributed equally to this work.

Corresponding Author: Ellie E. Armstrong (elliea@stanford.edu)

Abstract

Background

A high-quality reference genome assembly is a valuable tool for the study of non-model organisms. Genomic techniques can provide important insights about past population sizes, local adaptation, and aid in the development of breeding management plans. This information is important for fields like conservation genetics, where endangered species require critical and immediate attention. However,

funding for genomic-based methods can be sparse for conservation projects, as costs for general species management can consume budgets.

Findings

Here we report the generation of high-quality reference genomes for the African wild dog (*Lycaon pictus*) at a low cost (< $3000), thereby facilitating future studies of this endangered canid. We generated assemblies for three individuals using the linked-read 10x Genomics Chromium system. The most continuous assembly had a scaffold and contig N50 of 21 Mb and 83 Kb, respectively, and completely reconstructed 95% of a set of conserved mammalian genes. Additionally, we estimate the heterozygosity and demographic history of African wild dogs, revealing that although they have historically low effective population sizes, heterozygosity remains high.

Conclusions

We show that 10x Genomics Chromium data can be used to effectively generate high-quality genomes from Illumina short-read data of intermediate coverage (~25-50x). Interestingly, the wild dog shows higher heterozygosity than other species of conservation concern, possibly due to its behavioral ecology. The availability of reference genomes for non-model organisms will facilitate better genetic monitoring of threatened species such as the African wild dog and help conservationists to better understand the ecology and adaptability of those species in a changing environment.

Keywords

Conservation genomics, 10x Genomics Chromium, African wild dog, *Lycaon pictus*, *de novo* Assembly

Background

Major population declines have been observed in vertebrate groups over the past several hundred years, primarily due to anthropogenic change [1]. This decline has resulted in extinction rates unprecedented in recent history [1, 2]. The conservation of extant species will require major efforts in restoring and preserving habitat, along with protection, management, and investment by local stakeholders. While, by definition, all species of conservation concern exist as small populations, populations generally still retain genetic variation that was generated and maintained when population sizes were much larger.

The historic genetic variation contains signals of demographic history, gene flow, and natural selection which can inform efforts towards the long-term survival of species. In addition to signals of a species history, genetic information can be used to uncover important contemporary or very recent events and processes. Genetic markers can be used to track individual movement across landscapes either indirectly by measuring relatedness, or directly by genotyping scat or hair left by an individual as it moves. Additionally, the identification and assignment of individuals through genotyping can be an important tool for law enforcement to assign contraband and confiscated materials to their geographic origin [4]. Conservationists can also use fine grained measurements of reproductive success along with genotypes and environmental variables to gather a detailed understanding of the factors contributing to or limiting population growth, such as inbreeding depression. Taken together, genomic tools are poised to have a major contribution to conservation [5, 6].

The African wild dog, also known as the African painted dog or Cape hunting dog (*Lycaon pictus*), is a medium-sized (18-34kg), endangered carnivore that lives in scattered populations in sub-Saharan Africa (Fig. 1A). The species is a surviving member of a lineage of wolf-like canids, including other species such as the Ethiopian wolf and the dhole [7]. Wild dogs have been subject to intense recovery

efforts across their range [8, 9], but their global population is decreasing. It is estimated that only 6,600 adult wild dogs remain in 39 subpopulations [10]. The primary reasons for the species' population decline include habitat loss and fragmentation, as well as anthropogenic mortality (e.g. snaring, persecution, road kills, exposure to infectious diseases from domestic dogs) when they range beyond the borders of protected areas [8, 9, 11]. Due to their large ranges and low population densities, African wild dogs are more susceptible to these threats than most other carnivore species [9]. In addition, their complex social system and susceptibility to Allee effects appears to increase the species extinction risk [12, 13]. The dogs are obligate cooperative breeders which form packs consisting of an alpha male and female, their adult siblings, and pups and subadults from the dominant pair [14]. Subadults that have reached reproductive age disperse in single sex groups and form new packs by joining dispersing groups from the opposite sex [15]. Pack members rely on each other for hunting, breeding, and defense against natural enemies and pack size has been found to be a significant factor in determining hunting and breeding success [14, 16, 17]. When pack size becomes critically low, this dependence on helpers increases the risk of pack extinction and reduces the number of successful dispersals ([13], but see [18]).

Prior genetic studies on wild dogs using a combination of mitochondrial, microsatellite, and MHC markers have resulted in varying estimates of the start of the species decline on the African continent [19, 20]. Consistent with expectation, the data shows strong structuring among populations due to habitat fragmentation and isolation, as well as low genetic diversity within populations [20, 21]. For species that are experiencing such rapid and alarming declines, estimates that are particularly important for management decisions, such as effective population size, inbreeding and local adaptation, are greatly improved by the use of whole-genome methods. Recently, Campana and colleagues [22] sequenced low-coverage genomes of two African wild dog individuals from Kenya and South Africa, respectively, to investigate

demographic history and signatures of selection of these two separate populations. By mapping these data to the domestic dog genome, they discovered approximately 780,000 single nucleotide polymorphisms (SNPs) between their two individuals which could be used to develop SNP typing for the two populations. However, given the low coverage of their genomes (5.7-5.8x average coverage) and the small number of individuals sequenced, additional sequencing will be needed to verify the authenticity of those SNPs. Further, important structural variation can be overlooked when mapping against a reference genome from a different genus, and mapping can be hindered if the divergence is high between the sample and the reference (see e.g. [23]). The groups containing the African wild dog and the domestic dog are estimated to have split approximately 2.5-4 Mya and furthermore, the domestic dog has undergone significant genomic selection in recent time [24, 25,26].

Despite the ever-declining cost to sequence DNA, the routine use of genomic approaches in conservation is still far from a reality. One of the major remaining barriers is the lack of reference genomes for species of conservation concern. Generating a *de novo* reference genome generally requires the sequencing and assembly of billions of base pairs that make up a genome. The first mammalian genome (human) required a massive collaboration among hundreds of scientists and nearly $3 billion US dollars (1990-2001; [27, 28]). Fortunately, the cost to sequence DNA is now low enough that every base-pair in a typical mammalian genome can be sequenced to high-coverage for a few thousand US dollars. However, these low-cost sequencing methods produce very short sequences of 150-300 base-pairs in length (for a review on sequencing methods see [29]). Because large proportions of typical mammal genomes consist of repetitive sequences, it has been challenging to obtain complete or highly-contiguous genomes using only these short sequences. In order to achieve higher continuity, more elaborate and expensive library preparation or alternative sequencing technologies have to be used [29, 30]. Among others, these include mate-pair libraries, chromatin folding based libraries, such as cHiCago [31] or

HiC [32], and long-read sequencing technologies, such as Pacific Biosciences and Oxford Nanopore Technology. While the resulting genomes can show high continuity, those methods substantially increase the costs of sequencing projects and thus can hinder the generation of genomes for conservation biology purposes.

Here we report the use of the Chromium system developed by 10x Genomics [33], a genomic library preparation technique that facilitates cost-effective assemblies using short sequencing reads, to assemble three African wild dog genomes. In brief, the 10x Genomics Chromium system is based on dilution of high molecular weight (HMW) DNA. It uses as little as 1ng of input DNA, which is well-suited for a variety of applications. During library preparation, gel beads, so-called GEMs, are mixed with DNA and polymerase for whole-genome amplification. Each gel bead has primer oligos (44nt long) attached to its surface. These contain a priming site (22nt partial R1), a 16nt barcode region, and a 6nt N-mer region that binds to different places on the original DNA fragment. The low amount of input DNA ensures that each gel bead only binds a single (up to ~100kb) DNA fragment. In the next step, amplification of short reads along the original DNA fragment is performed within each gel bead. In most cases, this amplification results in spotted read coverage along the fragment. However, all reads from a respective GEM contain identical barcodes and can later be assigned to groups originating from the same DNA molecule. The information about which molecule of DNA the sequence originated from greatly increases the ability to identify the location of repetitive sequences. The library is then sequenced on an Illumina platform and the raw read data is assembled by the 10x Genomics Supernova assembler. The data produced also can be phased, presenting another potentially useful addition to genome assemblies.

We *de novo* assembled three African wild dog genomes using the 10x Genomics Chromium platform to investigate whether this technology is suitable for conservation genomic purposes. For any endangered species, a genome can enable

studies with the potential for large conservation impacts, but high-quality genomes have historically been costly or impossible due to the sampling requirements and analysis. Thus, for an assembly to be a practical component of many conservation projects, the technology needs to be (a) cost-effective and (b) user-friendly. We test the 10x Genomics Chromium based assemblies for reproducibility, continuity, conserved gene completeness, and repetitive content, as compared to the previously published domestic dog genome [34] and several other genomes built with various technologies. We further estimate heterozygosity of the individuals and within the phased data from the 10x technology and estimate historical effective population size from each genome.

Data Description & Analyses

Assembly of the African wild dog genome

Using 10x Genomics Chromium technology, we generated DNA libraries for three African wild dog individuals, two of which were collected from a wild pack in Hwange National Park, Zimbabwe and are sisters from the same litter born in June of 2013 (identified as Sister 1 and Sister 2, additional information can be found in Appendix S1), and a third unrelated individual from the Endangered Wolf Center, Eureka, Missouri (identified as Eureka). A summary of the assembly statistics output by the Supernova assembler can be found in Table 1 (detailed statistics for each genome assembly can be found in Table S1). We generated ~1.2 billion paired-end reads for Sister 1, ~0.8 billion reads for Sister 2, and ~0.4 billion reads for Eureka. We then used the reads to assemble each genome using the 10x Genomics Supernova assembler (as explained in https://support.10xgenomics.com/de-novo-assembly/software/overview/welcome). The mean input DNA molecule length reported by the Supernova assembler was 19.91kb for Sister 1, 196 77.03kb for Sister 2, and 52.00kb for Eureka. All three assemblies corroborate a genome size of

approximately 2.3Gb, which is similar to that of the domestic dog (2.4Gb; [34]). These three assemblies together constitute the first reported *de novo* assemblies for the African wild dog species.

The Sister 1 assembly resulted in a contig and scaffold N50 of 61.34 kb and 7.91 Mb, respectively, the Sister 2 assembly achieved 83.47 kb contig and 21.34 Mb scaffold N50s, and the Eureka assembly had 50.15 kb contig and 15.31 Mb scaffold N50s (Table 1). While the scaffold N50's of these three 10x genomes are are smaller than the ones from the most recent dog genome (267kb and 45.9Mb, respectively), they are still larger than most mammalian genomes assembled that used only short read data (see e.g. [36]). A recent *de novo* assembly of a wild wolf using Illumina mate-pair libraries of varying insert size resulted in a similar contig N50, but much lower scaffold N50 measurements than our results (Supporting Information Table S2; [35]). Interestingly, despite the molecule size being the highest for Sister 2, the highest percent phased data was obtained by Eureka (52.54% compared to 40.1%; Table S1).

Conserved Genes

The program BUSCO (Benchmarking Universal Single-Copy Orthologs) uses highly conserved single-copy orthologous genes from several different taxa and groups to test assemblies (both genomic and transcriptomic) for gene completeness, fragmentation, or absence as an indicator of assembly quality. Using BUSCO v2 on our assemblies, we found that the most continuous assembly, Sister 2, completely recovered 95.1% of conserved genes (Mammalia gene set; Table 2). Sister 1 and Eureka recovered 95.4% and 93.3% of complete conserved genes, respectively. Using the same analysis, we found 95.3% of complete conserved genes in the latest dog assembly (canFam3.1; [34]). This indicates that although the domestic dog assembly is more continuous overall, our assemblies recover nearly the same or even higher numbers of conserved genes. Surprisingly, Sister 1 had the fewest

missing genes out of all the assemblies assessed, despite lower continuity than Sister 2. We also ran BUSCO on the Hawaiian monk seal genome, generated through the combination of 10x Genomics Chromium and Bionano Genomics Irys data, and found it recovered 94.6% of conserved genes using BUSCO [37]. This suggests that using Bionano in addition to 10x does not greatly improve the reconstruction of the gene regions. However, the Hawaiian monk seal genome has a scaffold N50 of approximately 28Mb, so Bionano may improve the overall assembly continuity compared to 10x Genomics alone. The low-coverage genomes from Campana et al. 2016 achieved a BUSCO score of 92.8% for the individual from Kenya and 94.8% for the individual from South Africa [22]. The wolf genome also scored similarly (94.8%) [35].

Repeat annotation

We identified repetitive regions of the genome to discern how well these complex areas were assembled by the 10x Genomics Chromium technology. We found that for all three wild dog assemblies, total repeat content was evaluated to be within 3% of one another, which indicates consistency among assemblies from a single species (Supporting Information Table S3). No single repeat category was disproportionately affected during repeat annotation of the three genomes, which suggests that assembly quality was likely the most influential factor. Furthermore, repeat content of all wild dog assemblies was qualitatively similar to canFam3.1 [34] and the wolf genome [35], likely due to recent common ancestry between the two groups [24, 25, 26].

Gene annotation

Genome annotation resulted in very similar numbers of annotated genes between all three African wild dog individuals and the domestic dog [34]. Annotations ranged from 20,649 (Sister 2) to 20,946 (Sister 1) genes (Supporting Information

Table S4). Through detecting orthologous genes between individuals and paralogous genes within individuals, we found 12,617 one:one orthologs present in all three individuals and 6,462 one:one orthologs in two out of the three individuals. We found 268 multi-copy genes present in all three individuals and 37 total not present in single individuals, likely due to their coverage differences (ten were missing in Sister 1, thirteen in Sister 2 and fourteen in Eureka). Overall, the number of annotated genes was comparable to those found in the domestic dog genome and the wolf genome (Supporting Information Table S4; [34,35]).

Variant rates

We found a high number of heterozygous sites to be shared between all three individuals (321k; here we report the heterozygous sites called using a posterior probability cutoff of 0.99; Supplementary Information Figure S2A). As expected, Sister 1 and Sister 2 share more heterozygous sites (344k) than either sister with Eureka (168k and 170k, for Sister 1 and Sister 2, respectively). Each individual shows a high number of singletons (heterozygous sites only found in one individual), with Sister 2 showing the highest number (1,100k), followed by Sister 1 (968k) and Eureka (825k). Even if we include the two low-coverage genomes from Campana et al. (2016) [21], we find a high number of shared heterozygous sites between all individuals (134k; Supporting Information Figure S2B). We see a higher number of singletons in these two individuals, most likely due to the lower reliability of the genotype calls caused by the low-coverage data (false positives caused by sequencing errors). We estimated a per site heterozygosity of 0.0008 to 0.0012 for Sister 1, 0.0009 to 0.0012 for Sister 2, and 0.0007 to 0.001 for Eureka using posterior cutoffs for genotype calls from 0.95 to 1 in ANGSD (Supporting Information, Fig. S1C). As can be seen in Supplementary Figure S2, except for a posterior probability cutoff of 1, where Sister 1 shows the highest heterozygosity, Sister 2 always shows the highest, Sister 1 the second highest and Eureka the lowest

heterozygosity. Interestingly, Eureka shows a lower heterozygosity than the other two assemblies, even though its parents are thought to have originated from different localities (Supplement S1). With more stringent filtering, we likely could improve the heterozygosity estimates for the low-coverage individuals, but we did not investigate this further and maintained our methods across datasets for comparative purposes.

We did not see any major difference between heterozygosity estimates from repeat-masked and unmasked genomes [66]. The Supernova software estimated a heterozygous position every 2.6kb, 3.1kb, and 7.14kb for Sister 1, Sister2, and Eureka, respectively (Supporting Information Table S5). On the contrary, estimates based on genotype calls using ANGSD showed much more frequent heterozygous positions (850bp - 1.2kb, 814bp - 1.1kb and 999bp - 1.5kb depending on the posterior cutoff used; Supporting Information Table S5). Overall, our estimates show that, while being heavily threatened, African wild dogs seem to still retain a relatively high within-individual heterozygosity relative to other endangered species which have been estimated, such as those in the cheetah or the Amur tiger (> 0.0005, 0.0005; [38]), or the island grey fox (>0.0005; [39]). Additionally, the estimates here are comparable to those from several gray wolf individuals (0.0009-0.0012; [35]).

We also examined the phased data and its effect on heterozygosity estimates for one individual, Sister 2. We find that the estimates are relatively consistent between both the pseudohaplotypes, and the merged pseudohaplotype produced by the Supernova software (Supplementary Information Table S5) [66].

Demographic history

We estimated demographic history using the program PSMC [40]. Our results show similar demographic trends with those reported in Campana et al. (2016) [22], however, we observe declines beginning just over 1mya, as opposed to approximately 700,000 years ago (Figure 1C). From 1 million to 120,000 years ago

the population size steadily declines, resulting in a predicted N_e of approximately 1,000-2,000 individuals. During the remainder of the African wild dog history, there are some small effective population size estimate fluctuations.

We also infer similar population histories from the genomes of the two sisters from Zimbabwe and furthermore, show very little difference between the inferred history of the third individual, Eureka (Figure 1C). This may be because the populations were formerly continuous and share their ancestral population history, but further analyses would be required to disentangle these hypotheses. We also do not detect additional large fluctuations as noted by Campana et al. (2016) [22], but more high coverage genomes from across populations would be needed to confirm that these do not exist, since our individuals are from distinct populations than those previously tested. Furthermore, population structure and short-term demographic incidents (e.g. populations bottlenecks) can affect PSMC estimations of historic population sizes [41]. In addition, the assumed mutation rate and generation times can have large effects on the resulting estimates. However, the data consistently reinforces that African wild dogs have existed at relatively low population sizes for a long time.

Discussion

Assembly continuity and quality

All three African wild dog assemblies produced with 10x Genomics Chromium data showed high continuity, high recovery rates of conserved genes, and expected proportions of repetitive sequence overall. The assembly for Sister 2, which has the highest mean molecule length, is also the most continuous (Contig N50: 83.47kb, Scaffold N50: 21.34Mb; Table 1). Interestingly, the Sister 1 genome has a higher contig N50 (61.34kb) than Eureka (50.15kb), but a lower scaffold N50 (7.91Mb and 15.31Mb, respectively). This may indicate that input molecule length is a key factor

for scaffolding, while coverage is a key factor for contig assembly, and indeed, input DNA quality is noted as the most common cause of failed or substandard assemblies (https://support.10xgenomics.com/de-novo-assembly/software/pipelines/latest/troubleshooting). Furthermore, the percent of the genome able to be phased across genomes did not correspond to input molecule length (Table S1). More work will need to be done to determine the accuracy of the phased data and the wet lab methods and/or assembly parameters which influence these inferences.

Despite having the highest continuity of all three assemblies, Sister 2 did not show the highest BUSCO completeness scores (see Table 2), although the differences were minor (with 95.1% complete BUSCOs compared to 95.4% for Sister 1). Sister 1 achieved the highest BUSCO scores, even compared to the latest domestic dog genome assembly (CanFam3.1 [34]; 95.2%), which has three times higher contig N50 and an almost six times higher scaffold N50. The high scores are remarkable for the limited number of reads used for the assemblies (as low as 25x coverage). As expected, Sister 2, which showed the highest continuity also had the highest repeat content (see Supporting Information Table S3). All three assemblies resulted in similar repeat contents in terms of repeat composition as well as overall percentage (within 3% of each other), with the most continuous assembly (Sister 2) showing the highest number of repeats. Repeat composition in the African wild dog genomes was also similar to the domestic dog and the wolf [34, 35].

All assemblies yielded similar amounts of genes, with Sister 1 showing the highest number (see Supporting Information Table S4), which reflect its BUSCO scores. Closer investigations of one:one and one:many orthologs further showed a very good agreement between annotations obtained from all three individuals. The numbers of annotated genes for all three African wild dogs were similar to those calculated for the latest domestic dog assembly and wolf genome assembly [34, 35].

10x Genomics Chromium system: Feasibility and caveats

Most mammal genomes published in the last several years use a mixture of paired-end (PE) and multiple mate pair (MP) Illumina libraries (e.g. [36] and [42]). While often resulting in good continuity (e.g. [42] or [43]), using different insert libraries considerably increases the cost per genome. On the contrary, 10x Genomics Chromium allows for assembly of a comparable or even more continuous genome using only a single library for a fraction of the cost (see below). Furthermore, as we show here, this library technology generates high-quality assemblies from as low as 25x coverage (see Eureka assembly), while the recommended coverage for PE plus MP assemblies is approximately 80x-100x [44]. We do note however, that the most recent wolf genome used a variety of PE and MP libraries to produce a highly continuous assembly with approximately 30x total coverage [35]. Recently, Mohr and colleagues [37] presented a highly continuous assembly of the endangered Hawaiian monk seal (~2.4Gb total genome assembly length) using a combination of 10x Genomics Chromium and Bionano Genomics optical mapping. Interestingly, their 10x Genomics Chromium (sans additional Bionano) assembly showed similar N50 statistics to those reported here (scaffold N50 22.23Mb), showing that 10x Genomics Chromium technology alone consistently generates highly continuous mammalian genome assemblies.

A limitation of 10x Genomics Chromium technology is the requirement of fresh tissue samples for the isolation of HMW DNA. This can be difficult or impossible to obtain from some endangered species. Fortunately, small amounts of mammalian blood yield sufficient amounts of HMW DNA when properly stored. Additionally, DNA extraction kits such as the Qiagen MagAttract kit can extract sufficient amounts of HMW DNA from as little as 200µl (See Supplementary Information S1 and Supplementary Information Figure S1). For museum samples, or tissues stored for extended periods of time, reference-based mapping might be the

only option to extract long-range genomic information. However, for extant endangered species, especially those with individuals in captivity, 10x Genomics Chromium offers a cost-effective approach to sequence genomes. For species with genome sizes <1Gb and between ~3Gb and 5.8Gb special data processing will need to be applied (see https://support.10xgenomics.com/de-novo-assembly/sample-prep/doc/technical-note-supernova-guidance). In addition, the amplification primers for the 10x Chromium library preparation are designed for GC contents similar to human (~41%), implying that the method might not work as well for genomes that strongly divert from this GC content (e.g. for some invertebrates).

Cost-effectiveness

Sequencing costs are steadily dropping. At the time the sequencing for this project was carried out a lane on the Illumina HiSeqX cost (output of ~120Gb) approximately $1,500 - $2,000 and a 10x Genomics library prep ranged from $450 to $1000, thus allowing the generation of high quality *de novo* genomes for less than $3,000 total (2016-2017). As we have shown, the 10x method only requires a single library to be sequenced to an average coverage of 25x - 75x for comparable results. Furthermore, computational resources required to assemble the genome are very low. The current version of Supernova 1.2 only requires a minimum of 16 CPU cores and 244Gb of memory (for a human genome at 56x coverage; https://www.10xgenomics.com/), and the assembly can be carried out in only few days (depending on the number of available CPU cores). This is about a reduction of five times the memory requirement compared to the first version of Supernova. Additionally, Supernova does not require parameter input or tuning, thus allowing even novices to easily assemble 10x Genomics Chromium based genomes.

For a comparable Illumina assembly, such as the one produced in Gopalakrishnan et al. (2017), the cost would include two paired-end and two mate-

pair libraries plus the sequencing costs [35]. Although paired-end libraries are relatively cheap to produce ($120-$180 USD), mate-pair libraries can be much more expensive depending on their input size ($2000-$3000 for larger insert sizes, or $700-$1000 if non-size selected). In addition, mate-pair libraries require a much larger quantity of starting material compared to the 10x library prep.

Applications in conservation

Traditionally, conservation biologists have obtained a great deal of genetic information from a few microsatellite markers and/or nuclear and mitochondrial loci. The analysis of microsatellite markers can provide a snapshot into contemporary population structure, but this method risks providing incomplete information on selection and migration and can be an unreliable way to identify individuals from degraded low-quality DNA samples (such as scat) due to the stochastic behavior of marker amplification (allelic dropout; [45]; [46] ;[47]). Moreover, microsatellites can be difficult to successfully design and develop, which can quickly increase costs for species that have little to no genetic information available. The ability to rapidly and cost-effectively generate full genomes will allow conservation biologists to bridge this gap and harvest crucial fine-scale population information for population parameters such as inbreeding (e.g. [48]), load of deleterious mutations (e.g. [49]), gene flow (e.g. [50]) and population structure (e.g. [51]). Once a reference genome has been assembled, optional (low-coverage) re-sequencing data from several individuals allows for the typing of genome-wide information such as single-nucleotide polymorphisms (SNPs), potentially neutral microsatellite loci, and other genomic regions of interest. These data can then be used to investigate the aforementioned population parameters, but also further yield insights into adaptive genetic variation and perhaps the adaptive potential of different populations or species.

Heterozygosity within African wild dog individuals

A high number of heterozygous sites were shared between all three individuals in this study, with Sister 1 and Sister 2 sharing more heterozygous sites than either shared with Eureka. Each of the individuals further showed a high number of singletons (heterozygous sites only found in one individual). Even when compared to the two low-coverage genomes from Campana et al. (2016) we find a high number of shared sites [22]. As expected, we see a much higher rate of singletons in these two individuals. Due to the low-coverage (5.7 - 5.8x average coverage) we suspect a higher proportion of the called heterozygous sites to be false positives due to sequencing errors, which could potentially be removed with more stringent filtering. Heterozygosity per site estimates indicate a high within individual diversity. Estimates ranged from 0.0007 - 0.001 for Eureka to 0.0009 - 0.0012 for Sister 2, which are similar to those obtained for lions (0.00074 – 0.00148) and tigers (0.00087 – 0.00104) [52]. Intriguingly, other threatened carnivores, such as the Iberian lynx (*Lynx pardinus*), the cheetah (*Acinonyx jubatus*), and the island fox (*Urocyon littoralis*) show nearly 10-fold lower heterozygosity (0.0001 [51], 0.0002 [38] and 0.000014 - 0.0004 [39], respectively). The high within-individual heterozygosity could be a result of their social structure, as only unrelated individuals come together to form new packs through dispersal. In addition, Hwange National Park is considered to be a part of the most continuous population of African wild dogs, which may explain the high heterozygosity of Sister 1 and Sister 2 [20]. Further sequencing of other populations and additional unrelated individuals will be needed to assess whether the high within-individual heterozygosity is a range-wide phenomenon in African wild dogs.

The Supernova software reports distance between heterozygous site estimates (see Supporting Information Table S1). Interestingly, those estimates were much lower than the ones obtained based on the genotype calls produced with

ANGSD. While Supernova estimated this distance to be 2.6kb in Sister 1, 3.1kb in Sister 2 and 7.1kb in Eureka, the ANGSD based estimates range from 850bp - 1.2kb for Sister 1, 814bp - 1.1kb for Sister 2 and 999bp - 1.5kb for Eureka, depending on the posterior cutoff used. Supernova calculates the distance between heterozygous sites as part of the assembly process. However, when the fasta consensus sequence is called part of the variation can get flattened (see e.g. [33]). This phenomenon is typically seen in regions between megabubbles, which are nominally homozygous, but could in fact have some variation that cannot be phased by Supernova. We also note that heterozygosity values obtained using genotype calls in ANGSD could also be biased, as they are based on the nominal and not the effective coverage. The nominal coverage is the total number of reads that cover a site in the assembly, whereas for the effective coverage only reads from different barcodes are included in the estimation. If individual barcoded regions amplified with different efficiency during the library preparation step, then heterozygosity estimates could be unreliable. However, this should not strongly affect genome-wide heterozygosity estimates, as we expect this issue to be rare.

Potential Implications

We find that the 10x Genomics Chromium system can be used to assemble highly continuous and accurate mammalian genome assemblies for less than $3,000 US dollars per genome (sequenced 2016 and 2017). The method can be easily applied to species of conservation concern for which genomic methods could greatly benefit their management and monitoring programs. For the African wild dog, these genomes will facilitate more reliable and cost-effective conservation efforts through the use of re-sequencing and SNP-typing methods. Compared to other species of conservation concern, the African wild dog has a relatively high heterozygosity.

Using demographic analyses, we also demonstrate that these wild dog populations appear to have been stable at lower effective population sizes for the past hundred thousand years. Additional studies should inquire whether this is consistent for populations across the African continent and evaluate current effective population sizes. More studies are also required to understand how both the social biology and recent precipitous population declines have impacted the population genomic structure of African wild dogs, and how management might use this information for the benefit and longevity of the species.

Methods

Detailed Methods can be found in Supporting Information (S1).

Samples

Blood samples from two individuals belonging to the same pack in Hwange National Park, Zimbabwe were provided by Painted Dog Conservation (CITES Export permit: ZW/0842/2015, ESA import permit: MA66259B-0, Research Council of Zimbabwe permit: 02553). These individuals were presumed to be sisters from direct observation of their litter at the den (here, named Sister 1 and Sister 2). DNA was extracted from samples two weeks after storage at -80ºC. The third sample was provided by the Endangered Wolf Center, Eureka, Missouri from a captive born individual (here named Eureka). DNA was extracted 9 days after the sample was taken (additional information on sample storage can be found in appendix S1). Though the Chromium library preparation does not require large amounts of DNA, the DNA should have a mean molecule length > 200kb (high-molecular weight, or HMW). DNA from all individuals was extracted from blood samples using the QIAGEN MagAttract HMW DNA kit following the provided instructions.

Genome Assembly

We constructed one sequencing library per individual using the 10x Genomics Chromium System with 1.2ng of HMW input DNA. All libraries were then sequenced on the Illumina HiSeqX (Sister 2, Eureka) or HiSeq 4000 (Sister 1) platform. We subsequently assembled the three genomes using the 10x Genomics genome assembler Supernova 1.1.1 [33]; http://support.10xgenomics.com/de-novo-assembly/software/overview/welcome) using default assembly parameters.

Assembly Quality Assessment

We used the Supernova assembler as well as scripts from Assemblathon 2 to determine continuity statistics, such as the scaffold N50 and the total number of scaffolds [53]. We further applied the program BUSCO v2 (BUSCO, RRID:SCR_015008) [54] to assess the presence of nearly universal lineage-specific single-copy orthologous genes in our assemblies using the mammalian gene set from OrthoDB v9 (OrthoDB, RRID:SCR_011980; 4104 genes; available at http://busco.ezlab.org). We compare these results to the high-quality canFam3.1 assembly of the domestic dog ([34]; *Canis familiaris*). The canFam3.1 assembly was built on 7x coverage of Sanger reads and BAC-end sequencing and has a scaffold N50 of 46Mb. We also inferred the number of BUSCO's in the recently published Hawaiian monk seal genome (which was assembled using a combination of 10x Genomics Chromium and Bionano Genomics Irys data) and the two previously published African wild dog genomes (sequenced with basic short read Illumina technology at low coverage and assembled using the domestic dog for reference mapping; [22]).

Repeat Identification and Masking

We next identified repetitive regions in the genomes as another comparative measure of assembly quality and to prepare the genome for annotation. Repeat

annotation was carried out using both homology-based and *ab-initio* prediction approaches. We used the canid RepBase (http://www.girinst.org/repbase/; [56]) repeat database for the homology-based annotation within RepeatMasker (RepeatMasker, RRID:SCR_012954) [55]. We then carried out *ab-initio* repeat finding using RepeatModeler (RepeatModeler, RRID:SCR_015027).

Gene Annotation

Gene annotation for the three assemblies was performed with the genome annotation pipeline Maker3 (MAKER, RRID:SCR_005309) [57], which implements both *ab-initio* prediction and homology-based gene annotation by leveraging previously published protein sequences from dog, mouse, and human.

Orthologous genes between the three African wild dog assemblies, as well as paralogous genes within each individual, were inferred using Proteinortho [58]. Proteinortho applies highly parallelized reciprocal blast searches to establish orthology and paralogy for genes within and between gene annotation files.

Variant rates

In order to estimate within-individual heterozygosity, we output a single pseudohaplotype using the 'style=pseudohap' parameter within Supernova from Sister 2 to represent the reference sequence. Next, we mapped the raw reads from all three individuals to the reference using BWA-MEM [52]. We then converted the resulting SAM files to BAM format using Samtools [53], and sorted and indexed them using Picard (Picard, RRID:SCR_006525; http://broadinstitute.github.io/picard/). Realignment around insertion/deletion (indel) regions and duplicate marking was performed using GATK (GATK , RRID:SCR_001876), and finally, we called heterozygous sites using a probabilistic framework implemented in ANGSD [54, 62, 63]. We tested different posterior probability cutoffs (1, 0.999,0.99, 0.98, and 0.95). To allow for comparison between all individuals, we down-sampled our three

assemblies to 20x mean nominal coverage (total number of reads covering a position, independent of their barcode) for our analyses. Heterozygosity was then simply calculated as the ratio of variable sites to the total number of sites (variable and invariable). Supernova also outputs the distance between heterozygous sites as part of their assembly report. We then used the read data of Campana et al. (2016) [21] and mapped them to our Sister 2 assembly to compare heterozygosity estimates (using the approach outlined above). Next, we estimated the number of shared heterozygous sites between a) our individuals and b) our individuals and the individuals from Campana et al. (2016) [21]. To do so, we used the *gplots* library in R (https://www.r-project.org) to calculate the overlap between the three sets and to display them in a Venn diagram.

Different pseudohaplotypes were obtained through the Supernova software by selecting either the '--style=pseudohap' or '--style=pseudohap2'. The two fasta files produced by 'pseudohap2' were then analyzed as described above.

Demographic history

We filtered each genome for putative X chromosome sequences by first aligning them to the domestic dog X scaffold [34]. Scaffolds showing significant alignment were then further filtered using the program BLAST [65]. The top hit for each alignment was chosen and all scaffolds which aligned with either the mouse, human, pig, domestic dog, or domestic cat X chromosome were removed. This was repeated for each assembly.

We then mapped the raw reads to the subset of scaffolds using BWA-MEM and called the consensus sequence using SAMtools and BCFtools (SAMtools/BCFtools, RRID:SCR_005227) [59, 60]. Population history was reconstructed using PSMC and scaled using a mutations/site/generation rate of 6.0 x 10^{-9} and a generation time of 5 years [40]. This generation time a

mutation/site/generation rate was chosen because it was the average mutation/site/generation rate inferred in Campana et al. (2016) [22].

Availability of supporting data

Genomic and read data is available in the NCBI database under project accession PRJNA488046. Further supporting data can be found in the *GigaScience* repository, GigaDB [66].

Supporting Information

Detailed information on methods, Supernova output, repeat annotation, gene annotation, heterozygosity calculations, and different posterior probability cutoffs are available online. The authors are solely responsible for the content and functionality of these materials. Queries (other than absence of the material) should be directed to the corresponding author.

Competing interests

Author J. Stuelpnagel is a board member of 10x Genomics Inc. Author Ryan W. Taylor is founder of End2End Genomics Inc.

Authors' contributions

Authors JS, CSZ, PB, SP, EA, and DP conceived the project. Authors EM, HM, OM, and RMC contributed samples and insight to the project. RT assembled the genomes. EA and SP performed the genome annotation and downstream analyses. EA, SP, CST, DP, and RT wrote the paper. All authors read and approved the final manuscript.

Acknowledgements

We thank M. Agnew, C. Asa, L. Padilla, and W. Warren for assistance in obtaining the Eureka sample. T. Linderoth, T. Korneliussen, and K. Bi for help with the different heterozygosity calculations. D. Church from 10x Genomics for discussion on how SuperNova performs the heterozygous site calling. We also thank the reviewers for their extremely helpful comments and suggestions in the improvement of this manuscript as well as the editor and GigaDB staff for the assistance in submitting the supporting data and refining the manuscript. This work was funded by a donation to the Program for Conservation Genomics at Stanford University.

Literature Cited

1. Pimm SL, Jenkins CN, Abell R, Brooks TM, Gittleman JL, Joppa LN, Raven PH, Roberts CM. and Sexton JO. The biodiversity of species and their rates of extinction, distribution, and protection. Science. 2014; 344(6187):1246752.
2. Ceballos G, Ehrlich PR, Barnosky AD, García A, Pringle RM, Palmer TM. Accelerated modern human–induced species losses: Entering the sixth mass extinction. Science advances. 2015; 5:e1400253.
3. Epstein B, Jones M, Hamede R, Hendricks S, McCallum H, Murchison EP, Schönfeld B, Wiench C, Hohenlohe P, Storfer A. Rapid evolutionary response to a transmissible cancer in Tasmanian devils. Nature communications. 2016; 7:12684.
4. Harper C, Ludwig A, Clarke A, Makgopela K, Yurchenko A, Guthrie A, Dobrynin P, Tamazian G, Emslie R, van Heerden M, Hofmeyr M. Robust forensic matching of confiscated horns to individual poached African rhinoceros. Current Biology. 2018; 28(1):R13-4.
5. Steiner CC, Putnam AS, Hoeck PE, Ryder OA. Conservation genomics of threatened animal species. Annu. Rev. Anim. Biosci. 2013; 1(1):261-81.

6. Shafer AB, Wolf JB, Alves PC, Bergström L, Bruford MW, Brännström I, Colling G, Dalén L, De Meester L, Ekblom R, Fawcett KD. Genomics and the challenging translation into conservation practice. Trends in Ecology & Evolution. 2015; 30(2):78-87.6.

7. Girman DJ, Kat PW, Mills MG, Ginsberg JR, Borner M, Wilson V, Fanshawe JH, Fitzgibbon C, Lau LM, Wayne RK. Molecular genetic and morphological analyses of the African wild dog (Lycaon pictus). Journal of Heredity. 1993; 84(6):450-9.

8. Woodroffe R, Ginsberg J, and MacDonald DW. The African wild dog: status survey and conservation action plan. IUCN/SSC Canid Specialist Group. 1997; IUCN.

9. IUCN/SSC Regional conservation strategy for the cheetah and African wild dog in Southern Africa. IUCN. Species Survival Commission Gland. 2007; IUCN.

10. Woodroffe R, Sillero-Zubiri C. Lycaon pictus. The IUCN Red List of Threatened Species. 2012;2012:e-T12436A16711116.

11. Woodroffe R and Ginsberg JR. Edge effects and the extinction of populations inside protected areas. Science. 1998; 280(5372):2126-2128.

12. Courchamp F, Clutton-Brock T, and Grenfell B. Inverse density dependence and the Allee effect. Trends in Ecology & Evolution. 1999; 14(10):405-410.

13. Courchamp F, Clutton-Brock T, and Grenfell B. Multipack dynamics and the Allee effect in the African wild dog, Lycaon pictus. Animal Conservation forum. 2000; 3(4):277-285. Cambridge University Press.

14. McNutt JW and Silk JB. Pup production, sex ratios, and survivorship in African wild dogs, Lycaon pictus. Behavioral Ecology and Sociobiology. 2008; 62(7):1061-1067.

15. McNutt JW. Sex-biased dispersal in African wild dogs, Lycaon pictus. Animal behaviour. 1996; 52(6):1067-1077.

16. Fanshawe JH and Fitzgibbon CD. Factors influencing the hunting success of an African wild dog pack. Animal behaviour. 1993; 45(3):479-490.

17. Creel S and Creel NM. Six ecological factors that may limit African wild dogs, Lycaon pictus. Animal Conservation. 1998; 1(1):1-9.

18. Creel S and Creel NM. Opposing effects of group size on reproduction and survival in African wild dogs. Behavioral Ecology. 2015; 26(5):1414-1422.

19. Girman DJ, Vila C, Geffen E, Creel S, Mills MG, McNutt JW, Ginsberg JK, Kat PW, Mamiya KH, Wayne RK. Patterns of population subdivision, gene flow and genetic variability in the African wild dog (Lycaon pictus). Molecular Ecology. 2001; 10(7):1703-23.

20. Marsden CD, Woodroffe R, Mills MG, McNutt JW, Creel S, Groom R, Emmanuel M, Cleaveland S, Kat P, Rasmussen GS, Ginsberg J. Spatial and temporal patterns of neutral and adaptive genetic variation in the endangered African wild dog (Lycaon pictus). Molecular Ecology. 2012; 21(6):1379-93.

21. Marsden CD, Mable BK, Woodroffe R, Rasmussen GS, Cleaveland S, McNutt JW, Emmanuel M, Thomas R, Kennedy LJ. Highly endangered African wild dogs (Lycaon pictus) lack variation at the major histocompatibility complex. Journal of heredity. 2009; 100:S54-65.

22. Campana MG, Parker LD, Hawkins MT, Young HS, Helgen KM, Gunther MS, Woodroffe R, Maldonado JE, Fleischer RC. Genome sequence, population history, and pelage genetics of the endangered African wild dog (Lycaon pictus). BMC genomics. 2016; 17(1):1013.

23. Shapiro B and Hofreiter M. A paleogenomic perspective on evolution and gene function: new insights from ancient DNA. Science. 2014; 343(6169):1236573.

24. Lindblad-Toh K, Wade CM, Mikkelsen TS, Karlsson EK, Jaffe DB, Kamal M, Clamp M, Chang JL, Kulbokas III EJ, Zody MC, Mauceli E. Genome sequence, comparative analysis and haplotype structure of the domestic dog. Nature. 2005; 438(7069):803.

25. Perini FA, Russo CA, Schrago CG. The evolution of South American endemic canids: a history of rapid diversification and morphological parallelism. Journal of evolutionary biology. 2010; 23(2):311-22.

26. Koepfli KP, Pollinger J, Godinho R, Robinson J, Lea A, Hendricks S, Schweizer RM, Thalmann O, Silva P, Fan Z, Yurchenko AA. Genome-wide evidence reveals that African and Eurasian golden jackals are distinct species. Current Biology. 2015; 25(16):2158-65.

27. International Human Genome Sequencing Consortium. Initial sequencing and analysis of the human genome. Nature. 2001; 409(6822):860.

28. Hayden, E.C., The $1,000 genome. Nature, 2014. 507(7492): p. 294. Hayden EC. Is the $1,000 genome for real?. Nature News. 2014 Jan 15.

29. Goodwin S, McPherson JD, McCombie WR. Coming of age: ten years of next-generation sequencing technologies. Nature Reviews Genetics. 2016; 17(6):333.

30. Ekblom R, Wolf JB. A field guide to whole-genome sequencing, assembly and annotation. Evolutionary applications. 2014; 7(9):1026-42.

31. Putnam NH, O'Connell BL, Stites JC, Rice BJ, Blanchette M, Calef R, Troll CJ, Fields A, Hartley PD, Sugnet CW, Haussler D. Chromosome-scale shotgun assembly using an in vitro method for long-range linkage. Genome research. 2016; 26(3):342-50.

32. Burton JN, Adey A, Patwardhan RP, Qiu R, Kitzman JO, Shendure J. Chromosome-scale scaffolding of de novo genome assemblies based on chromatin interactions. Nature biotechnology. 2013; 31(12):1119.

33. Weisenfeld NI, Kumar V, Shah P, Church DM, Jaffe DB. Direct determination of diploid genome sequences. Genome research. 2017; 27(5):757-67.

34. Hoeppner MP, Lundquist A, Pirun M, Meadows JR, Zamani N, Johnson J, Sundström G, Cook A, FitzGerald MG, Swofford R, Mauceli E. An improved canine genome and a comprehensive catalogue of coding genes and non-coding transcripts. PloS one. 2014; 9(3):e91172.

35. Gopalakrishnan S, Castruita JA, Sinding MH, Kuderna LF, Räikkönen J, Petersen B, Sicheritz-Ponten T, Larson G, Orlando L, Marques-Bonet T, Hansen AJ. The wolf reference genome sequence (Canis lupus lupus) and its implications for Canis spp. population genomics. BMC genomics. 2017; 18(1):495.

36. Lok S, Paton TA, Wang Z, Kaur G, Walker S, Yuen RK, Sung WW, Whitney J, Buchanan JA, Trost B, Singh N. De novo genome and transcriptome assembly of the Canadian beaver (Castor canadensis). G3: Genes, Genomes, Genetics. 2017; 7(2):755-73.

37. Mohr DW, Naguib A, Weisenfeld N, Kumar V, Shah P, Church DM, Jaffe D, Scott AF. Improved de novo Genome Assembly: Synthetic long read sequencing combined with optical mapping produce a high quality mammalian genome at relatively low cost. bioRxiv. 2017; 128348.

38. Dobrynin, P., et al., Genomic legacy of the African cheetah, Acinonyx jubatus. Genome biology, 2015. 16(1): p. 277.

39. Robinson JA, Ortega-Del Vecchyo D, Fan Z, Kim BY, Marsden CD, Lohmueller KE, Wayne RK. Genomic flatlining in the endangered island fox. Current Biology. 2016; 26(9):1183.

40. Li H, Durbin R. Inference of human population history from individual whole-genome sequences. Nature. 2011; 475(7357):493.

41. Orozco-terWengel P. The devil is in the details: the effect of population structure on demographic inference. Heredity. 2016 Apr;116(4):349.

42. Liu S, Lorenzen ED, Fumagalli M, Li B, Harris K, Xiong Z, Zhou L, Korneliussen TS, Somel M, Babbitt C, Wray G. Population genomics reveal recent speciation and rapid evolutionary adaptation in polar bears. Cell. 2014; 157(4):785-94.

43. Huang J, Zhao Y, Shiraigol W, Li B, Bai D, Ye W, Daidiikhuu D, Yang L, Jin B, Zhao Q, Gao Y. Analysis of horse genomes provides insight into the diversification and adaptive evolution of karyotype. Scientific reports. 2014; 4:4958.

44. Gnerre S, MacCallum I, Przybylski D, Ribeiro FJ, Burton JN, Walker BJ, Sharpe T, Hall G, Shea TP, Sykes S, Berlin AM. High-quality draft assemblies of mammalian genomes from massively parallel sequence data. Proceedings of the National Academy of Sciences. 2011; 108(4):1513-8.

45. Frantzen MA, Silk JB, Ferguson JW, Wayne RK, Kohn MH. Empirical evaluation of preservation methods for faecal DNA. Molecular Ecology. 1998; 7(10):1423-8.

46. Taberlet P, Luikart G. Non-invasive genetic sampling and individual identification. Biological journal of the linnean society. 1999; 68(1-2):41-55.

47. Morin PA, Luikart G, Wayne RK. SNPs in ecology, evolution and conservation. Trends in Ecology & Evolution. 2004; 19(4):208-16.

48. Vieira FG, Fumagalli M, Albrechtsen A, Nielsen R. Estimating inbreeding coefficients from NGS data: impact on genotype calling and allele frequency estimation. Genome research. 2013; 23(11):1852-61.

49. Pazmiño DA, Maes GE, Simpfendorfer CA, Salinas-de-León P, van Herwerden L. Genome-wide SNPs reveal low effective population size within confined management units of the highly vagile Galapagos shark (Carcharhinus galapagensis). Conservation Genetics. 2017; 18(5):1151-63.

50. Hampton JO, Spencer P, Alpers DL, Twigg LE, Woolnough AP, Doust J, Higgs T, Pluske J. Molecular techniques, wildlife management and the importance of genetic population structure and dispersal: a case study with feral pigs. Journal of Applied Ecology. 2004; 41(4):735-43.

51. Abascal F, Corvelo A, Cruz F, Villanueva-Cañas JL, Vlasova A, Marcet-Houben M, Martínez-Cruz B, Cheng JY, Prieto P, Quesada V, Quilez J. Extreme genomic erosion after recurrent demographic bottlenecks in the highly endangered Iberian lynx. Genome biology. 2016; 17(1):251.

52. Kim S, Cho YS, Kim HM, Chung O, Kim H, Jho S, Seomun H, Kim J, Bang WY, Kim C, An J. Comparison of carnivore, omnivore, and herbivore mammalian genomes with a new leopard assembly. Genome biology. 2016; 17(1):211.

53. Bradnam KR, Fass JN, Alexandrov A, Baranay P, Bechner M, Birol I, Boisvert S, Chapman JA, Chapuis G, Chikhi R, Chitsaz H. Assemblathon 2: evaluating de novo methods of genome assembly in three vertebrate species. GigaScience. 2013; 2(1):10.

54. Simão FA, Waterhouse RM, Ioannidis P, Kriventseva EV, Zdobnov EM. BUSCO: assessing genome assembly and annotation completeness with single-copy orthologs. Bioinformatics. 2015; 31(19):3210-2.

55. Smit AF, Hubley R, Green P. 1996–2010. RepeatMasker Open-3.0.

56. Jurka J, Kapitonov VV, Pavlicek A, Klonowski P, Kohany O, Walichiewicz J. Repbase Update, a database of eukaryotic repetitive elements. Cytogenetic and genome research. 2005; 110:462-7.

57. Holt C, Yandell M. MAKER2: an annotation pipeline and genome-database management tool for second-generation genome projects. BMC bioinformatics. 2011; 12(1):491.

58. Lechner M, Hernandez-Rosales M, Doerr D, Wieseke N, Thévenin A, Stoye J, Hartmann RK, Prohaska SJ, Stadler PF. Orthology detection combining clustering and synteny for very large datasets. PLoS One. 2014; 9(8):e105015.

59. Li H, Durbin R. Fast and accurate short read alignment with Burrows–Wheeler transform. Bioinformatics. 2009;25(14):1754-60.

60. Li H, Handsaker B, Wysoker A, Fennell T, Ruan J, Homer N, Marth G, Abecasis G, Durbin R. The sequence alignment/map format and SAMtools. Bioinformatics. 2009; 25(16):2078-9.

61. Korneliussen TS, Albrechtsen A, Nielsen R. ANGSD: analysis of next generation sequencing data. BMC bioinformatics. 2014; 15(1):356.

62. Nielsen R, Paul JS, Albrechtsen A, Song YS. Genotype and SNP calling from next-generation sequencing data. Nature Reviews Genetics. 2011; 12(6):443.

63. Nielsen R, Korneliussen T, Albrechtsen A, Li Y, Wang J. SNP calling, genotype calling, and sample allele frequency estimation from new-generation sequencing data. PloS one. 2012; 7(7):e37558.

64. Kurtz S, Phillippy A, Delcher AL, Smoot M, Shumway M, Antonescu C, Salzberg SL. Versatile and open software for comparing large genomes. Genome biology. 2004; 5(2):R12.65.

65. Altschul SF, Madden TL, Schäffer AA, Zhang J, Zhang Z, Miller W, Lipman DJ. Gapped BLAST and PSI-BLAST: a new generation of protein database search programs. Nucleic acids research. 1997; 25(17):3389-402.

66. Armstrong E, Taylor RW, Prost S, Blinston P, van der Meer E, Madzikanda H et al. Supporting data for "Entering the era of conservation genomics: Cost-effective assembly of the African wild dog genome using linked reads" GigaScience Database. 2018 http://dx.doi.org/10.5524/100475

Tables

Table 1. Assembly Statistics. Assembly statistics for the three African wild dog genomes reported by the Supernova assembler. Coverage was assessed using SAMtools depth.

		Sister 1	Sister 2	Eureka
Input	Reads (m)	1,200	801.56	427.6
	Average coverage	69	46	25
	Mean molecule size (kb)	19.91	77.03	52.00
Contig	N50 (kb)	61.34	83.47	50.15
	Longest (kb)	524.60	615.40	450.50
	Number (k)	78.62	68.64	108.00
Scaffold	N50 (mb)	7.91	21.34	15.31
	Longest (mb)	43.96	69.63	41.67
	Number (k)	11.78	17.64	25.78
Total size (gb)	Scaffolds >= 10kb	2.27	2.26	2.20
	Scaffolds >= 500bp	2.34	2.40	2.42

Table 2. Conserved Gene Statistics. Results of the BUSCO v2 gene annotation from three African wild dog genome assemblies, canFam3.1, low-coverage wild dog genomes [22], the recently published wolf genome [35] and the Hawaiian monk seal genome [37].

Assembly	Species	Complete	Single copy	Duplicated	Fragmented	Missing	Total searched
Sister 1	*L. pictus*	3914	3875	39	102	88	4104
Sister 2	*L. pictus*	3903	3845	58	107	94	4104
Eureka	*L. pictus*	3829	3789	40	169	106	4104
canFam3.1	*C. familiaris*	3910	3857	53	98	96	4104
Kenya	*L. pictus*	3849	3823	26	136	119	4104
South Africa	*L. pictus*	3892	3867	25	104	108	4104
Wolf	*C. lupus*	3890	3849	41	110	104	4104
Hawaiian monk seal	*Neomonachus schauinslandi*	3881	3833	48	118	105	4104

Figure 1. (A) Pack of African wild dogs. B) Shared heterozygous sites between the three *de novo* assemblies (calculated using a posterior cutoff of 0.99). More of the heterozygous sites are shared between the two sisters than between either sister and Eureka. C) PSMC reconstruction of the individuals' demographic history. Bootstrap replicates are plotted in lighter colors. Time is in years before present.

Figure 1

Click here to access/download;Figure;AWD_figure1_revision.pdf

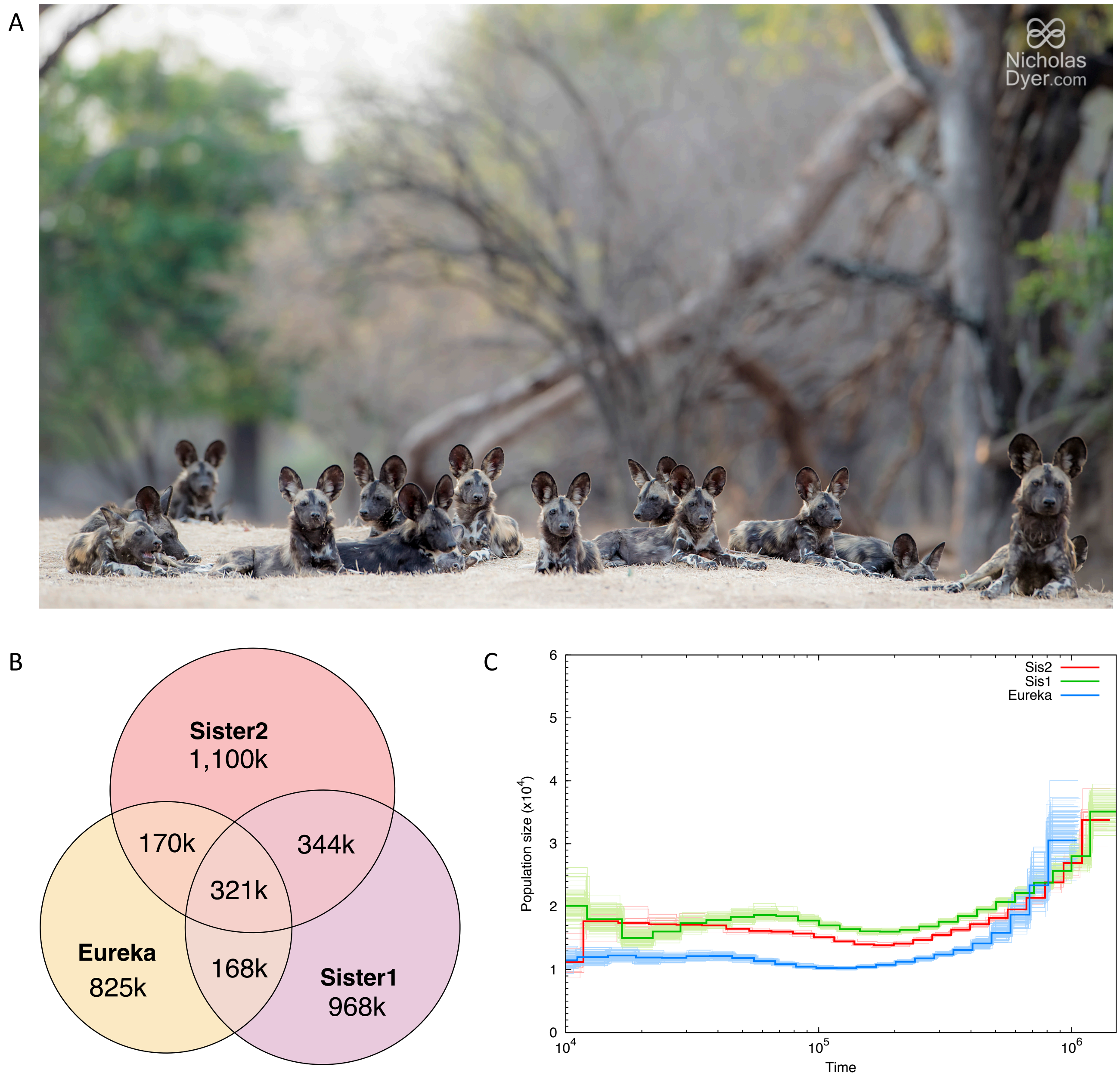

Click here to access/download
Supplementary Material
Supporting_information_AWD_Gigascience_final_update.docx
