# Supplementary material for: Cost-effective assembly of the African wild dog (Lycaon pictus) genome using linked reads
Source: Gigascience. 2018 Oct 22;8(2):giy124. doi: 10.1093/gigascience/giy124 (PMC6350039; doi:10.1093/gigascience/giy124)
Supplement: Supplemental File [file giy124_supplemental_file.docx]

**Supporting Information-Appendix S1**

*Samples*

Blood samples from two individuals belonging to the same pack in Hwange National Park, Zimbabwe were provided by Painted Dog Conservation. These individuals were documented to be sisters (here, named Sister 1 and Sister 2) from direct observation of their litter at the den. They were both born to mother ‘Socks’ in June of 2013 and have unknown paternity. Both samples were collected during routine collaring and health monitoring. From these samples 3ml of blood was aliquoted and frozen immediately in liquid nitrogen and kept frozen in liquid nitrogen for 6 months until transfer to a -80ºC freezer. DNA was extracted two weeks after storage at -80ºC. The third sample was provided by the Endangered Wolf Center, Eureka, Missouri from a captive born individual (here named Eureka). This individual’s mother descended from a male that was wild caught in Ellisras/Lephalale in the Limpopo Province of South Africa, and from a female that was wild caught in Botswana (no further details about location are available). The blood from this sample was treated with EDTA anticoagulant, refrigerated, and shipped on ice. DNA was extracted 9 days after the sample was taken. DNA from all individuals was extracted from blood samples using the QIAGEN MagAttract HMW DNA kit following the provided instructions.

Though the Chromium library preparation does not require large amounts of DNA, the DNA should have a mean molecule length > 200kb (high-molecular weight, or HMW). Unfortunately, we did not have the appropriate equipment or expertise to assess DNA molecule length of greater than approximately 50kb. All samples showed evidence of DNA molecule lengths greater than 50kb. We ran extracted DNA from Sister 2 on a gel using a Pippin Pulse pulsed field gel electrophoresis power supply. The gel shows evidence of fragments >= 50kbp (Figure S1). 10x Genomics also ran extracted DNA from Sister 1 and Sister 2 on an Agilent Tape Station and found a very similar size distribution to human DNA extracted from a cell culture line.


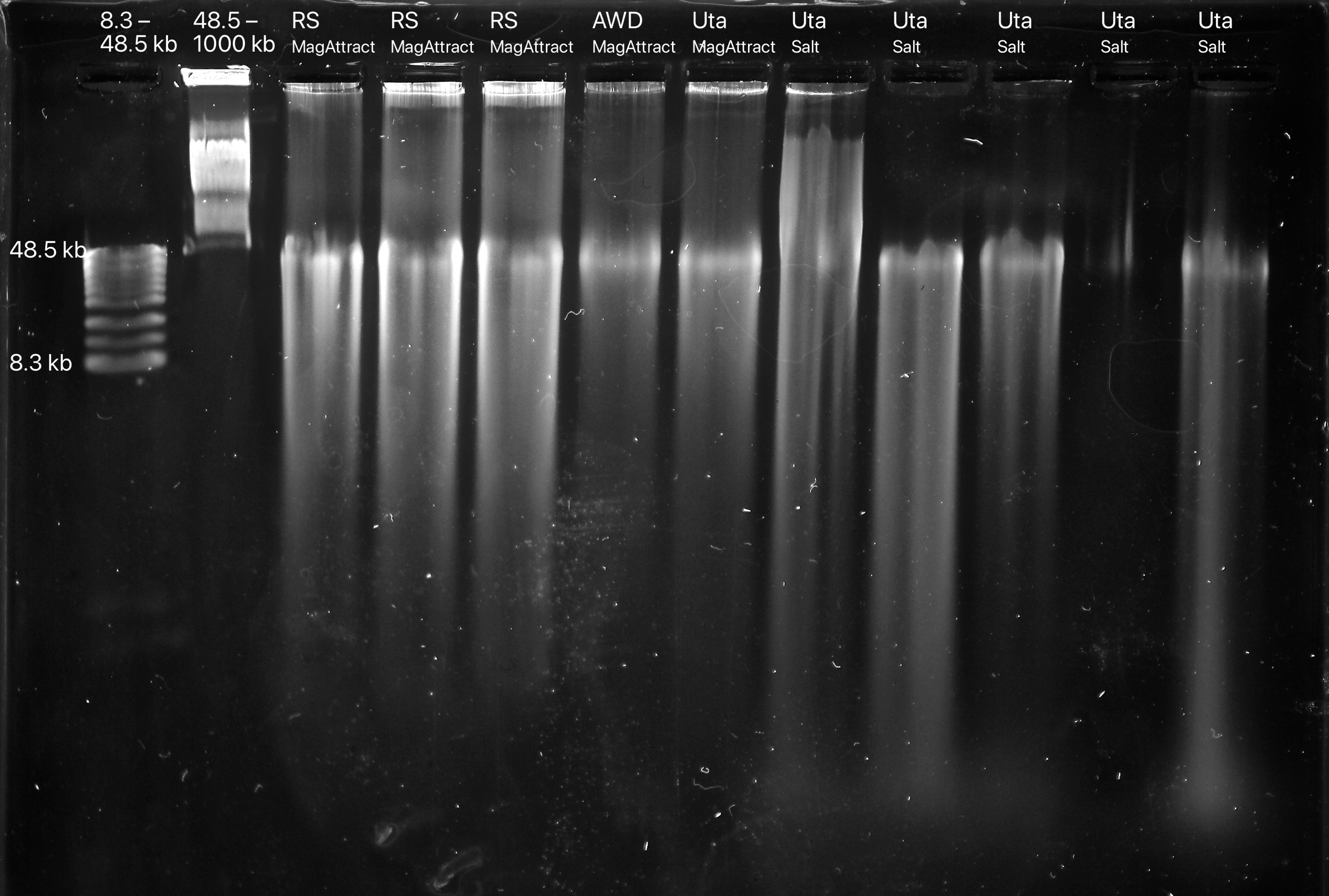


**Figure S1. Pippin Pulse gel image**. Lanes labeled RS and Uta are samples unrelated to this project. AWD is DNA extracted from the blood sample from Sister 2 using the Qiagen MagAttract kit.

*Genome Assembly*

We constructed one sequencing library per individual using the 10x Genomics Chromium System. Each library was constructed using 1.2ng of HMW input DNA. The libraries for Sister 1 and Eureka were prepared and sequenced by 10x Genomics in Pleasanton, California. The library for Sister 2 was prepared and sequenced by HudsonAlpha in Huntsville, Alabama. All libraries were then sequenced on the Illumina HiSeqX (Sister 2, Eureka) or HiSeq 4000 (Sister 1) platform. We generated 1,200 million read pairs for Sister 1, 801.56 million reads for Sister 2, and 427.6 million reads for Eureka.

We subsequently assembled the three genomes using the 10x Genomics genome assembler Supernova 1.1.1 [33]; <http://support.10xgenomics.com/de-novo-assembly/software/overview/welcome>) using default assembly parameters.

*Assembly Quality Assessment*

We used the Supernova assembler as well as the Assemblathon 2 scripts to determine continuity statistics, such as the scaffold N50 and the total number of scaffolds [53]. In order to estimate the N50 statistic, scaffolds first get ranked according to their size. The N50 value is the size of the scaffold when the running sum (starting with the longest scaffold) equals at least half the genome size.  It is similar to the median scaffold length, but puts more weight on longer scaffolds. We further applied the program BUSCO v2 [54] to assess the presence of nearly universal lineage specific single-copy orthologous genes in our assemblies using the mammalian gene set from OrthoDB v9 (4104 genes; available at <http://busco.ezlab.org>). We compare these results to the high-quality canFam3.1 assembly of the domestic dog ([34]; *Canis familiaris*) and the wolf genome (*Canis lupus*) [35]. The canFam3.1 assembly was built on 7x coverage of Sanger reads and BAC-end sequencing and has a scaffold N50 of 46Mb. Prior to long-read technology, this approach was the gold standard to generate high-quality genomes of model organisms. This approach is especially useful for resolving repetitive or complex regions, but unfortunately it is very costly. We also estimated the number of BUSCO’s using the recently published Hawaiian monk seal genome (which was assembled using a combination of 10x Genomics Chromium and Bionano Genomics Irys data and the two previously published African wild dog genomes (sequenced with basic short read Illumina technology at low-coverage and assembled using the domestic dog; [37, 22] ).

*Repeat Identification and Masking*

We next identified repetitive regions in the genomes as another comparative measure of assembly quality and to prepare the genome for annotation. We also performed annotation on the wolf and domestic dog genomes for comparison [34, 35]. Repeat annotation was carried out using both homology-based and *ab-initio* prediction approaches. We used the canid RepBase (<http://www.girinst.org/repbase/>; [56] repeat database for the homology-based annotation within RepeatMasker (<http://www.repeatmasker.org>; [55]. In this step, previously compiled repeats from the canid database were mapped to the genome to identify repeats in the sequence. The RepeatMasker option -gccalc was used to infer GC content for each contig separately to improve the repeat annotation. We then carried out *ab-initio* repeat finding using RepeatModeler (<http://repeatmasker.org/RepeatModeler.html>; [55]). In contrast to RepeatMasker, RepeatModeler does not require previously assembled repeat databases, but identifies repeats in the genome using statistical models.

*Gene Annotation*

Gene annotation for the three assemblies was performed with the genome annotation pipeline Maker3 [57], which implements both *ab-initio* prediction and homology-based gene annotation by leveraging previously published protein sequences from dog, mouse, and human. In order to reduce the number of false positives, we hard-masked tandem elements before running the pipeline.  Hard-masking replaces repeat sequences with Ns and thereby precludes any alignment to these regions. On the other hand, we only soft-masked simple repeats (conversion of sequences to lowercase). This allows alignment to these regions, but prevents the simple repeat from being included in the gene model during the actual gene annotation. We configured Maker3 to soft-mask simple repeats during the pipeline run.

Orthologous genes between the three African wild dog assemblies, as well as paralogous genes within each individual, were inferred using Proteinortho [58]. Proteinortho applies highly parallelized reciprocal blast searches to establish orthology and paralogy for genes within and between gene annotation files.

*Variant rates*

In order to estimate within individual heterozygosity, we used the reference alignment created by the Supernova software using the options ‘mkoutput --style=pseudohap’ from Sister 2 to represent the reference sequence. Next, we mapped the raw reads from all three individuals to the reference using BWA-MEM [59]. We then converted the resulting SAM files to BAM format using SAMtools [60] and sorted and indexed them using Picard (<http://broadinstitute.github.io/picard/>). Realignment around insertion/deletion (indel) regions was performed using GATK, and finally, we called heterozygous sites using a probabilistic framework implemented in ANGSD [61]. We chose a probabilistic over a simple allele counting approach for two reasons. First, a genome coverage of 20x is on the lower side of what is needed to reliably call genotypes [63]. However, even if coverage is as high as 55x, heterozygous sites can be falsely called due to erroneous alignment in low-complexity regions or if reads span areas not covered by the reference genome [60], showing that even high coverage data could benefit from the application of probabilistic genotype calling. Here, we further addressed the former issue by applying realignment around indel regions using GATK. Second, we wanted to use the same approach for all samples, including the low coverage ones from [Campana et al. (2016](#_ENREF_3)) [22]. We tested different posterior probability cutoffs (1, 0.999,0.99 and 0.95) using -doPost 2 -doCutoff 0.95 (with the following filters: -minIndDepth 15 -only_proper_pairs 0 -minQ 20). For the two genomes from [Campana et al. (2016](#_ENREF_3)) we applied -minIndDepth 3 (given their average coverage of 5.7-5.8x). To allow for comparison between all individuals, we down-sampled all individuals to 20x mean nominal coverage (total number of reads covering a position, independent of their barcode) for our analyses. Heterozygosity was then simply calculated as the ratio of variable sites to the total number of sites (variable and invariable). Furthermore, Supernova outputs the distance between heterozygous sites as part of their assembly report. Briefly, here heterozygous sites are called from the assembly graph and are used for phasing (to generate a diploid genome consensus). We further downloaded the read data of [Campana et al. (2016](#_ENREF_3)) and mapped them against our Sister 2 assembly to compare heterozygosity estimates (using the approach outlined above).

To investigate whether the phased data reflected similar heterozygosity estimates, we used the --style=pseudohap2 parameter in Supernova to produce two phased files. We then mapped the read data to these using the methods outlined above and estimated heterozygosity using similar cutoffs.

We then estimated the number of shared heterozygous sites between our individuals. To do so, we used the *gplots* library in R (<https://www.r-project.org>) to calculate the overlap between the three sets and to display them in a Venn diagram. We also integrated the two individuals from Campana et al. (2016) in this analysis. However, it is important to point out that those genotype calls are based on low-coverage data and may not be reliable (see e.g.[62, 63]).

*Demographic History*

In order to assess demographic history, we first filtered putative X chromosomes from the three assemblies. We first aligned our scaffolds to the domestic dog X chromosome obtained from Hoeppner et al. (2014) using the program MUMmerv3 [64]. Specifically, we used nucmer to generate fasta-fasta alignments with a minimum length of alignment of 1000bp (-L 1000). We generated alignment information and filtered for 98 % identity and similarity of the alignments using the show-coords option with parameters -rcl -B -I 98. Those alignments were then filtered using the delta-filter option with parameters -r and -q to generate one to one alignments.

We further filtered these scaffolds by calculating the % alignment of the query sequence (the length of the aligned query from our assembly divided by the total length of the query). Any scaffolds with greater than 1% total alignment were additionally checked using BLAST [65]. All scaffolds which had a top hit with a mammal X chromosome were removed. We then mapped the raw reads back to the genome and called the consensus sequence using SAMtools and BCFtools [59,60].

We then used the program PSMC to infer population history using options -N25 -t15 -r5 -b -p "4+25*2+4+6”. We additionally ran 100 bootstraps. We then plotted this using a mutation rate/site/basepair of 6x10^-9^ and a generation time of 5 years. These parameters were chosen because this was the approximate median mutation rate/site/basepair found in Campana et al. (2016) [22].

Table S1: **Assembly statistics as calculated by Supernova.**

| Statistic | Sister 1 | Sister 2 | Eureka |
| --- | --- | --- | --- |
| Reads | 1200 M | 801.56 M | 427.6 M |
| Mean read length | 138 bp | 139 bp | 138 bp |
| Read two Q30 | 71.56 % | 87.01 % | 80.86 % |
| Median insert | 0.38 Kb | 0.31 Kb | 0.34 Kb |
| Proper pairs | 86.04 % | 89.44 % | 86.81 % |
| Molecule length | 19.91 Kb | 77.03 Kb | 52.0 Kb |
| Heterozygosity distance | 2.61 Kb | 3.11 Kb | 7.14 Kb |
| Number of unbarcoded reads | 5.79 % | 4.77 % | 5.17 % |
| N50 reads per barcode | 972 | 678 | 354 |
| Duplicates | 23.46 % | 21.7 % | 3.28 % |
| Phased | 39.07 % | 40.1 % | 52.54 % |
| Scaffolds >= 10kb | 1.12 K | 1.2 K | 1.56 K |
| N50 edge size | 5.96 Kb | 10.88 Kb | 9.36 Kb |
| N50 contig size | 61.34 Kb | 83.47 Kb | 50.15 Kb |
| N50 phase block size | 0.12 Mb | 2.02 Mb | 0.31 Mb |
| N50 scaffold size | 7.91 Mb | 21.34 Mb | 15.31 Mb |
| N60 scaffold size | 6.2 Mb | 17.04 Mb | 11.49 Mb |
| Assembly size (scaffolds >=10kb) | 2.27 Gb | 2.26 Gb | 2.20 Gb |

Table S2. **Assembly statistics as calculated by Assemblathon 2 scripts.**

|  | Dog | Wolf | Sis1 | Sis2 | Eureka | Kenya wild dog | South Africa wild dog |
| --- | --- | --- | --- | --- | --- | --- | --- |
| Number of scaffolds | 3,268 | 8,747 | 11,784 | 17,641 | 25,783 | 813 | 803 |
| Largest scaffold | 123,869,142 | 12,722,267 | 43,957,506 | 69,643,340 | 41,673,113 | 123,854,990 | 123,855,249 |
| Scaffold N50 | 63,241,923 | 1,584,122 | 7,998,235 | 21,450,969 | 15,073,158 | 64,190,785 | 63,240,551 |
| Number of contigs | 20,229 | 60,703 | 61,669 | 53,205 | 97,072 | 1,219,697 | 660,234 |
| Largest contig | 4,109,767 | 1,128,778 | 644,289 | 901,967 | 606,945 | 89,055 | 85,705 |
| Contig N50 | 411,364 | 89,096 | 84,387 | 124,769 | 57,091 | 3,198 | 6,830 |

Table S3. **Repeat statistics**. *De novo* and homology based repeat annotations as reported by RepeatMasker and RepeatModeler. Families of repeats included here are long interspersed nuclear elements (LINEs), short interspersed nuclear elements (SINEs), long tandem repeats (LTR), DNA repeats (DNA), unclassified (unknown) repeat families, small RNA repeats (SmRNA), and others (consisting of small, but classified repeat groups). The total is the total percentage of base pairs made up of repeats in each genome, respectively.

| Assembly | LINE | SINE | LTR | DNA | Unclassified | SmRNA | Others | Total (%) |
| --- | --- | --- | --- | --- | --- | --- | --- | --- |
| Sister 1 | 847,645 | 1,472,018 | 297,851 | 315,865 | 11,125 | 1,080,087 | 1,054,219 | 40.19 |
| Sister 2 | 857,045 | 1,490,757 | 301,853 | 319,940 | 10,103 | 1,095,228 | 1,050,189 | 41.42 |
| Eureka | 855,470 | 1,479,441 | 299,492 | 317,939 | 12,936 | 1,086,289 | 1,025,874 | 38.65 |
| Wolf | 837,437 | 1,453,070 | 289,436 | 308,534 | 10,829 | 1,061,566 | 1,005,085 | 38.70 |
| canFam3.1 | 857,579 | 1,503,465 | 302,932 | 321,141 | 14,466 | 1,110,467 | 1,038,344 | 42.13 |

Table S4. **Gene Annotation Statistics**. Total number and average gene transcript sizes as reported by Maker3.

| Assembly | Total genes annotated | Average transcript size (bp) |
| --- | --- | --- |
| Sister 1 | 20,946 | 1,289 |
| Sister 2 | 20,649 | 1,287 |
| Eureka | 20,817 | 1,212 |
| Wolf | 21,384 | 1,311 |
| canFam3.1 | 19,634 | 1,339 |

**
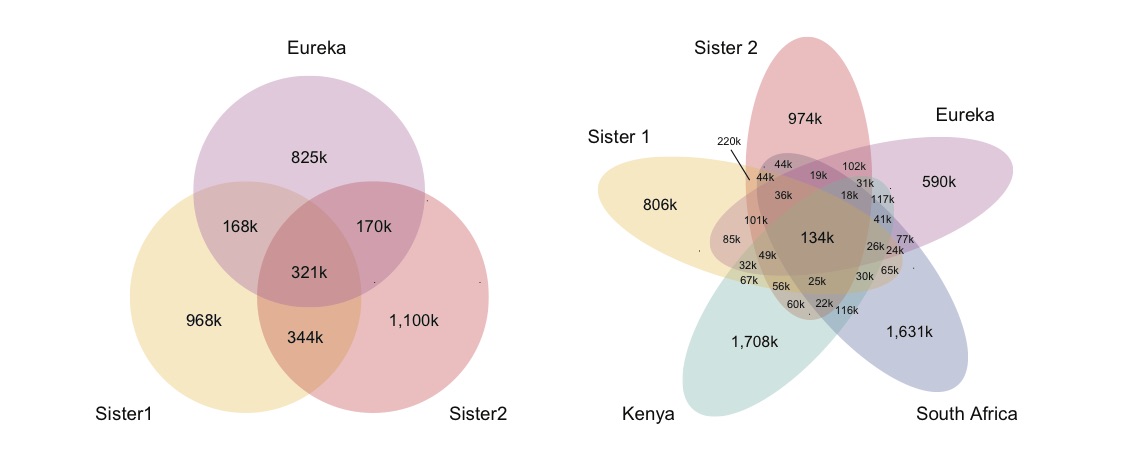
**

Figure S2. **Comparison of heterozygous sites between individuals.** A) Shared heterozygous sites between the three *de novo* assemblies. Many heterozygous sites are shared between all individuals, and more heterozygous sites are shared between the two sisters than between each sister and Eureka. B) Shared heterozygous sites between the three *de novo* assemblies and the two low-coverage reference-based genomes (Kenya and South Africa) from Campana et al. 2016. Both Kenya and South Africa show a very high number of singletons, which is likely caused by the low coverage and the resulting false-positive heterozygous sites (caused by sequencing errors). We see that a high amount of heterozygous sites are shared between all individuals, and that Sister 1 and Sister 2 share more heterozygous sites than any other pairwise comparison.

Table S5. **Heterozygosity estimates.** The total number of sites, the total number of heterozygous sites, the calculated heterozygosity and the length in bp between heterozygous sites is provided for all three genomes for different posterior probability cutoffs.

| Genome assembly | Posterior cutoff | Total number of Sites | Total number of heterozygous sites | Heterozygosity | Length (bp) between heterozygous sites |
| --- | --- | --- | --- | --- | --- |
| Sister 1 | 1 | 940,480,720 | 1,107,829 | 0.0012 | 849 |
|  | 0.999 | 1,863,729,959 | 1,485,163 | 0.0008 | 1,255 |
|  | 0.99 | 1,876,594,049 | 1,801,440 | 0.0010 | 1,042 |
|  | 0.98 | 1,879,378,076 | 1,942,525 | 0.0010 | 967 |
|  | 0.95 | 1,883,014,319 | 2,188,413 | 0.0012 | 860 |
|  |  |  |  |  |  |
| Sister 2 | 1 | 1,063,807,193 | 1,156,011 | 0.0011 | 920 |
|  | 0.999 | 1,820,298,841 | 1,586,917 | 0.0009 | 1147 |
|  | 0.99 | 1,829,356,947 | 1,934,764 | 0.0011 | 946 |
|  | 0.98 | 1,831,414,501 | 2,065,053 | 0.0011 | 887 |
|  | 0.95 | 1,833,781,723 | 2,252,213 | 0.0012 | 814 |
|  |  |  |  |  |  |
| Eureka | 1 | 1,123,979,892 | 1,124,799 | 0.0010 | 999 |
|  | 0.999 | 1,972,583,001 | 1,314,557 | 0.0007 | 1,501 |
|  | 0.99 | 1,983,203,305 | 1,483,893 | 0.0007 | 1,336 |
|  | 0.98 | 1,985,824,623 | 1,551,950 | 0.0008 | 1,280 |
|  | 0.95 | 1,989,156,093 | 1,663,356 | 0.0008 | 1,196 |
|  |  |  |  |  |  |
| Sister 2, Pseudohap 1 | 1 | 812,457,309 | 1,042,311 | 0.0013 | 779 |
|  | 0.999 | 1,643,332,572 | 1,455,771 | 0.0009 | 1,128 |
|  | 0.99 | 1,653,529,033 | 1,800,134 | 0.0011 | 918 |
|  | 0.98 | 1,655,601,261 | 1,926,933 | 0.0012 | 858 |
|  | 0.95 | 1,658,089,788 | 2,108,228 | 0.0013 | 786 |
|  |  |  |  |  |  |
| Sister 2, Pseudohap 2 | 1 | 812,470,548 | 1,041,644 | 0.0013 | 779 |
|  | 0.999 | 1,644,795,927 | 1,455,328 | 0.0009 | 1,129 |
|  | 0.99 | 1,653,534,185 | 1,799,315 | 0.0011 | 918 |
|  | 0.98 | 1,655,603,102 | 1,925,978 | 0.0012 | 859 |
|  | 0.95 | 1,658,090,307 | 2,107,642 | 0.0013 | 786 |


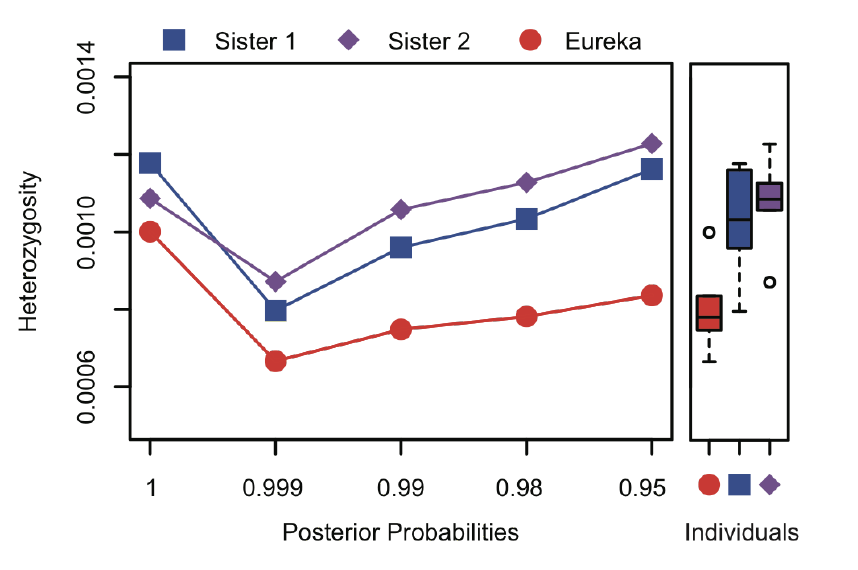


Figure S3 **Distribution of heterozygosity based on different posterior probabilities.** Comparison of heterozygosity estimates using different posterior probability cutoffs for all three assemblies. Boxplot of heterozygosity values (y-axis) calculated for different posterior probability cutoffs.
