# Supplementary material for: Cost-effective assembly of the African wild dog (Lycaon pictus) genome using linked reads
Source: Gigascience. 2018 Oct 22;8(2):giy124. doi: 10.1093/gigascience/giy124 (PMC6350039; doi:10.1093/gigascience/giy124)
Supplement: Response_to_Reviewer_Comments_Original_Submission.pdf [file giy124_response_to_reviewer_comments_original_submission.pdf]

Author's Response To Reviewer Comments

Close

Response to Editor Comments

We have included additional commentary regarding the sample preparation and processing. We have discussed further reasons for possible differences between the assemblies, as well as noted which parameters we are unable to investigate as a result of this study (e.g. the relationship between estimated molecule input length and percent genome phased). We have also changed the title, as requested.

Reviewer 1

Discretionary Revision: Perhaps it would be useful to run a PSMC-type analysis using multiple wild dog genomes to assess trends in historical population sizes in recent times for African wild dogs. This might produce useful results with conservation applications. There are several methods that have come out recently that can do a decent job with estimating population size in recent times.

We have added a PSMC analyses of our three genomes. The results show comparative historical population sizes to those estimated in Campana et al. (2016) (Figure 1). The most notable differences are in the recent population size estimates and the timing of the beginning of the population decline, but are overall consistent.

Edit: Line 444. The word "Heterozygosity" at the end of the paragraph seems out of place.

This sentence has been revised.

Reviewer 2

Line 84 - 'The lineage is the only surviving member of a lineage of wolf-like canids' is I guess true to some degree, but that could be said of other wolf-like canids like the dhole, Ethiopian wolf, African Golden Wolf etc. Perhaps consider rewriting.

This sentence and others have been revised as suggested from this comment, as well as comments from Reviewer 3 to reflect more accurate predictions of the divergence of the African wild dog lineage from other canids. We have included more up to date estimates for this timing.

Line 171 and elsewhere, term 'high quality' is used. I agree that the scaffold size is excellent, but high quality also can refer to long contig sizes (in particular if one wants to study repeats, duplication etc). It would be useful if the authors could undertake a comparison of the contig sizes recovered here to those other genomes of similar SCAFFOLD quality (in particular genomes generated with different methods) so that readers can get a feel for how the contig size varies when using this approach as opposed to much more expensive methods (e.g. deep PacBio sequencing, or mate pair Illumina). Of the top of my head, one comparison in this regard could be to look at the recently published purely Illumina (mate pair) based wolf de novo genome (Gopalakrishnan et al. 2017 BMC Genomics). Unfortunately that genome is not annotated so other comparisons cannot be made (e.g. gene completeness) but simply what I

suggest would be interesting.

We have added an analyses comparing contig and scaffold sizes of our genomes with the wolf genome. We ran analyses on all genomes using the Assemblathon scripts (Table S2) and BUSCO v2 (Table 2). We also annotated the wolf genome for comparison of gene completeness with the same methods as we annotated the African wild dog genomes.

Line 360-361 - perhaps give sequencing price per GB or per 100GB instead of per lane? As many readers may not know the lane output.

We have noted the output of the sequencer and hope this provides a reference to the reader.

Reviewer 3

We especially thank Reviewer 3 for their extensive time and comments to our manuscript. Below we have outlined responses to these comments, as well as clarification on certain aspects of the manuscript.

1. Lines 1-2: The title should be revised because we've already been in the 'era of conservation genomics' for several years now, so this idea is out of date. How about just shortening the title to: "Cost-effective assembly of the African wild dog (Lycaon pictus) genome using linked reads"

Revised as suggested.

2. Line 80: Add a comma after "Taken together" so that the sentence reads: "Taken together, genomic tools are poised…"

Revised as suggested.

3. Line 82: "The African wild dog…" The species is also known by two other common names that are commonly applied to Lycaon pictus - African painted dog and Cape hunting dog. The former is especially used by many researchers and canid conservationists. Therefore, the authors should include these alternative names: "The African wild dog, also known as the African painted dog or Cape hunting dog (Lycaon pictus) is a medium-sized (18-34kg)…"

Revised as suggested.

4. Line 83: "sub Saharan should by hyphenated.

Revised as suggested.

5. Lines 123-125: "The groups containing the African wild dog and the domestic dog…" The authors cite the Nyakatura and Bininda-Emonds (2012) paper on the updated supertree analyses of the Carnivora to support the phylogenetic grouping and divergence time of the African wild dog in relation to the domestic dog. However, the supertree results are inconsistent with more direct assessments of canid phylogenetic history based on analyses of DNA sequences from multiple nuclear and mitochondrial loci.

Supertree analyses have been empirically shown to produce inaccurate results regarding relationships. Direct assessment of DNA sequences indicate that the African wild dog and domestic dog, its wild counterpart, the gray wolf, and other wolf-like canids, are grouped together in the same clade (Tribe Canini, the wolf-like-canids). Furthermore, recent estimates of divergence times suggest that the African wild dog lineage and domestic dog lineage split only about 2.5 - 4 Mya (less than have the age suggested by Nyakatura and Bininda-Emonds, 2012). The authors should instead cite the following references: Lindblad-Toh et al. 2005 Nature 438: 803; Perini et al. 2010 Journal of Evolutionary Biology 23: 311; . The authors should then revise this sentence accordingly.

Associated sentences revised and inferences revised accordingly.

6. Lines 138-139: "...it has been impossible to assemble highly-contiguous genomes from only these short sequences." This statement is incorrect, in particular, the use of the word "impossible." Many mammalian genome assemblies with high continuity (e.g., human, dog, cow, Tasmanian devil, cheetah) have been generated using Illumina short read data. Short read data per se is not the problem. Given that enough paired-end shotgun and mate pair libraries are constructed and sequenced, the resulting short read data can be assembled to produce draft assemblies with high continuity despite the high content of repetitive sequences (comparable to or greater than those generated by the 10X Genomics Chromium System). Therefore, the comparison is a relative one and mostly depends on input. I suggest the authors revise the sentence as follows: "Because large proportions of typical mammal genomes consist of repetitive sequences, it has been challenging to obtain complete or highly continuous genome assemblies using only these short sequences."

Revised as suggested.

7. Lines 173-175: "Thus, in order for it to be useful for conservation purposes the technology needs to be (a) cost-effective and (b) user-friendly." This sentence doesn't make sense and doesn't accord with the facts. Genomes of multiple endangered species (e.g., tiger - Cho et al. 2013 Nat Comm; crested ibis - Li et al 2014 Genome Biol.; cheetah - Dobrynin et al. 2015 Genome Biol.' Iberian lynx - Abascal et al. 2016 Genome Biol.) have been generated and directly useful for conservation purposes regardless of their cost-effectiveness or user-friendliness. The authors' statement precludes other potential sequencing technologies that may not be as cost-effective (e.g. PacBio long reads) but yet still may be used to obtain high quality genome assemblies for conservation genomic applications. And most surprisingly, why should user-friendliness with regards to analysis of next generation sequencing data (i.e., bioinformatics) ever be a criterion on whether it is useful or not for conservation? Please delete this sentence.

We have revised this sentence with an emphasis on the practicality of using genomics as a wide-spread tool in the conservation world. We would defend that it still remains elusive or out of reach for many conservation biologists to assemble a genome de novo, despite desiring to use what a reference assembly provides downstream for everyday conservation practice. We direct the reviewers to a recent study (Taylor et al. (2017) Bridging the conservation genetics gap by identifying barriers to implementation for conservation practitioners. Global Ecology and Conservation), which describes a common disconnect between managers desiring to use genetic and genomic resources, but lacking the funds and expertise to use such technologies.

8. Line 184: "and are presumed to be sisters..." The authors should indicate that the details behind this

presumption are included in the supporting information and cite Appendix S1.

Revised accordingly.

9. Lines 202 - 204: The authors need to cite Hoeppner et al. 2014 here; e.g., "...from the most recent dog genome (267kb and 45.9Mb, respectively [48]),"

Revised accordingly.

10. Line 216: Same comment as point 9; need to cite the Hoeppner et al. 2014 paper.

Revised accordingly.

11. Lines 240-241: "Furthermore, repeat content of all wild dog assemblies was qualitatively similar to canFam3.1." Given that African wild dog and domestic dog share a relatively close evolutionary ancestry (see point #5 above), it's not surprising that their repeat contents would be similar. The authors should qualify their findings in these terms.

Revised accordingly.

12. Lines 242-245: "...the similarity in repeat content between the African wild dog compared to that of the domestic dog, highlights the value of using 10x Genomics Chromium technology to produce accurate and continuous assemblies." This seems like a specious conclusion. The canFam3.1 assembly was not generated using 10x Genomics data, yet it has a repeat content similar to the African wild dogs. This is likely due to the recent common ancestry (point #11) and not because of the technology used to sequence/assemble the genome. The repeat content of the two species would be similar regardless of the continuity of the assembly or how that was achieved. I recommend the authors delete the last sentence in this paragraph.

Revised as suggested.

13. Line 254: "...multi copy..." should be hyphenated (multi-copy).

Revised as suggested.

14. Line 255: "...and 37 not present in one individual." Specify which individual was missing these multi-copy genes (paralogues). Any reason why these 37 multi-copy genes were missing? Lower coverage? Assembly problem?

We re-phrased this sentence to more accurately reflect the results. Thirty-seven total singletons were missing across the three individuals, with the lowest coverage genome missing the most and the highest coverage genome missing the least.

15. Lines 270-272: "As expected, we see a higher number of singletons in these two individuals..." Here the authors should be more explicit about the discrepancy in the number of singleton SNPs in the two African wild dogs sequenced by Campana et al. 2016 and the three individuals sequenced by the authors. Please provide numbers or percentages about the differences and then cite the Appendix S1 for

the detailed methods used for variant calling. Coverage in and of itself may not be the sole reason for the higher number of singletons in the two African wild dogs sequenced by Campana et al. More stringent filtering methods applied to these two individuals would likely have resulted in a comparable number of SNPs to the three individuals sequenced by the authors. The authors should discuss these alternatives. Also, the Nielsen et al. 2011 and 2012 references are not included in the references (main text or Appendix S1). Also, the authors should consider the following papers: Bryc et al. 2013 Genetics 195: 553 and Kousathanas et al. 2017 Genetics 205: 317.

We agree with the reviewer that there is much to be said for the different ways to estimate heterozygosity, but would add that this is difficult to do without introducing additional biases. Indeed, data-preprocessing, the choice of a reference genome (this particular issue is documented in Gopalakrishnan et al. 2017 using the wolf data), mapping tools, and filtering, may all introduce unknown biases in heterozygosity estimates. Our intention in this paper was not to estimate heterozygosity using multiple different methods, but rather use a single method and estimate differences. However, this would be a pertinent follow-up study in the future using a more controlled data set and we will certainly consider this. We have adjusted the language here to acknowledge the limitations of our analyses.

16. Lines 280-281: "Our estimates show that, while being heavily threatened, African Wild dogs seem to still retain a relatively high within individual heterozygosity." First "Wild" in this sentence should be revised as "wild." Second, the conclusion of "relatively high within individual heterozygosity" is impossible to judge without context to some reference/metric or other species. Relative to what exactly? The per site heterozygosities measured by the authors should be compared to those obtained from other species listed as endangered or critically endangered on the IUCN Red List. The paper by Robinson et al. 2016 Current Biol. 26: 1183 would be of use for this. Furthermore, it would be useful to compare the per site heterozygosities obtained for the three African wild dogs with those of gray wolves reported by Gopalakrishnan et al. 2017 BMC Genomics 18: 495 (see their Table S1).

We have included comparisons to those reported for several endangered species in Dobrynin et al. 2016, Gopalakrishnan et al. 2017, and Robinson et al. 2016.

17. Lines 299-301: "This may indicate that input molecule length is a key factor for scaffolding, while coverage is a key factor for contig assembly." Input molecule length is indeed likely to have a strong effect on assembly quality for the 10X Genomics platform. In fact, this is directly stated by 10X Genomics: "DNA quality. By far the most common cause of subpar assembly results is poor input DNA quality" (https://support.10xgenomics.com/de-novo-assembly/software/pipelines/latest/troubleshooting). In fact, the Chromium library preparation process may nick the DNA and thus cause fragmentation (smaller molecule lengths). The authors should include and cite the weblink above. It is somewhat surprising that the assemblies of the three African wild dogs were so different in terms of their assembly metrics (e.g., contig and scaffold N50s). Given that the 10X Genomics linked-read technology is still relatively new, it's difficult to judge whether these results are common or not. The authors' findings do not accord with my own experience using 10X, where assembly metrics from multiple individuals of the same species were more consistent (mostly identical). The authors should discuss in in one or two additional sentences other factors that may have influenced their results: 1) sample handling, storage, and/or preparation; 2) library preparation - were the three libraries prepared by the same lab or technician? The authors state in Appendix S1 that the three individuals were sequenced at two different sequencing facilities/vendors; 3) sequencing platforms, chemistries used (HiSeq X for two individuals vs. HiSeq4000 for the third).

We have included the link as part of our revisions and added this as a commentary. We do emphasize that the three assemblies were sequenced at different depths, which may also result in some of the stochasticity among our assemblies. We hope that what comes across is not that the assemblies are wildly different, but rather that as an assembly service which is cost-effective, that the results across individuals are more or less consistent.

18. Lines 357-375: Cost effectiveness: The authors should list the US sequencing facilities examined and their corresponding prices for Chromium library preparation and sequencing in the Supporting Information- Appendix 1 in a table. This will provide readers with the explicit information to gauge different costs associated with these services. This information is also usually provided on the websites of sequencing facilities and vendors. Also, the authors should indicate the pricings for the library preparation and sequencing at the two sequencing facilities they used to generate the data of the three African wild dogs. Also, how much would the cost be for if the authors had used generated and sequenced Illumina shotgun and mate pair libraries to obtain genome assemblies comparable in quality to those generated using the 10X Chromium platform?

We have included details on the prices we paid for each assembly. We are reluctant to include a survey of current costs because the cost for sequence services changes rapidly, and the prices posted on websites are not always representative of negotiated prices. We believe the prices we paid are within 15% of prices currently offered by most sequencing providers.

We have more explicitly listed the cost of each of our genomes by their components (the price of a lane and the price of the library prep) in comparison with the approximate cost to prepare the libraries and sequencing of the wolf genome, a comparable Illumina library based genome.

19. Lines 408-411: See my previous comments with respect to this issue in point # 15 above. It would be useful to cite Nielsen et al. 2011 and 2012 here.

We have incorporated the Nielsen et al. 2011 & 2012 citations where appropriate. We thank the reviewer for bringing this oversight to our attention.

20. Line 414: "other threatened large bodied carnivores…" - Neither the Iberian lynx nor dwarf Channel island fox would be considered large-bodied. I suggest the authors revise this just as: "other threatened carnivores…"

Revised as suggested.

21. Line 421: a comma should be added after "dogs" in this sentence.

Revised as suggested.

22. Line 433: "…as part of the assembly process, however, when the fasta consensus sequence…" This is a run-on sentence and should be broken into two sentences: "…as part of the assembly process. However, when the fasta consensus sequence…"

Revised as suggested.

23. Line 473: "DNA was extracted 9 days after the sample was taken." The authors should provide details about how this sample was stored prior to DNA extraction. Also, what type of blood tubes (e.g., Vacutainer) were the samples collected into? These details are important to document given the importance of the HMW input DNA to the success of the 10X Genomics Chromium technology (and in the interests of reproducibility).

We had described the storage and processing of the samples in detail, but failed to reference appendix S1. We have corrected this error.

24. Line 486 (and in Appendix S1): In the interests of reproducibility, the default assembly parameters should be listed or described.

There are no assembly parameters for Supernova and it is simply 'supernova run' in the same directory as the fastq files.

25. Line 492: "lineage specific" should include a hyphen.

Revised as suggested.

26. Line 496: "BAC end" should include a hyphen.

Revised as suggested.

27. Lines 524-527: The 10X Genomics Supernova assembler outputs four FASTA data files (raw, megabubbles, pseudohap and pseudohap2); see: https://support.10xgenomics.com/de-novo-assembly/software/pipelines/latest/output/generating. Given that there are only two outputs that provide the phased information (pseudohap and pseudohap2), how could this choice for estimating heterozygosity possibly be described a random? In the interests of reproducibility, the authors should indicate which pseudo-haplotype file was used for which individual African wild dog. Also, the authors should at least take one individual (Sister 2, the one with the most continuous assembly) and estimate the heterozygosity from the other pseudo-haplotype file to check that there is no difference in the inferred number of heterozygous sites (this acts as a control).

We have included an analysis of the two distinct pseudohaplotypes from the --style=pseudohap2 output for Sister 2 and have included a more thorough description of which files were used for each. We do note, however, that the software does a randomized pseudohaplotype when the option --style=pseudohap is chosen and is noted here in the Supernova manual: "For pseudohap...Megabubble arms are chosen arbitrarily so many records will mix maternal and paternal alleles." However, for --style=pseudohap2, the maternal and paternal arms are separated. We have made efforts to make this more clear in the text.

28. Line 529: The Samtools and Picard programs should be capitalized.

Revised as suggested.

29. Literature cited: The authors should carefully check the formatting of their references so that they

consistently conform to the journal standards (e.g., journal titles are often not properly capitalized).

Revised as suggested.

30. Methods (main text and Appendix S1): Samples. Given the requirement of input DNA with long molecule lengths and its importance to the 10X Genomics technology, no details or information is provided on how the HMW genomic DNA was assayed following extraction. This is absolutely crucial and related to the issue of experimental reproducibility. Such HMW DNA is usually assessed using pulse-field electrophoresis techniques or variations thereof. Since the authors used two different sequencing facilities to generate the libraries and sequencing data, different methods may have been used for the assays. In any case, the authors should provide the details about how the HMW DNA was assessed and evaluated prior to Chromium library preparation.

We have added additional information on the assays performed following extraction in the supplement.

31. Phased assemblies: Even though the percentage of the assemblies that were phased is presented in Table S1, this feature is never discussed in detail in the main text. However, this is one of the most noteworthy (and marketed) features of the 10X Genomics platform. Phased assemblies also have a dramatic impact on the downstream population genetic analyses and provide additional information for these analyses compared to technologies that do not yield phased assemblies. The authors should include a description of the phasing results of the three African wild dog assemblies in the Data Description & Analyses section as well as discuss this important feature of the 10X Genomics platform.

We considered this point extensively during analyses, but unfortunately are not able to address this point with the data in hand. Although we can produce phased vcf files, the genomes produced from the Sister 1 and Sister 2 individuals by independent Supernova runs are still too fragmented for us to consider the phasing of any certain haplotype or position, nor to investigate whether the sisters share the expected amount of variation. We are continuing this project with population-level sequencing of individuals from Zimbabwe and hope to address this point further when we have additional information on the expected allele frequencies.

Close
