# Supplementary material for: Cost-effective assembly of the African wild dog (Lycaon pictus) genome using linked reads
Source: Gigascience. 2018 Oct 22;8(2):giy124. doi: 10.1093/gigascience/giy124 (PMC6350039; doi:10.1093/gigascience/giy124)
Supplement: Reviewer_1_Report_(Original_Submission) -- Andreas Chavez — 1/31/2018 Reviewed [file giy124_reviewer_1_report_(original_submission).pdf]

Reviewer Report

Title: Cost-effective assembly of the African wild dog genome using linked reads.

Version: Original Submission **Date:** 1/31/2018

Reviewer name: Andreas Chavez

Reviewer Comments to Author:

Manuscript Number: GIGA-D-17-00324
Title: Entering the era of conservation genomics: Cost-effective assembly of the African wild dog genome using linked reads.

Summary: The study reports a de novo genome assembly for the African Wild Dog and demonstrates a cost-effective approach for generating high-quality assemblies. Genome assemblies have been prohibitively expensive and labor-intensive for researchers focused on non-model organisms, particularly organisms with large genomes. This can be problematic for conservation geneticists that are trying to understand key genetic aspects of endangered taxa, such as their demographic history, inbreeding, and adaptive genetic variation, to name a few. This study demonstrated that high-quality reference genomes using 10X Genomics libraries and standard Illumina sequencing can be generated for around $3,000 for an organism with a relatively large genomes (~2.5GB).

Major Essential Revision: I have not identified any major revisions that are needed for this manuscript.

Discretionary Revision: Perhaps it would be useful to run a PSMC-type analysis using multiple wild dog genomes to assess trends in historical population sizes in recent times for African wild dogs. This might produce useful results with conservation applications. There are several methods that have come out recently that can do a decent job with estimating population size in recent times.

Schiffels, S. and Durbin, R., 2014. Inferring human population size and separation history from multiple genome sequences. Nature genetics, 46(8), p.919.

Paul, J.S., Steinrücken, M. & Song, Y.S. An accurate sequentially Markov conditional sampling distribution for the coalescent with recombination. Genetics 187, 1115-1128 (2011).

Sheehan, S., Harris, K. & Song, Y.S. Estimating variable effective population sizes from multiple genomes: a sequentially Markov conditional sampling distribution approach. Genetics

194, 647-662 (2013).

Steinrücken, M., Paul, J.S. & Song, Y.S. A sequentially Markov conditional sampling distribution for structured populations with migration and recombination. Theor. Popul. Biol. 87, 51-61 (2013).

Edit: Line 444. The word "Heterozygosity" at the end of the paragraph seems out of place.

Recommendations: This study will be very valuable for researchers studying non-model organisms who are interested cost-effective approaches for genome assembly. The manuscript is also very well written and organized.

Methods

Are the methods appropriate to the aims of the study, are they well described, and are necessary controls included? Yes

Conclusions

Are the conclusions adequately supported by the data shown? Yes

Reporting Standards

Does the manuscript adhere to the journal's guidelines on minimum standards of reporting? Yes

Choose an item.

Statistics

Are you able to assess all statistics in the manuscript, including the appropriateness of statistical tests used? Yes, and I have assessed the statistics in my report.

Quality of Written English

Please indicate the quality of language in the manuscript: Acceptable

Declaration of Competing Interests

Please complete a declaration of competing interests, considering the following questions:

- Have you in the past five years received reimbursements, fees, funding, or salary from an organisation that may in any way gain or lose financially from the publication of this manuscript, either now or in the future?
- Do you hold any stocks or shares in an organisation that may in any way gain or lose financially from the publication of this manuscript, either now or in the future?
- Do you hold or are you currently applying for any patents relating to the content of the manuscript?
- Have you received reimbursements, fees, funding, or salary from an organization that holds or has applied for patents relating to the content of the manuscript?
- Do you have any other financial competing interests?
- Do you have any non-financial competing interests in relation to this paper?

If you can answer no to all of the above, write 'I declare that I have no competing interests' below. If your reply is yes to any, please give details below.

I declare that I have no competing interests

I agree to the open peer review policy of the journal. I understand that my name will be included on my report to the authors and, if the manuscript is accepted for publication, my named report including any attachments I upload will be posted on the website along with the authors' responses. I agree for my report to be made available under an Open Access Creative Commons CC-BY license (http://creativecommons.org/licenses/by/4.0/). I understand that any comments which I do not wish to be included in my named report can be included as confidential comments to the editors, which will not be published.

I agree to the open peer review policy of the journal

To further support our reviewers, we have joined with Publons, where you can gain additional credit to further highlight your hard work (see: https://publons.com/journal/530/gigascience). On publication of this paper, your review will be automatically added to Publons, you can then choose whether or not to claim your Publons credit. I understand this statement.

Yes Choose an item.
