# Supplementary material for: Cost-effective assembly of the African wild dog (Lycaon pictus) genome using linked reads
Source: Gigascience. 2018 Oct 22;8(2):giy124. doi: 10.1093/gigascience/giy124 (PMC6350039; doi:10.1093/gigascience/giy124)
Supplement: Reviewer_2_Report_(Original_Submission) -- M. Thomas P. Gilbert — 2/5/2018 Reviewed [file giy124_reviewer_2_report_(original_submission).pdf]

Reviewer Report

Title: Cost-effective assembly of the African wild dog genome using linked reads.

Version: Original Submission **Date:** 2/5/2018

Reviewer name: M. Thomas P. Gilbert

Reviewer Comments to Author:

This is a very nice demonstration of the power of 10x genomics, and I applaud the readers for having explored the quality of the genomes so nicely. I imagine many readers will find this of great interest. I have only a few very small suggestions.

Line 84 - 'The lineage is the only surviving member of a lineage of wolf-like canids' is I guess true to some degree, but that could be said of other wolf-like canids like the dhole, Ethiopian wolf, African Golden Wolf etc. Perhaps consider rewriting.

Line 171 and elsewhere, term 'high quality' is used. I agree that the scaffold size is excellent, but high quality also can refer to long contig sizes (in particular if one wants to study repeats, duplication etc). It would be useful if the authors could undertake a comparison of the contig sizes recovered here to those other genomes of similar SCAFFOLD quality (in particular genomes generated with different methods) so that readers can get a feel for how the contig size varies when using this approach as opposed to much more expensive methods (e.g. deep PacBio sequencing, or mate pair Illumina). Of the top of my head, one comparison in this regard could be to look at the recently published purely Illumina (mate pair) based wolf de novo genome (Gopalakrishnan et al. 2017 BMC Genomics). Unfortunately that genome is not annotated so other comparisons cannot be made (e.g. gene completeness) but simply what I suggest would be interesting.

Line 360-361 - perhaps give sequencing price per GB or per 100GB instead of per lane? As many readers may not know the lane output.

Methods

Are the methods appropriate to the aims of the study, are they well described, and are necessary controls included? Yes

Conclusions

Are the conclusions adequately supported by the data shown? Yes

Reporting Standards

Does the manuscript adhere to the journal's guidelines on minimum standards of reporting? Yes

Choose an item.

Statistics

Are you able to assess all statistics in the manuscript, including the appropriateness of statistical tests used? No, and I do not feel adequately qualified to assess the statistics.

Quality of Written English

Please indicate the quality of language in the manuscript: Acceptable

Declaration of Competing Interests

Please complete a declaration of competing interests, considering the following questions:

- Have you in the past five years received reimbursements, fees, funding, or salary from an organisation that may in any way gain or lose financially from the publication of this manuscript, either now or in the future?
- Do you hold any stocks or shares in an organisation that may in any way gain or lose financially from the publication of this manuscript, either now or in the future?
- Do you hold or are you currently applying for any patents relating to the content of the manuscript?
- Have you received reimbursements, fees, funding, or salary from an organization that holds or has applied for patents relating to the content of the manuscript?
- Do you have any other financial competing interests?
- Do you have any non-financial competing interests in relation to this paper?

If you can answer no to all of the above, write 'I declare that I have no competing interests' below. If your reply is yes to any, please give details below.

I declare that I have no competing interests.

I agree to the open peer review policy of the journal. I understand that my name will be included on my report to the authors and, if the manuscript is accepted for publication, my named report including any attachments I upload will be posted on the website along with the authors' responses. I agree for my report to be made available under an Open Access Creative Commons CC-BY license (http://creativecommons.org/licenses/by/4.0/). I understand that any comments which I do not wish to be included in my named report can be included as confidential comments to the editors, which will not be published.

I agree to the open peer review policy of the journal

To further support our reviewers, we have joined with Publons, where you can gain additional credit to further highlight your hard work (see: https://publons.com/journal/530/gigascience). On publication of this paper, your review will be automatically added to Publons, you can then choose whether or not to claim your Publons credit. I understand this statement.

Yes Choose an item.
