# Supplementary material for: Cost-effective assembly of the African wild dog (Lycaon pictus) genome using linked reads
Source: Gigascience. 2018 Oct 22;8(2):giy124. doi: 10.1093/gigascience/giy124 (PMC6350039; doi:10.1093/gigascience/giy124)
Supplement: Reviewer_3_Report_(Original_Submission) -- Klaus-Peter Koepfli, PhD — 2/6/2018 Reviewed [file giy124_reviewer_3_report_(original_submission).pdf]

Reviewer Report

Title: **Cost-effective assembly of the African wild dog genome using linked reads.**

Version: **Original Submission** **Date:** 2/6/2018

Reviewer name: **Klaus-Peter Koepfli, PhD**

Reviewer Comments to Author:

Review of "Entering the era of conservation genomics: Cost-effective assembly of the African wild dog genome using linked reads" by Ellie E Armstrong et al.

Manuscript number: GIGA-D-17-00324

Armstrong and colleagues report the first de novo genome assemblies from the African wild dog (Lycaon pictus) an endangered African canid with a life history that includes obligate cooperative breeding and hunting in packs. Genomes were sequenced and assembled from three individuals using the 10X Genomics Chromium system and the 10X Genomics Supernova assembler, respectively. The assemblies were evaluated with regards to continuity and quality, and then annotated with regards to protein-coding genes, repetitive element content and genome-wide diversity (heterozygosity). The metrics obtained from these analyses were compared to those from two previously published low coverage Lycaon genomes and the most recent domestic dog genome assembly. The authors suggest that the 10X Genomics Chromium method provides an economical method for generating high quality genomes relative to traditional short read sequencing approaches and that the genomes provide an important resource to enhance conservation management of this species.
The manuscript is generally well written and organized, and therefore the readability of the manuscript is high. The length of the manuscript is satisfactory and the amount of text devoted to the background, results and discussion sections is well balanced. The cited literature is up to date and fully representative of African wild dog conservation genetics and the methods employed. The experimental and analytical methods are technically sound for the most part. There are some major issues that need to be addressed. Below I provide comments about these and additional issues as well as edits that I think would improve the manuscript.

1. Lines 1-2: The title should be revised because we've already been in the 'era of conservation genomics' for several years now, so this idea is out of date. How about just shortening the title to: "Cost-effective assembly of the African wild dog (Lycaon pictus) genome using linked reads"

2. Line 80: Add a comma after "Taken together" so that the sentence reads: "Taken together, genomic tools are poised..."

3. Line 82: "The African wild dog..." The species is also known by two other common names that are commonly applied to Lycaon pictus - African painted dog and Cape hunting dog. The former is especially used by many researchers and canid conservationists. Therefore, the authors should include these alternative names: "The African wild dog, also known as the African painted dog or Cape hunting dog (Lycaon pictus) is a medium-sized (18-34kg)..."

4. Line 83: "sub Saharan should by hyphenated.

5. Lines 123-125: "The groups containing the African wild dog and the domestic dog..." The authors cite the Nyakatura and Bininda-Emonds (2012) paper on the updated supertree analyses of the Carnivora to support the phylogenetic grouping and divergence time of the African wild dog in relation to the domestic dog.

However, the supertree results are inconsistent with more direct assessments of canid phylogenetic history based on analyses of DNA sequences from multiple nuclear and mitochondrial loci. Supertree analyses have been empirically shown to produce inaccurate results regarding relationships. Direct assessment of DNA sequences indicate that the African wild dog and domestic dog, its wild counterpart, the gray wolf, and other wolf-like canids, are grouped together in the same clade (Tribe Canini, the wolf-like-canids). Furthermore, recent estimates of divergence times suggest that the African wild dog lineage and domestic dog lineage split only about 2.5 - 4 Mya (less than have the age suggested by Nyakatura and Bininda-Emonds, 2012). The authors should instead cite the following references: Lindblad-Toh et al. 2005 Nature 438: 803; Perini et al. 2010 Journal of Evolutionary Biology 23: 311; Koepfli et al. 2015 Current Biology 25: 2158. The authors should then revise this sentence accordingly.

6. Lines 138-139: "...it has been impossible to assemble highly-contiguous genomes from only these short sequences." This statement is incorrect, in particular, the use of the word "impossible." Many mammalian genome assemblies with high continuity (e.g., human, dog, cow, Tasmanian devil, cheetah) have been generated using Illumina short read data. Short read data per se is not the problem. Given that enough paired-end shotgun and mate pair libraries are constructed and sequenced, the resulting short read data can be assembled to produce draft assemblies with high continuity despite the high content of repetitive sequences (comparable to or greater than those generated by the 10X Genomics Chromium System). Therefore, the comparison is a relative one and mostly depends on input. I suggest the authors revise the sentence as follows: "Because large proportions of typical mammal genomes consist of repetitive sequences, it has been challenging to obtain complete or highly continuous genome assemblies
using only these short sequences."

7. Lines 173-175: "Thus, in order for it to be useful for conservation purposes the technology needs to be (a) cost-effective and (b) user-friendly." This sentence doesn't make sense and doesn't accord with the facts. Genomes of multiple endangered species (e.g., tiger - Cho et al. 2013 Nat Comm; crested ibis - Li et al 2014 Genome Biol.; cheetah - Dobrynin et al. 2015 Genome Biol.' Iberian lynx - Abascal et al. 2016 Genome Biol.) have been generated and directly useful for conservation purposes regardless of their cost-effectiveness or user-friendliness. The authors' statement precludes other potential sequencing technologies that may not be as cost-effective (e.g. PacBio long reads) but yet still may be used to obtain high quality genome assemblies for conservation genomic applications. And most surprisingly, why should user-friendliness with regards to analysis of next generation sequencing data (i.e., bioinformatics) ever be a criterion on whether it is useful or not for conservation? Please delete this sentence.

8. Line 184: "and are presumed to be sisters..." The authors should indicate that the details behind this presumption are included in the supporting information and cite Appendix S1.

9. Lines 202 - 204: The authors need to cite Hoeppner et al. 2014 here; e.g., "...from the most recent dog genome (267kb and 45.9Mb, respectively [48]),"

10. Line 216: Same comment as point 9; need to cite the Hoeppner et al. 2014 paper.

11. Lines 240-241: "Furthermore, repeat content of all wild dog assemblies was qualitatively similar to canFam3.1." Given that African wild dog and domestic dog share a relatively close evolutionary ancestry (see point #5 above), it's not surprising that their repeat contents would be similar. The authors should qualify their findings in these terms.

12. Lines 242-245: "...the similarity in repeat content between the African wild dog compared to that of the domestic dog, highlights the value of using 10x Genomics Chromium technology to produce accurate and continuous assemblies." This seems like a specious conclusion. The canFam3.1 assembly was not generated using 10x Genomics data, yet it has a repeat content similar to the African wild dogs. This is likely due to the recent common ancestry (point #11) and not because of the technology used to sequence/assemble the genome. The repeat content of the two species would be similar regardless of the continuity of the assembly or how that was achieved. I recommend the authors delete the last sentence in this paragraph.

13. Line 254: "...multi copy..." should be hyphenated (multi-copy).

14. Line 255: "...and 37 not present in one individual." Specify which individual was missing these multi-copy genes (paralogues). Any reason why these 37 multi-copy genes were missing? Lower coverage? Assembly problem?

15. Lines 270-272: "As expected, we see a higher number of singletons in these two individuals..." Here the authors should be more explicit about the discrepancy in the number of singleton SNPs in the two African wild dogs sequenced by Campana et al. 2016 and the three individuals sequenced by the authors. Please provide numbers or percentages about the differences and then cite the Appendix S1 for the detailed methods used for variant calling.
Coverage in and of itself may not be the sole reason for the higher number of singletons in the two African wild dogs sequenced by Campana et al. More stringent filtering methods applied to these two individuals would likely have resulted in a comparable number of SNPs to the three individuals sequenced by the authors. The authors should discuss these alternatives. Also, the Nielsen et al. 2011 and 2012 references are not included in the references (main text or Appendix S1). Also, the authors should consider the following papers: Bryc et al. 2013 Genetics 195: 553 and Kousathanas et al. 2017 Genetics 205: 317.

16. Lines 280-281: "Our estimates show that, while being heavily threatened, African Wild dogs seem to still retain a relatively high within individual heterozygosity." First "Wild" in this sentence should be revised as "wild." Second, the conclusion of "relatively high within individual heterozygosity" is impossible to judge without context to some reference/metric or other species. Relative to what exactly? The per site heterozygosities measured by the authors should be compared to those obtained from other species listed as endangered or critically endangered on the IUCN Red List. The paper by Robinson et al. 2016 Current Biol. 26: 1183 would be of use for this. Furthermore, it would be useful to compare the per site heterozygosities obtained for the three African wild dogs with those of gray wolves reported by Gopalakrishnan et al. 2017 BMC Genomics 18: 495 (see their Table S1).

17. Lines 299-301: "This may indicate that input molecule length is a key factor for scaffolding, while coverage is a key factor for contig assembly." Input molecule length is indeed likely to have a strong effect on assembly quality for the 10X Genomics platform. In fact, this is directly stated by 10X Genomics: "DNA quality. By far the most common cause of subpar assembly results is poor input DNA quality" (https://support.10xgenomics.com/de-novo-assembly/software/pipelines/latest/troubleshooting). In fact, the Chromium library preparation process may nick the DNA and thus cause fragmentation (smaller molecule lengths). The authors should include and cite the weblink above.
It is somewhat surprising that the assemblies of the three African wild dogs were so different in terms of their assembly metrics (e.g., contig and scaffold N50s). Given that the 10X Genomics linked-read technology is still relatively new, it's difficult to judge whether these results are common or not. The authors' findings do not accord with my own experience using 10X, where assembly metrics from multiple individuals of the same species were more consistent (mostly identical). The authors should discuss in in one or two additional sentences other factors that may have influenced their results: 1) sample handling, storage, and/or preparation; 2) library preparation - were the three libraries prepared by the same lab or technician? The authors state in Appendix S1 that the three individuals were sequenced at two different sequencing facilities/vendors; 3) sequencing platforms, chemistries used (HiSeq X for two individuals vs. HiSeq4000 for the third).

18. Lines 357-375: Cost effectiveness: The authors should list the US sequencing facilities examined and their corresponding prices for Chromium library preparation and sequencing in the Supporting Information-Appendix 1 in a table. This will provide readers with the explicit information to gauge different costs associated with these services. This information is also usually provided on the websites of sequencing facilities and vendors. Also, the authors should indicate the pricings for the library preparation and sequencing at the two sequencing facilities they used to generate the data of the three African wild dogs. Also, how much would the cost be for if the authors had used generated and sequenced Illumina shotgun

and mate pair libraries to obtain genome assemblies comparable in quality to those generated using the 10X Chromium platform?

19. Lines 408-411: See my previous comments with respect to this issue in point # 15 above. It would be useful to cite Nielsen et al. 2011 and 2012 here.

20. Line 414: "other threatened large bodied carnivores..." - Neither the Iberian lynx nor dwarf Channel island fox would be considered large-bodied. I suggest the authors revise this just as: "other threatened carnivores..."

21. Line 421: a comma should be added after "dogs" in this sentence.

22. Line 433: "...as part of the assembly process, however, when the fasta consensus sequence..." This is a run-on sentence and should be broken into two sentences: "...as part of the assembly process. However, when the fasta consensus sequence..."

23. Line 473: "DNA was extracted 9 days after the sample was taken." The authors should provide details about how this sample was stored prior to DNA extraction. Also, what type of blood tubes (e.g., Vacutainer) were the samples collected into? These details are important to document given the importance of the HMW input DNA to the success of the 10X Genomics Chromium technology (and in the interests of reproducibility).

24. Line 486 (and in Appendix S1): In the interests of reproducibility, the default assembly parameters should be listed or described.

25. Line 492: "lineage specific" should include a hyphen.

26. Line 496: "BAC end" should include a hyphen.

27. Lines 524-527: The 10X Genomics Supernova assembler outputs four FASTA data files (raw, megabubbles, pseudohap and pseudohap2); see: https://support.10xgenomics.com/de-novo-assembly/software/pipelines/latest/output/generating. Given that there are only two outputs that provide the phased information (pseudohap and pseudohap2), how could this choice for estimating heterozygosity possibly be described a random? In the interests of reproducibility, the authors should indicate which pseudo-haplotype file was used for which individual African wild dog. Also, the authors should at least take one individual (Sister 2, the one with the most continuous assembly) and estimate the heterozygosity from the other pseudo-haplotype file to check that there is no difference in the inferred number of heterozygous sites (this acts as a control).

28. Line 529: The Samtools and Picard programs should be capitalized.

29. Literature cited: The authors should carefully check the formatting of their references so that they consistently conform to the journal standards (e.g., journal titles are often not properly capitalized).

30. Methods (main text and Appendix S1): Samples. Given the requirement of input DNA with long molecule lengths and its importance to the 10X Genomics technology, no details or information is provided on how the HMW genomic DNA was assayed following extraction. This is absolutely crucial and related to the issue of experimental reproducibility. Such HMW DNA is usually assessed using pulse-field electrophoresis techniques or variations thereof. Since the authors used two different sequencing facilities to generate the libraries and sequencing data, different methods may have been used for the assays. In any case, the authors should provide the details about how the HMW DNA was assessed and evaluated prior to Chromium library preparation.

31. Phased assemblies: Even though the percentage of the assemblies that were phased is presented in

Table S1, this feature is never discussed in detail in the main text. However, this is one of the most noteworthy (and marketed) features of the 10X Genomics platform. Phased assemblies also have a dramatic impact on the downstream population genetic analyses and provide additional information for these analyses compared to technologies that do not yield phased assemblies. The authors should include a description of the phasing results of the three African wild dog assemblies in the Data Description & Analyses section as well as discuss this important feature of the 10X Genomics platform.

Methods

Are the methods appropriate to the aims of the study, are they well described, and are necessary controls included? Yes

Conclusions

Are the conclusions adequately supported by the data shown? Yes

Reporting Standards

Does the manuscript adhere to the journal's guidelines on minimum standards of reporting? Yes

Choose an item.

Statistics

Are you able to assess all statistics in the manuscript, including the appropriateness of statistical tests used? Yes, and I have assessed the statistics in my report.

Quality of Written English

Please indicate the quality of language in the manuscript: Acceptable

Declaration of Competing Interests

Please complete a declaration of competing interests, considering the following questions:

- Have you in the past five years received reimbursements, fees, funding, or salary from an organisation that may in any way gain or lose financially from the publication of this manuscript, either now or in the future?
- Do you hold any stocks or shares in an organisation that may in any way gain or lose financially from the publication of this manuscript, either now or in the future?
- Do you hold or are you currently applying for any patents relating to the content of the manuscript?
- Have you received reimbursements, fees, funding, or salary from an organization that holds or has applied for patents relating to the content of the manuscript?

- Do you have any other financial competing interests?
- Do you have any non-financial competing interests in relation to this paper?

If you can answer no to all of the above, write 'I declare that I have no competing interests' below. If your reply is yes to any, please give details below.

I declare that I have no competing interests.

I agree to the open peer review policy of the journal. I understand that my name will be included on my report to the authors and, if the manuscript is accepted for publication, my named report including any attachments I upload will be posted on the website along with the authors' responses. I agree for my report to be made available under an Open Access Creative Commons CC-BY license (http://creativecommons.org/licenses/by/4.0/). I understand that any comments which I do not wish to be included in my named report can be included as confidential comments to the editors, which will not be published.

I agree to the open peer review policy of the journal

To further support our reviewers, we have joined with Publons, where you can gain additional credit to further highlight your hard work (see: https://publons.com/journal/530/gigascience). On publication of this paper, your review will be automatically added to Publons, you can then choose whether or not to claim your Publons credit. I understand this statement.

Yes Choose an item.
