# Supplementary material for: Cost-effective assembly of the African wild dog (Lycaon pictus) genome using linked reads
Source: Gigascience. 2018 Oct 22;8(2):giy124. doi: 10.1093/gigascience/giy124 (PMC6350039; doi:10.1093/gigascience/giy124)
Supplement: Reviewer_3_Report_(Revision_1) -- Klaus-Peter Koepfli, PhD — 6/6/2018 Reviewed [file giy124_reviewer_3_report_(revision_1).pdf]

Reviewer Report

Title: Cost-effective assembly of the African wild dog genome using linked reads.

Version: Revision 1 **Date:** 6/6/2018

Reviewer name: Klaus-Peter Koepfli, PhD

Reviewer Comments to Author:

Re-Review of "Cost-effective assembly of the African wild dog genome using linked reads" by Ellie E Armstrong et al.Manuscript number: GIGA-D-17-00324R1I have re-reviewed the manuscript by Armstrong et al. and find that it has been substantially improved compared to the original submission. The authors have satisfactorily addressed each of the reviewers' comments and revised the manuscript accordingly. The manuscript is very well written, and the data presented and discussion are exciting. I believe this paper provides an important benchmark in conservation genomics with respect to demonstrating the efficacy of generating cost-effective, high-quality genome assemblies for endangered species.The revised manuscript still contains a number of minor grammatical and stylistic errors that the authors should address. I detail these below. Other than these minor corrections, I recommend the manuscript be accepted for publication in GigaScience.1. Lines 194-196. This last part of the sentence is awkwardly constructed (agreement issue). I suggest the following revision: "The mean input DNA molecule length reported by the Supernova assembler was 19.91kb for Sister 1, 196 77.03kb for Sister 2, and 52.00kb for Eureka."2. Line 203: "While the three 10x genomes scaffold N50's..." Again, sentence construction is awkward. The apostrophe from "N50's" should be removed as the "three 10x genomes" are described as a plural, not possessive. This can be clarified by revising the first part of this sentence as follows: "While the scaffold N50s of the three 10x genomes are smaller..."3. Line 214: "The program BUSCO (Benchmarking Universal Copy Orthologs) uses..." should be revised as "The program BUSCO (Benchmarking Universal Single-Copy Orthologs) uses..."4. Line 215: "single copy" should be hyphenated.5. Line 294: "within individual" should be hyphenated.6. "The current version of Supernova 1.2..." 10x Genomics has now released Supernova 2.0. The authors should update the computational spec requirements associated with this version of the assembler.7. Lines 425-426: "In addition, mate-pair libraries require a much quantity of starting material compared to the 10x library prep." This sentence is missing a word. Revise as: "In addition, mate-pair libraries require a much larger quantity of starting material compared to the 10x library prep."8. Line 572: Proteinortho should be capitalized in this sentence.9. Lines 577-579: First, "within individual" should be hyphenated. Second, the sentence is incomplete, as it appears to be missing one or more words.10. Line 579: Add a comma after "Next"11. Line 580: "bwa mem" should changed to "BWA-MEM"12. Line 581: "sam files" and "bam format" should changed to "SAM files" and "BAM format"13. Line 610: "bwa mem" should changed to "BWA-MEM"14. Line 611: "samtools and bcftools" should changed to "SAMtools and BCFtools"15. Line 862: "samtools depth" should changed to "SAMtools depth"16. Table 1: In the row reporting scaffold assembly statistics, the longest scaffold is reported in units of kb, but should be changed to mb.Supporting information-Appendix S1Assembly Quality Assessment section1. "We compare these results to the high-quality canFam3.1 assembly of the domestic dog ([34]; Canis familiaris) and the wolf genome [35]." Include the species name for the wolf in this sentence (Canis lupus).2. In the next sentence, BAC-end should be hyphenated.Repeat Identification and Masking section3. "On the contrary to RepeatMasker," should be revised as "In contrast to Repeatmasker,"Gene annotation section4. The first mention of Proteinortho should be capitalized in this sentence.Variant rates section5. Make sure to revised program titles according to their standard format in the first paragraph: BWA-MEM, BAM format, SAMtools, Picard.6. "We choose a probabilistic over a simple allele counting approach for two reasons." This should be revised as "We chose a probabilistic over a simple allele counting approach for two reasons."7. "However, even if coverage is as high as 55x, heterozygous sites can be falsely called due to erroneous alignment in low-complexity regions or if reads span areas not covered by the reference genome [60]. Showing that even high coverage data could benefit from the application of probabilistic genotype calling." The second sentence here is incomplete, but follows from the

first sentence. Revise as "However, even if coverage is as high as 55x, heterozygous sites can be falsely called due to erroneous alignment in low-complexity regions or if reads span areas not covered by the reference genome [60], showing that even high coverage data could benefit from the application of probabilistic genotype calling."Demographic History section8. "We further filtered this scaffolds by calculating the..." should be changed to "We further filtered these scaffolds by calculating the..."9. "We then mapped the raw reads back to the genome and called the consensus sequence using samtools and bcftools [59,60]." samtools and bcftools should be revised as SAMtools and BCFtools.Table S2: The sample names of the three African wild dogs Sis1, Sis2 and StLouis should be changed to Sister 1, Sister 2 and Eureka so that these are consistent with the sample labels in the main text, and other tables of Appendix S1.

Level of Interest

Please indicate how interesting you found the manuscript: Choose an item.

Quality of Written English

Please indicate the quality of language in the manuscript: Choose an item.

Declaration of Competing Interests

Please complete a declaration of competing interests, considering the following questions:

- Have you in the past five years received reimbursements, fees, funding, or salary from an organisation that may in any way gain or lose financially from the publication of this manuscript, either now or in the future?
- Do you hold any stocks or shares in an organisation that may in any way gain or lose financially from the publication of this manuscript, either now or in the future?
- Do you hold or are you currently applying for any patents relating to the content of the manuscript?
- Have you received reimbursements, fees, funding, or salary from an organization that holds or has applied for patents relating to the content of the manuscript?
- Do you have any other financial competing interests?
- Do you have any non-financial competing interests in relation to this paper?

If you can answer no to all of the above, write 'I declare that I have no competing interests' below. If your reply is yes to any, please give details below.

I declare that I have no competing interests.

I agree to the open peer review policy of the journal. I understand that my name will be included on my report to the authors and, if the manuscript is accepted for publication, my named report including any

attachments I upload will be posted on the website along with the authors' responses. I agree for my report to be made available under an Open Access Creative Commons CC-BY license (http://creativecommons.org/licenses/by/4.0/). I understand that any comments which I do not wish to be included in my named report can be included as confidential comments to the editors, which will not be published.

I agree to the open peer review policy of the journal

To further support our reviewers, we have joined with Publons, where you can gain additional credit to further highlight your hard work (see: https://publons.com/journal/530/gigascience). On publication of this paper, your review will be automatically added to Publons, you can then choose whether or not to claim your Publons credit. I understand this statement.

Yes
